# Systems Modeling of the Water-Energy-Food-Ecosystems Nexus: Insights from a Region Facing Structural Water Scarcity in Southern Spain

**DOI:** 10.1007/s00267-024-02037-6

**Published:** 2024-09-13

**Authors:** Antonio R. Hurtado, Enrique Mesa-Pérez, Julio Berbel

**Affiliations:** 1 Water, Environmental and Agricultural Resources Economics (WEARE) Research Group, Department of Agricultural Economics, Universidad de Córdoba, Campus Rabanales Building C5, 14014 Córdoba, Spain; 2 Departamento de Economía Financiera y Contabilidad, Universidad Loyola Andalucía, 41704 Dos Hermanas (Sevilla), Spain

**Keywords:** WEFE nexus, Water scarcity, Systems thinking, Participatory systems modeling, Network analysis, Mediterranean region.

## Abstract

The complex relationship between water, energy, food, and ecological systems, known as the WEFE nexus, has emerged as a major topic in the debate about sustainable economic development and resource management. This subject is of special interest in Mediterranean coastal areas as rapid economic expansion driven by population growth, higher influx of tourists, and intensification of agriculture is leading to structural water scarcity conditions. However, addressing the diverse range of issues associated with the nexus is a difficult task due to the existence of intricate interconnections, interdependencies, and nonlinearities within and across its various components. Accordingly, this case study applies a combination of participatory systems modeling and network analysis tools to yield insights into the complexity of this nexus in Axarquia, a region with features that make it an example of water-stressed jurisdictions in the Mediterranean. Overall, our results provide a strong foundation to understand the dynamics that govern this nexus in regions where the availability of freshwater resources is a significant concern. Furthermore, they lay the groundwork for the development of models and scenarios to simulate the impact of various policies and interventions on the overall system.

## Introduction

Economic development plays an essential role in the improvement of living standards, but it can also cause sustainability issues related to higher extraction and utilization of resources to support industrial production, infrastructure development, and urbanization (Altintas and Kassouri 2020; Danish et al. 2019; Guo and Shahbaz 2024). Furthermore, economic growth often involves the intensification of agricultural and industrial activities, which can lead to increased environmental pressures associated with pollution, habitat destruction, biodiversity reduction, and reduced long-term viability of ecosystems (Prabhakar 2021). Addressing the sustainability challenges associated with economic development requires the adoption of integrated, holistic approaches to balance economic, social, and environmental objectives. This involves the design of sustainable development strategies that prioritize resource efficiency, environmental conservation, social equity, and resilience, while fostering innovation to support the adoption of sustainable and inclusive economic practices (Morgan et al. 2022; Ulucak and Lin 2017).

In this context, the water-energy-food-ecosystems (WEFE) nexus has emerged as an approach that promotes the notion that water, energy, food, and ecosystems need to be viewed as inextricably interconnected entities with the aim of implementing integrated solutions that lead to the achievement of the United Nations Sustainable Development Goals SDGs (European Commission 2019; UN General Assembly 2015). As the nexus is increasingly affected by factors such as population growth, extreme climate events, or landscape disturbance, among others, its management demands a comprehensive understanding of the interdependence between the components of the system. However, the challenge lies not only in understanding the intricate web of interactions between issues such as water availability, consumption patterns, energy and food production, and ecosystem services, but also in devising strategies that optimize its components while safeguarding the environment and human well-being (Bidoglio et al. 2019; Shah 2023).

Water arguably forms the backbone of the WEFE nexus because it is required to sustain ecosystems, such as wetlands, rivers, and forests, which regulate the water cycle and provide habitat for diverse species. Likewise, agriculture, as the primary source of food production, relies heavily on water, highlighting the nexus’s direct connection to food security. Understanding and managing the role of water within this nexus is vital as it addresses the challenges of resource availability, equitable access, and climate change resilience, while also promoting the conservation of biodiversity and the achievement of sustainable development goals (European Commission 2019; Zhao et al. 2021). In recent years, freshwater scarcity, and the vulnerability of water systems to climate change have become economic and environmental issues of growing concern, which has led the World Economic Forum to include water-related natural disasters and extreme weather events as the second risk that will be faced in the next two years and the third one in a ten-year horizon (WEF 2023). In the Mediterranean region, in particular, concerns about water scarcity are growing, and the topic has become part of the public discussion due to ongoing drought events. In this scenario, Lucca et al. (2023) argue that the holistic and transdisciplinary perspective of the WEFE nexus offers an innovative approach for the development of integrated resource management strategies to address the diverse pillars of sustainability in the region.

Against this backdrop, the objective of our study is to elucidate, through the lens of systems modelling, the complexities of the WEFE nexus in a region of southern Spain that is facing “closed basin” conditions (i.e. all available water resources have been over-allocated), with a view to identifying an initial set of points where interventions could be implemented to improve the prospects of long-term sustainability. Successful environmental policy interventions require understanding the cause-and-effect context in which the target system operates (Castro 2022), and system dynamics studies aim to develop a comprehensive understanding of how different parts of a system interact with each other and their surroundings, using causal loop diagrams (CLDs) as a tool to show the cause-and-effect relationships within it (Forrester 1961; Sterman 2000). Unlike traditional linear analyses that often dissect a problem into isolated components, systems modelling captures the intricate interactions and non-linear dynamics that characterize complex systems, thus making it perfectly suited to transcend the disciplinary boundaries of the nexus, allowing for the identification of unexpected links, the exploration of scenarios, and the generation of information for decision-makers to anticipate and address potential pitfalls (Jackson 2003; Meadows 2009; Richardson 2011).

The application of system dynamics tools to nexus studies has gained increasing momentum in the past decade, driven by the growing recognition of the interconnectedness and complexity of resource systems. Case studies from various regions have demonstrated the effectiveness of these tools for the optimization of resource allocation and the exploration of policy scenarios for sustainable development. Some examples include their use in the modeling of the coevolution process of the water-energy-ecosystem nexus in China’s Hehuang region (Feng et al. 2016); the quantification of the impacts of water resources allocation on the water-energy-food-society nexus in China’s Hanjiang River basin (Zeng et al. 2022); an assessment of the water-energy-food nexus and resource sustainability in Iran’s Ardabil Plain (Javan et al. 2023); the analysis of the water-energy-food nexus for sustainable food production governance in São Paulo, Brazil (Francisco et al. 2023); an analysis of the interactions among numerous components related to water and water-related energy consumption in South Korea (Yoo and Kim 2023); an in-depth analysis of food production within the context of Qatar’s energy-water-food nexus (Shubbar et al. 2024); and the creation of a model to track and manage the water-food-environment-ecosystem nexus in groundwater irrigation districts in China’s Hebei Plain (Dang et al. 2024). Accordingly, this approach is well suited to explore the dynamics that govern the WEFE nexus in regions that are facing basin closure conditions, i.e. a state where all the available resources of a basin or aquifer have been allocated (Falkenmark and Molden 2008; Molle et al. 2010). The results of our research provide valuable insights for other regions in the world facing similar issues.

## Overview of the Study Area

**Fig. 1:**
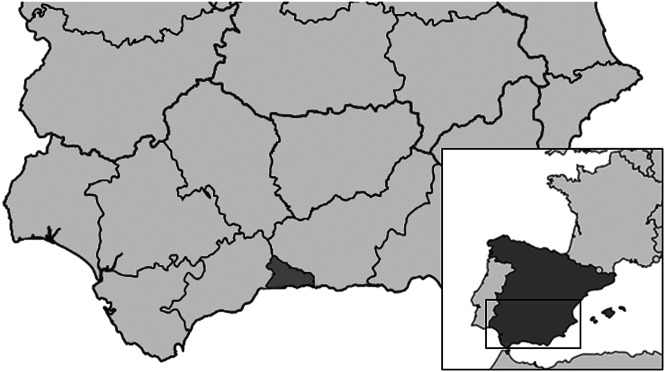
Map of the comarca of Axarquia within Spain (Source: adapted from https://www.mapchart.net/)

The area of our study is Axarquia, a comarca made up of 31 municipalities spanning 1025 km^2^ in the Andalusian province of Malaga, southern Spain (36^o^50’25”N 4^o^06’37”W) (Fig. 1). In Spain, comarcas are associations of municipalities (about 22 in average) that share common geographical, cultural, and economic characteristics. They are an administrative level between provinces (NUTS 3) and municipalities, formed voluntarily to facilitate management and planning of public services, as well as to promote cooperation for economic development. The Axarquia region, known for its distinct geography and cultural heritage, has historical roots dating back to the Moorish period in the Iberian Peninsula, specifically during the time of Al-Andalus.

Axarquia’s unique geography has significantly influenced its three main economic sectors (tourism, construction, and agriculture), making it an excellent illustration of the challenges that water-stressed regions in the Mediterranean are facing when they are confronted with major economic growth opportunities. The region’s mild climate, natural parks and nature reserves, and picturesque coastline and “white villages” have drawn new residents and established it as a year-round tourist destination, whereas the countryside offers optimal conditions for farming activities. At the same time, its semi-arid climate has led to growing sustainability challenges in the past 20 years, driven by a dramatic increase of water demand caused by a combination of rapid population growth (from 143 thousand permanent residents in 1998 to over 220 thousand in 2021) (INE 2024), higher influx of tourists and a growing number of second homes (an average of around 50 thousand seasonal residents throughout the year) (Regional Government of Andalusia 2023a), conversion of pasture and dry land into irrigated land (from 5100 ha in 1999 to almost 13,000 ha in 2021), and an increasing cultivation of very lucrative water-intensive subtropical crops like avocado and mango (from around 4000 ha in 1999 to nearly 9500 ha in 2021) (INE 2022; MAPA 2022; Regional Government of Andalusia 2022, 2023b). These trends have led Axarquia to a chronic structural imbalance between water supply and demand (Hurtado et al. 2024), with the deficit currently estimated to be at around 11 hm^3^/year, i.e. over 10% of total demand (Tocados-Franco et al. 2024). This scenario, combined with long and severe droughts hitting southern Spain since 2018, has placed the region near a state of emergency as the water levels in the comarca’s sole reservoir are currently at 16% of its capacity after falling to below 10% in November 2022 (Regional Government of Andalusia 2024). In addition, the overall state of the local aquifers has deteriorated in recent years, thus suggesting that they are being overexploited (Regional Government of Andalusia 2023a). This condition has led Axarquia to become the subject of several publicly funded projects such as ours to find solutions that could lead to long-term sustainability in the region.

## Methodology

We applied a Group Model Building (GMB) approach to map out the causal relationships, feedback loops, and trade-offs that shape the complexities of the WEFE nexus in an Andalusian water-stressed region (described above), combined with network analysis to identify candidate leverage points where interventions could help drive the system towards long-term sustainability. A description of our methodology follows, covering the dimensions established in the 4P framework for participatory modeling: why was this approach applied? (Purpose), who participated and why? (Partnerships), how were stakeholders involved? (Processes), and what products resulted from these efforts? (Products) (Gray et al. 2018).

### Participatory Systems Modeling

While the general guidelines for systems modeling have remained stable since their conception by Jay Forrester in the late 1950s and early 1960s (Forrester 1961), several methods have been created in the past six decades for the collection and processing of inputs. Among them, Participatory Systems Modeling (PSM) is well suited for our study due to several distinctive advantages. Most remarkably, it fosters a collaborative environment where various perspectives, knowledge domains, and values are integrated, thus helping to capture the nuanced feedback loops, trade-offs, and potential co-benefits that exist within complex systems such as the WEFE nexus. This collaborative engagement also fosters mutual learning among stakeholders, promoting dialogue and cooperation between sectors that often operate in isolation. Likewise, it creates a sense of ownership among stakeholders, as their inputs directly shape the modelling process. This ownership, in turn, enhances the likelihood of successful implementation of resulting policies (Barbrook-Johnson and Penn 2022; Decker and Wendel 2023; Kiraly and Miskolzi 2019). For these reasons, the participatory modelling approach has been increasingly applied to the study of different aspects of water, energy, food, and ecological systems as well as to promote the involvement of different groups of stakeholders in debates related to these issues (Antunes et al. 2015; González-Rosell et al. 2020; Kimmich et al. 2019; Lopes and Videira 2016; Sedlacko et al. 2014).

Three major approaches exist for the application of PSM: group model building (GMB), participatory system dynamics modeling (PSDM), and community-based system dynamics (CBSD) (Table 1). We applied the GMB approach due to its simplicity and our outmost interest in collecting viewpoints and promoting collaboration without much overburden on participants. Furthermore, this approach has the longest history and builds on the premise that qualitative models such as causal loop diagrams (CLDs) are often sufficient to understand a problem and identify solutions (Vennix 1999). The strengths and weaknesses of different participatory systems modeling approaches have been thoroughly described and discussed elsewhere (Decker and Wendel 2023; Kiraly and Miskolczi 2019; Suprun et al. 2018).

**Table 1:** Summary description of participatory system dynamics approaches (adapted from Decker and Wendel 2023; Kiraly and Miskolczi 2019)

	Profile of Participants	Level of Stakeholder Participation	Type of Model Produced
Group Model Building (GMB)	Highly qualified, extensive knowledge and expertise.	Carefully balanced (generally once or twice), ensuring that they contribute effectively without being overburdened.	Qualitative models that can be easily understood and owned by the stakeholders (causal loop diagrams, CLDs).
Participatory System Dynamics Modeling (PSDM)	A combination of highly qualified professionals and community partners.	Active in the early stages of model building as well as in the testing and experimentation with the simulation models produced by experts.	Quantitative (stock and flow) simulation models developed by system dynamics experts.
Community-Based System Dynamics (CBSD)	Members of communities directly impacted by the issues under analysis. Different levels of education.	Flexible, but high involvement throughout the entire process to promote understanding of the problem and empowerment.	Qualitative models (CLDs) that reflect what is important for the community. In the long term, quantitative simulation models developed with active participation of stakeholders.

#### Systems Modeling with Causal Loop Diagrams

In this study we use causal loop diagrams (CLDs), which are visual representations of the cause-and-effect relationships, interconnections, and feedback loops within a system (Forrester 1961; Sterman 2000). These diagrams consist of a set of variables connected by arrows that indicate the direction and type of influence between them, helping visualize how changes in one variable can lead to changes in others. Thus, if two connected variables change in the same direction, the arrow depicting the cause-effect relationship is attributed a positive (+) sign. Conversely, if two connected variables change in opposite directions, the corresponding arrow is assigned a negative (-) sign. The connected variables can, in turn, form feedback loops that either reinforce or balance the system’s behavior. In a “Reinforcing” (or positive) feedback loop, the changes in a variable are amplified and lead to further changes in the same direction, creating a self-reinforcing cycle that can lead to exponential growth or decline in the system’s behavior. In a “Balancing” (or negative) feedback loop, on the other hand, the feedback mechanisms act to counteract the changes that a variable undergoes, thus working to create stability in the system by bringing it back into a state of equilibrium. Feedback loops are categorized as Reinforcing when all the links within it are positive or if it contains an even number of negative connections, and they are regarded as Balancing if they contain an odd number of negative links.

#### Participants, Facilitators, and Modelers

The primary input for our study was collected through a PSM workshop conducted in Axarquia with 26 representatives of fruit and vegetable farmers, irrigators, agri-food processing, agri-food consulting and engineering services, financial services, universities, research organizations, regional government, municipal governments, water technology providers, public water works and utilities, tourism, restauration, real estate, and non-profit organizations. While attendance was just under 50% of the invited individuals, we achieved our goal of engaging a good representation of the major stakeholder groups of the WEFE nexus in the region to capture the highest possible diversity of perspectives (Online Resource 1 – Table SI-1.1).

The facilitation team for the PSM workshop comprised 11 experts in agricultural economics, water economics, water accounting, risk management, sustainability, economic development, innovation systems, socio-ecological systems, water technologies, and environmental technologies. Seven of them were from three Andalusian universities (including the authors of this paper) and four from an environmental consulting firm that is executing projects in the region.

The post-production phase (described below) was conducted by the authors in consultation with subject experts, and a validation session was conducted as part of a second workshop held in Axarquia with 61 stakeholders (Online Resource 1 – Table SI-1.2), many of whom had participated in the first workshop and/or provided inputs during the post-production phase.

#### Participatory Systems Modeling Workshop

The workshop’s preparation and execution followed the methodology recommended by Antunes et al. (2015). The scenario for the co-creation exercise was set through an overview of the current situation of the WEFE nexus in Axarquia, followed by a presentation of the event’s objective and format. The introductory overview was used to present the reference mode, i.e. the patterns and behaviors that key variables of the WEFE nexus in Axarquia (outlined in the “Overview of the Study Area” section above) have exhibited over time. This step helped delineate the system boundaries and develop a shared initial understanding of its behavior. The participants were then divided into two groups (as heterogeneous as possible to ensure a diversity of perspectives) and tasked with identifying key issues affecting water, energy, food, and ecological systems in the comarca, as well as the key variables that, from their viewpoint, are important for the sustainability of the nexus in the region. Thirty-two variables were coincidentally selected by each group, which, after screening out for duplicates, resulted in 56 variables. Each participant was subsequently asked to select two or three variables that they deemed more critical for the system and draw graphs depicting their perception of their behavior-over-time. Complemented by a brief group discussion, this step helped the participants to identify critical variables, articulate their understanding of how they changed over time, explore the relationships between them, and reconcile different perspectives about the system’s behavior. These 56 variables were then distributed according to their relevance for water, energy, food, and ecosystems.

After a break, which was used to collect insights from individual stakeholders, two new groups were formed to work with the variables selected. Based on the features of the variables, one group was assigned the development of a CLD for the food-water-ecosystems nexus and the other was given the same task for the water-energy-ecosystems nexus. As recommended by Barbrook-Johnson and Penn (2022), this approach was adopted to overcome time constraints and simplify what could become an overwhelming task through the creation of parallel submaps that would be brought together later. The groups were then provided with the variables previously identified as relevant for their respective submaps and asked to identify the most important of them in their assigned submap. Predictably, both groups identified “Availability of water resources” as the key variable in their nexus, which was therefore used as the starting point to establish upstream and downstream causal links among all the variables, and adding, removing, or modifying variables as needed. The insights collected during the break (which included personal reflections on the issues discussed up to that point and specific observations or experiences related to the topic) were used to feed the CLD building process and thus enhance the effectiveness of the second half of the session. Once their CLDs were completed, both groups reported back to the plenary, sharing their insights and conclusions with the other participants.

### Post-Production and Analysis of CLDs

Due to the complexity of the overall WEFE system, the primary CLDs obtained during the PSM workshop were subsequently submitted to a thorough post-production phase to ensure their accuracy and effectiveness in the communication of the insights gathered during their creation. This process was carried out in four steps: i) digitalization of the primary CLDs developed by the stakeholders; ii) refinement and reorganization of the primary CLDs to obtain nexus submaps; iii) merging of the submaps to obtain an integrated CLD of the WEFE nexus; and iv) creation of a summary CLD of the WEFE nexus in Axarquia.

In the first step of this stage, the primary CLDs designed during the workshop were converted into a digital format using the Vensim PLE software (Ventana Systems 2023), and all the ensuing processing of the diagrams was carried out with this tool. The primary diagrams were then submitted to a thorough review process that involved the engagement of local stakeholders and domain experts at various times to collect further insights that were subsequently used to validate the variables, links, and feedback loops. Any inconsistencies, inaccuracies, duplications, missing variables, or missing links were identified and addressed during this review phase to ensure that the CLDs represented the dynamics of the system as accurately as possible, using the reference mode described in the “Overview of the Study Area” section as the basis to validate the logic and structure of the CLDs, assessing whether the loops obtained could plausibly produce the historical trends observed, and thus ensure that the qualitative structure of the models was aligned with real-world patterns as perceived by the workshop participants, local stakeholders consulted during this phase, and domain experts with knowledge of the region. During this step, several variables were iteratively added and refined, secondary and redundant variables were eliminated, and new interlinkages were identified. As a result, the number of variables was reduced to 47 and two conceptual CLDs were obtained, one for food-water-ecosystems (FWEco) and another one for water-energy-food (WEF), the latter being derived from the water-energy-ecosystems CLD originally generated by the workshop participants as it was found to be in fact a closer representation of this nexus. The rationale of this approach was the creation of subsections of the full WEFE nexus map to help us gain insights into the system and assess candidate leverage points (Meadows 2009), effectively breaking down what would otherwise be an overwhelmingly large and complex diagram (Barbrook-Johnson and Penn 2022). In the third step, the two nexus submaps were merged into an integrated CLD of the WEFE nexus in Axarquia. The overall post-production phase was carried out over a combined period of approximately six months and involved an onerous iterative process of analysis of the loops obtained and continuous optimization of the models until no errors were found.

As for the analysis of the CLDs, the number of loops for each diagram was calculated using the “Loops” tool of the Vensim software, removing the workbench variables one by one to avoid double-counting, until no variables were left, and repeating the process randomly to ensure that the calculations were correct. On the other hand, a big challenge faced in this stage was the wealth of information contained in virtually every segment of the diagrams. Thus, after careful consideration, for the FWEco and WEF nexus submaps, we opted for prioritizing the identification of the key features of the dynamic behavior of the system and its potential leverage points using a combination of four criteria: (1) variables presenting the highest ubiquity values in these submaps; (2) interactions between the key variables described in the reference mode and other variables in the system; (3) perceived amenability to effective interventions; and (4) main discussions and initiatives that are currently underway or under consideration to address the basin closure process in Axarquia and their potential effects on the reference mode (i.e. on the historical trends observed in the region). The inputs from workshop participants, local stakeholders consulted, and domain experts with knowledge of the region provided the basis for (2), (3) and (4). Conversely, due to its complexity, the integrated CLD was submitted to further processing through network analysis methods as described in the next subsection.

Lastly, a summary CLD was created to capture the key findings, provide a visual illustration of the main conclusions of the study, and facilitate the communication of the results with and among the stakeholders. The results of the modeling process were presented and validated in a session held in Axarquia with 61 representatives of various stakeholder groups (Online Resource 1 – Table SI-1.2).

### Network Analysis of the WEFE CLD

An increasing number of studies suggest that the qualitative insights obtained from the analysis of CLDs of complex systems can be complemented by their combination with quantitative methods, such as network analysis. It is argued that while CLDs excel at helping visualize causal relationships, network analysis can help quantify the strength and direction of these connections, highlighting how changes in one sector can ripple through the system, helping identify potential leverage points that might not be readily apparent in a purely qualitative CLD analysis, and thus enabling the development of more targeted and impactful interventions (Adebiyi and Olabisi 2022; Barbrook-Johnson and Penn 2021; Hoyer et al. 2023; Maruccia et al. 2020; McGlashan et al. 2016; Murphy and Jones 2021; Savi et al. 2021). Accordingly, the next step in our study consisted in the conversion of the WEFE CLD into a directed unweighted graph and the application of network analysis methods to identify potential leverage points.

#### Network Analysis

Network analysis, also known as structural analysis, is a method based on graph theory that is applied in the examination of the structure, behavior, and properties of networks. It is used to obtain insights into the structure and function of a wide range of complex systems, facilitating a deeper understanding of interconnected phenomena and informing strategies for optimization, intervention, and management in various domains (Hevey 2018; Wellman 1983).

The foundations of network analysis lie on the study of graphs formed by nodes and edges. Nodes (also called vertices) represent individual variables within the system, while edges (also called arcs, links, or connections) represent the interactions or relationships between nodes. These interactions can be binary (i.e., present, or absent) or weighted (i.e., having varying degrees of strength or intensity). Networks are formally represented as adjacency matrices *x*_*ij*_ = {0, 1}, where nodes are *x*, and the values 0 and 1 represent the absence or presence of edges between each pair of nodes, respectively. After the graphic representation of a network is built, a set of metrics are used to characterize its topology, i.e. to break down the arrangement of its components and identify key features. These include structural metrics that describe the degree of interconnectedness within the network (density), the average degree of separation between nodes (average path length), the number of edges connected to a node (degree distribution), a node’s relative importance within the network (centrality), and the presence of clusters of nodes (modularity) (Maruccia et al. 2020; McGlashan et al. 2016).

#### Conversion of the WEFE CLD and Network Analysis

The CLD representing the WEFE nexus in Axarquia was represented as a directed unweighted network to allow the application of network analysis. With this purpose, both a list of nodes and a list of edges (Online Resource 4 – Table SI-4.1 and Table SI-4.2) were created and imported into Gephi 0.10, an open-source software for the visualization and exploration of graphs and networks (Bastian et al. 2009). Subsequently, this software was used to obtain network structure and centrality measures through the application of quantitative network analysis techniques, as described by Adebiyi and Olabisi (2022), Maruccia et al. (2020), and McGlashan et al. (2016).

## Results

### Food-Water-Ecosystems (FWEco) Nexus

The submap obtained for the FWEco nexus in Axarquia contained 28 variables and 6454 loops (Online Resource 2 – Fig. SI-2). Remarkably, “Annual water allocations for agriculture” appeared in the largest number of loops, suggesting that it plays a key function in the system. This is indeed the case in Axarquia, where agriculture accounts for approximately 68% of total water use (Regional Government of Andalusia 2023a), making it a good candidate leverage point as it is a variable that can be realistically influenced in a desired way. The next four among the five most ubiquitous variables were “Availability of water resources”, “Consumption of food, goods, and services”, “Profitability of farming activities”, and “Resident population”.

While a CLD containing a large number of loops is generally deemed undesirable because it hinders rather than facilitates understanding about system behavior, the diagram was kept because it reflected the views of the consulted stakeholders about a system that is inherently complex. Subsequently, the CLD was split into two overlapping and interconnected sections (Online Resource 2 – Fig. SI-2) to gain insights about the main features of the nexus and conduct a primary assessment of the most ubiquitous variables for their potential as leverage points.

**Fig. 2:**
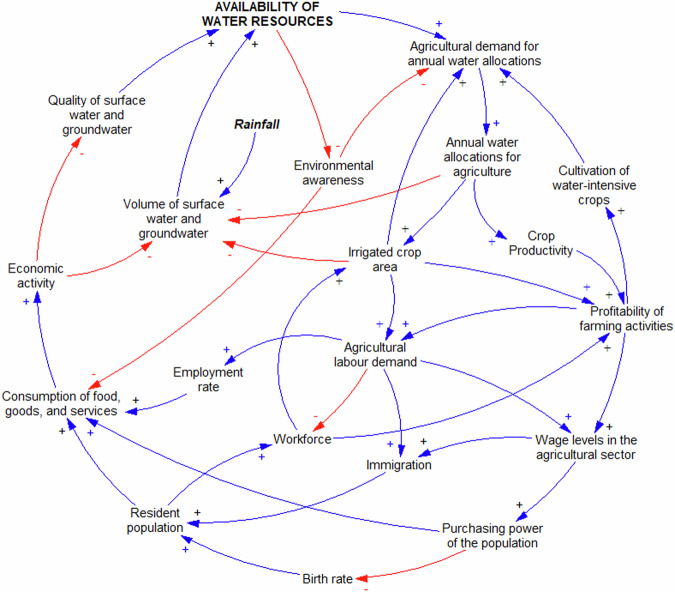
Causal loop diagram displaying the dynamics governing the interactions between agriculture and the economy in Axarquia

As shown in Fig. 2, “Annual water allocations for agriculture” in Axarquia is largely driven by reinforcing feedback loops involving both the conversion from rainfed to irrigated land and a significant increase in the cultivation of water-intensive crops such as avocado and mango (Fajardo-Díaz 2023; Tocados-Franco et al. 2024). These forces have played a key role as major drivers of the region’s economy in recent years, but at the same time are exerting great pressure on the “Availability of water resources” through a series of balancing and reinforcing feedback loops that lead to the reduction of “Volume of surface water and groundwater” and “Quality of surface water and groundwater” in the system. Likewise, the economic success generated by the increase of “Irrigated crop area” and the “Cultivation of subtropical crops” has been one of the drivers of the growth of “Resident population” experienced by Axarquia in the past two decades as well as of higher “Crop productivity” and “Profitability of farming activities”.

**Fig. 3:**
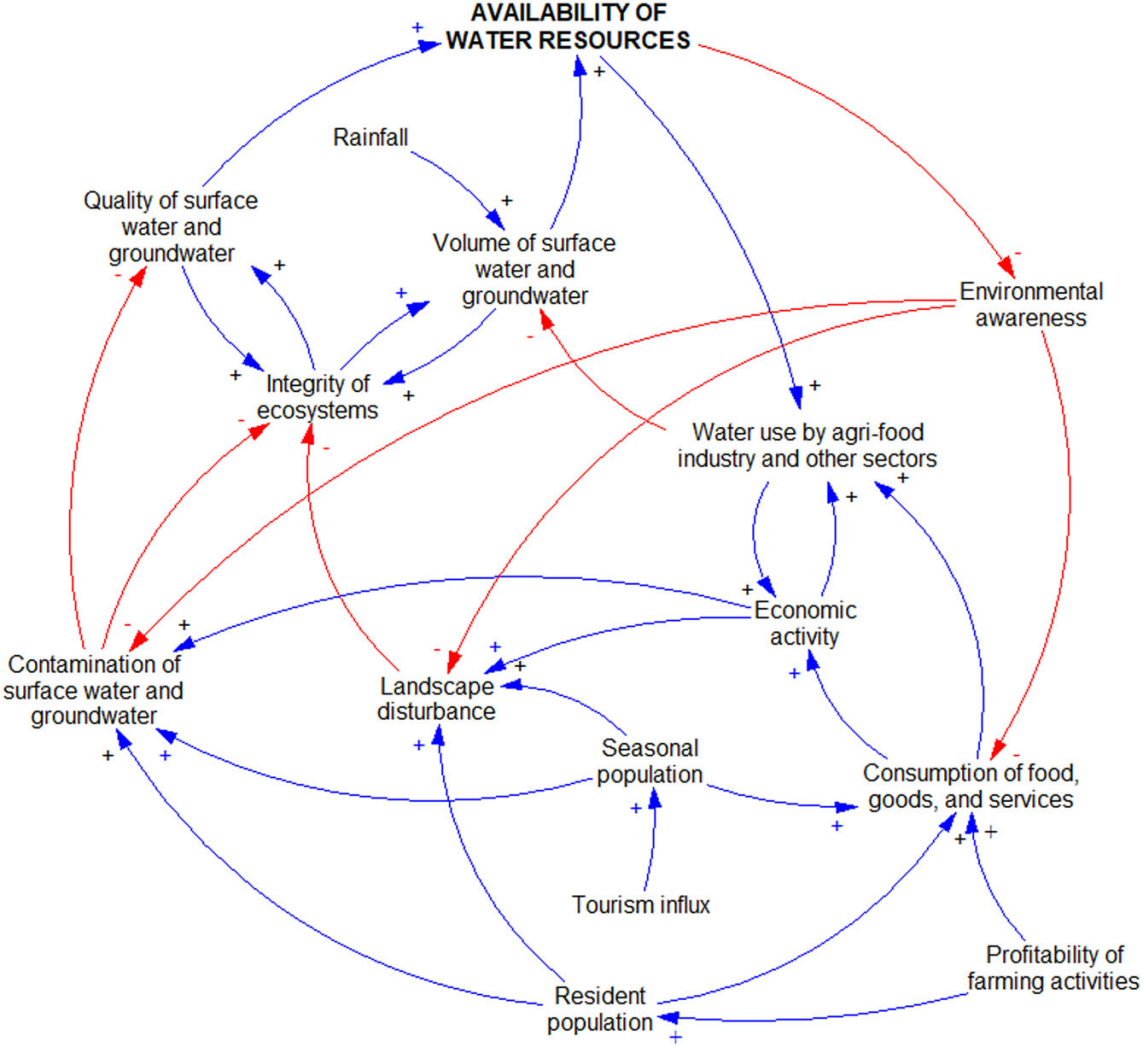
Causal loop diagram displaying the dynamics governing the interactions between population growth and ecosystems in Axarquia

On the other hand, the higher “Profitability of farming activities” that ensues from the “Cultivation of water-intensive crops”, combined with the growth of “Resident population” and the increasing “Tourism influx” to the region, has led to higher “Employment rate”, higher “Purchasing power the population”, more “Consumption of food, goods, and services”, and higher overall “Economic activity”, as shown in Fig. 2 and Fig. 3. All these factors, taken together, end up reducing the “Availability of water resources” through several balancing feedback loops that affect both the “Volume of surface water and groundwater” and “Quality of surface water and groundwater” that is present in the system. Conversely, the decreasing “Availability of water resources” drives an increase of awareness about the natural environment (“Environmental awareness”) (Damania et al. 2017; Liu et al. 2022), which in Axarquia plays an important balancing role in the system through the reduction of ¨Landscape disturbance”, “Contamination of surface water and groundwater”, “Cultivation of water-intensive crops”, “Agricultural demand for annual water allocations”, and overall “Consumption of food, goods, and services” in the local economy (Fig. 2 and Fig. 3).

### Water-Energy-Food (WEF)

The submap obtained for the WEF nexus in Axarquia contained 32 variables and a total of 864 loops. “Availability of water resources” was the most ubiquitous variable, appearing in the largest number of loops, followed by “Environmental awareness”, “Cost of the water cycle”, “Water use by agri-food industry and other sectors”, and “Volume of surface water and groundwater”. Other relevant variables that showed high ubiquity were “Annual water allocations for agriculture”, “Effective regulation and governance”, “Irrigated crop area”, “Agricultural demand for annual water allocations”, and “Agricultural demand for water rights”. As with the previous submap, the challenges related to the large number of loops were overcome by splitting the CLD into two overlapping and interconnected sections (Online Resource 3 – Fig. SI-3), one of which was entirely contained in Fig. 2.

**Fig. 4:**
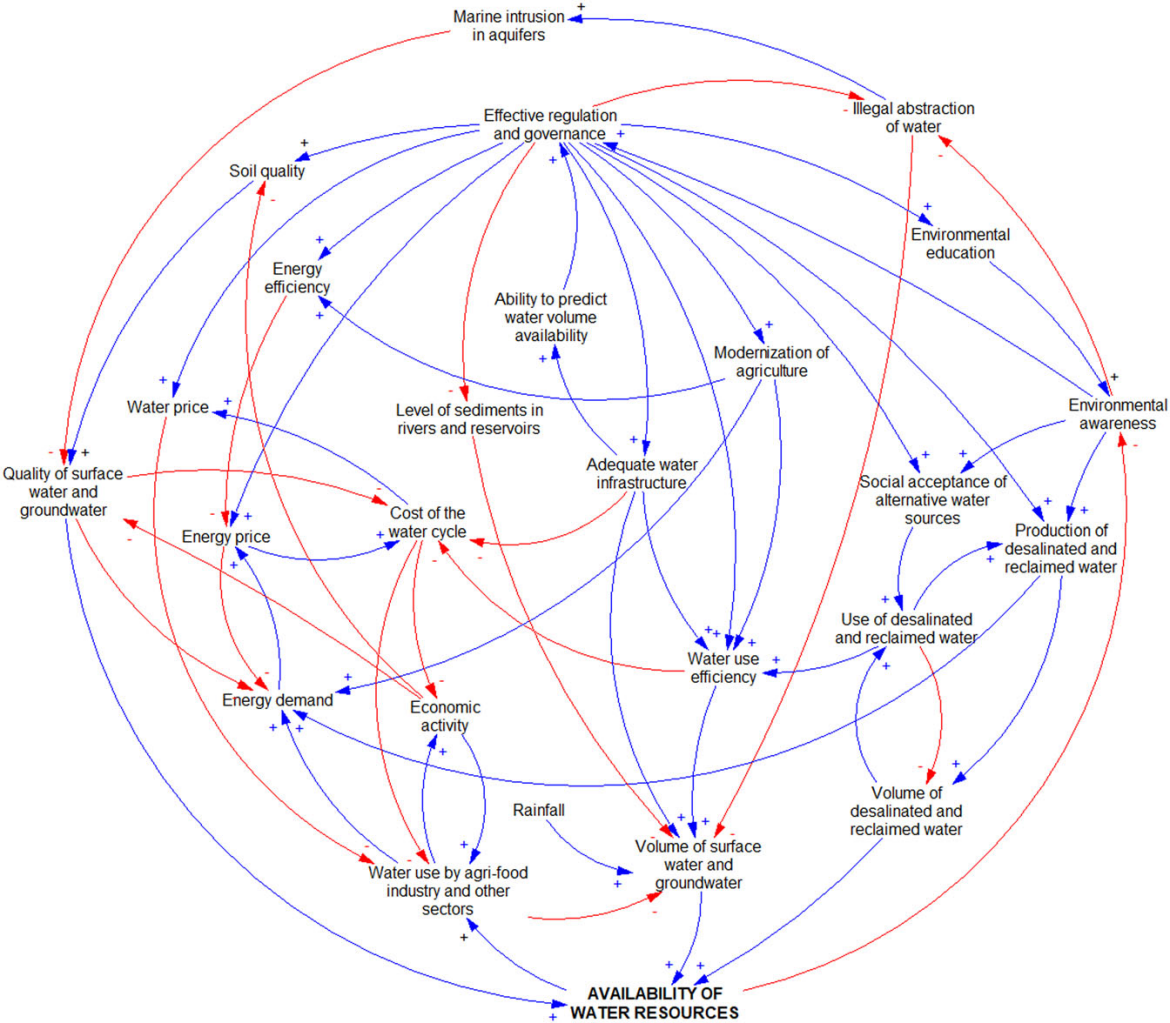
Causal loop diagram displaying the dynamics governing the water-energy system in Axarquia

Figure 4 shows both the key role that the “Availability of water resources” plays as a driver of “Economic activity” and at the same time how, as “Water use by agri-food industry and other sectors” rises, so does “Energy demand”. Moreover, it highlights how the perception of water abundance can potentially have unintended consequences. When the “Availability of water resources” is not a concern, there may be a tendency for some to perceive it as a more abundant resource than it is (Kaur et al. 2023; Morgan and Orr 2015), which can in turn potentially lead to a reduction in “Environmental awareness” and complacency regarding water conservation efforts because individuals may not fully grasp the broader context of water scarcity and its long-term implications (Damania et al. 2017; Praskievicz 2019). As it is observed in Axarquia, low levels of awareness about water stocks can lead to heightened demand from farmers, as they may be inclined to expand their irrigated lands or switch to subtropical crops with the belief that sufficient water is readily accessible. Furthermore, lower “Environmental awareness” due to the lack of concerns about the “Availability of water resources” can potentially lead to a relaxation of regulations and governance surrounding water management. Thus, when water appears plentiful, there may be a tendency to assume that strict oversight and conservation measures are less critical, which can result in less “Effective regulations and governance” as well as in a “Water price” that is below its true economic value (Klümper et al. 2017; Zetland 2009). Additionally, authorities may be less inclined to enforce existing policies or invest in “Adequate water infrastructure”, and at the same time more disposed to grant water rights and “Annual water allocations for agriculture” beyond levels considered sustainable (Fig. 2 and Fig. 4).

“Effective regulations and governance” also play a central function in the WEF nexus due to their critical role in translating “Environmental awareness” into action through laws, policies, guidelines, and frameworks that promote sustainable development and resource conservation. By setting standards and creating incentives for efficient resource use, regulations and governance help prevent resource depletion and minimize environmental harm in the pursuit of economic growth (Ahmed et al. 2021; Porter and van der Linde 1995; Wang et al. 2019) (Fig. 4). “Effective regulations and governance” exert major influence on a key variable of the water-energy system, the “Cost of the water cycle”, not only through “Energy price”, “Adequate water infrastructure”, and “Water use efficiency”, but also through both the “Volume of surface water and groundwater” and the “Quality of surface water and groundwater” that is available in the comarca. In well-functioning markets, regulations and governance measures dictate standards for infrastructure development, impacting initial investment costs but ensuring long-term water supply reliability, and they establish water quality criteria, potentially leading to higher operational expenses for treatment facilities. Likewise, policies and regulations governing water rights, water allocation, pricing and permitting processes have an impact on costs for various users, encouraging conservation and efficient usage. Thus, while compliance with regulations may entail initial expenses, they play a central role in the feedback loops that would lead to the sustainable management of water resources amidst the mounting challenges that affect water-stressed regions like Axarquia.

On the other hand, an increased supply of alternative water sources (“Volume of desalinated and reclaimed water”) has been seen as a solution to scarcity in Axarquia due to the technological advances that have improved the economic feasibility of desalination and reclamation processes, and the important efforts that have been made worldwide to overcome social barriers for their use by dispelling misconceptions and building trust in their safety and efficacy (Angelakis et al. 2018; Jones et al. 2019). While the benefits of the “Use of desalinated and reclaimed water” are clear in terms of addressing water scarcity and ensuring a sustainable water supply (i.e. higher “Availability of water resources”), the “Social acceptance of alternative water sources” has not always been straightforward. For instance, overcoming ingrained cultural attitudes and perceptions regarding the acceptability and safety of using alternative water sources requires education and awareness campaigns (“Environmental awareness”) to inform the public about the rigorous treatment processes and the adherence to stringent quality standards (i.e. that “Effective regulation and governance” is in place) (Adapa et al. 2016; Dolnicar and Schäfer 2009; Gu et al. 2015). Additionally, concerns about the potential impacts on human health and the environment, particularly in the case of reclaimed water, require robust regulatory frameworks to ensure that it meets stringent quality standards, addressing potential concerns about its safety for various uses, including irrigation (European Commission 2016; Shoushtarian and Negahban-Azar 2020). Once these barriers are overcome, the “Social acceptance of alternative water sources” creates a positive feedback loop with far-reaching benefits, beyond lessening the strain on traditional sources and mitigating the impacts of water scarcity. This successful integration encourages further investment and innovation in water desalination and reclamation technologies, ultimately leading to further cost reductions and increasing accessibility, while at the same time increasing “Energy demand” and the overall “Cost of the water cycle”. In view of all their potential benefits, the Regional Government of Andalusia is boosting the supply of alternative water sources in Axarquia from zero in 2021 to 22.5 hm^3^ (approximately 20% of the total) in 2024, which would reduce the pressure on the region’s scarce surface water and groundwater resources (Regional Government of Andalusia 2023c).

### Integrated Water-Energy-Food-Ecosystems (WEFE) Nexus

Merging the two submaps described above yielded a CLD containing all 47 variables identified in this study and, not surprisingly, over 69 thousand causal loops, which underscores once again the vast complexity of the system but hinders the clear visualization of the interconnections that exist among its components (Online Resource 4 – Figure SI-4). Therefore, the application of network analysis methods provided a valuable tool for the exploration of this complex system, to understand information flow within it, reveal structural insights, and highlight potential areas of focus.

As shown in Table 2, the integrated CLD obtained for the WEFE nexus in Axarquia contains 47 nodes and 120 edges. The sparseness of the network (5.6% of the theoretically possible causal connections) suggests that isolated interventions on any given variable would have small effects on the nexus and that actions on multiple leverage points would likely be required to have meaningful impact on the entire system (Adebiyi and Olabisi 2022; Maruccia et al. 2020; McGlashan et al. 2016; Savi et al. 2021). The average path length, on the other hand, indicates that the average number of causal ties between any two nodes is 4.187, suggesting that information is transmitted efficiently across the structure of the network and, therefore, an intervention conducted on one variable can (on average) cause changes in other variables with only a small amount of effort (Maruccia et al. 2020; McGlashan et al. 2016). The combination of low-density and low average path length is typical of “small world” network topologies, which is a feature of most complex networks (Albert and Barabasi 2002).

**Table 2:** Summary of the global network structure of the causal loop diagram displaying the dynamics hypothesized to govern the WEFE nexus in Axarquia

Nodes	Edges	Density	Average Path Length	Number of Clusters	Modularity
47	120	5.6%	4.187	5	0.408

Table 2 also shows that the modularity of the network, estimated with the Girvan-Newman method (Newman and Girvan 2004), is 0.408, which indicates that it contains structural clusters of variables. Taken all together, these results mean that gaining information about central nodes in the WEFE nexus (i.e. variables with high centrality metrics) is key to identify points that hold high potential as effective targets for intervention due to their mediating role between different parts of the system (Maruccia et al. 2020; McGlashan et al. 2016).

**Fig. 5:**
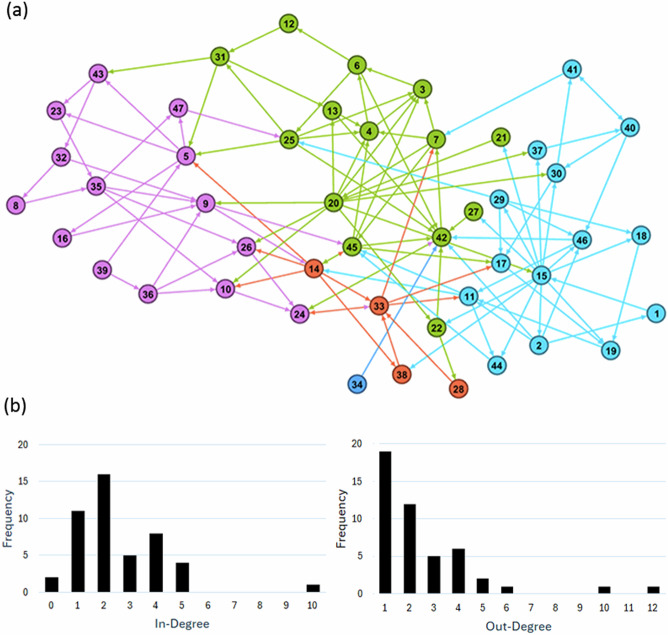
**a** Clusters of nodes and **b** distribution of node in- and out-degree (number of inbound and outbound edges for each node) for the CLD of the WEFE nexus in Axarquia. List of nodes and edges: Online Resource 4 – Table SI-4.1 and Table SI-4.2

The five distinct clusters of variables detected with the Girvan-Newman clustering algorithm are shown in Fig. 5, along with two charts illustrating that most nodes have low in-degree ( ≤ 5) and out-degree (≤6) connections, while only a few larger hubs (nodes with ≥10 in- or out-degree connections) are present in the network. Heavily tailed degree distributions are characteristic of “scale-free networks”, which are well suited for the description of real-world networks (Hein et al. 2006).

The centrality analysis revealed several variables with high centrality scores, indicating their strong potential as leverage points. The results obtained for “Availability of water resources” were disregarded due to its limited analytical value because it is the central variable investigated in this study. As seen in Table 3 and Online Resource 4 – Table SI-4.3, the vertices with the highest degree centrality are related to “Effective regulation and governance” (node 15), “Environmental awareness” (node 20), and the “Volume of surface water and groundwater” (node 42) that is available in the system. These findings highlight that environmental awareness, regulations, and governance are integral components in the complex web of the WEFE nexus. As aforementioned, regulations and governance mechanisms help ensure the preservation of water quality and availability, safeguarding against contamination and over-extraction, and facilitating infrastructure planning to meet societal needs while minimizing environmental impact (Bellamy et al. 2015; Cullet 2012; OECD 2018). Environmental awareness, on the other hand, serves as a catalyst for sustainable development by promoting efficient resource utilization and minimizing waste, aligning societal needs with environmental preservation, fuelling technological innovation, and driving the development of more efficient systems that reduce the environmental footprint (Ghodsvali et al. 2019; United Nations 2022).

**Table 3:** Ranking of the 10 most central vertices in Axarquia’s WEFE nexus

ID	Variable	In-Degree	Out-Degree	Degree Centrality
15	Effective regulation and governance	2	12	14
20	Environmental awareness	2	10	12
42	Volume of surface water and groundwater	10	2	12
45	Water use by agri-food industry and other sectors	5	5	10
14	Economic activity	3	6	9
33	Quality of surface water and groundwater	4	4	8
5	Agricultural labour demand	4	4	8
25	Irrigated crop area	3	5	8
11	Cost of the water cycle	4	3	7
9	Consumption of food, goods, and services	5	2	7

ID	Variable	Closeness Centrality
20	Environmental awareness	0.417
15	Effective regulation and governance	0.398
14	Economic activity	0.319
2	Adequate water infrastructure	0.314
33	Quality of surface water and groundwater	0.309
45	Water use by agri-food industry and other sectors	0.301
21	Environmental education	0.299
25	Irrigated crop area	0.291
1	Ability to predict water volume availability	0.289
41	Volume of desalinated and reclaimed water	0.277

ID	Variable	Eigenvector Centrality
42	Volume of surface water and groundwater	1.00
3	Agricultural demand for annual water allocations	0.86
24	Integrity of ecosystems	0.78
45	Water use by agri-food industry and other sectors	0.69
4	Agricultural demand for water rights	0.68
33	Quality of surface water and groundwater	0.55
17	Energy demand	0.53
6	Annual water allocations for agriculture	0.52
14	Economic activity	0.48
11	Cost of the water cycle	0.38

ID	Variable	Betweenness Centrality
20	Environmental awareness	714
42	Volume of surface water and groundwater	577
14	Economic activity	426
15	Effective regulation and governance	380
33	Quality of surface water and groundwater	348
5	Agricultural labour demand	312
45	Water use by agri-food industry and other sectors	310
11	Cost of the water cycle	296
9	Consumption of food, goods, and services	229
25	Irrigated crop area	200

“Effective regulation and governance” and “Environmental awareness” ranked high also for closeness and betweenness centrality, giving further support for their high potential as intervention points. Closeness centrality is a measure of how close a node is relative to others in the network, therefore indicating which vertices are best placed to transmit information throughout the entire system and thus impact it more rapidly (Maruccia et al. 2020; Schoenenberger and Schenker-Wicki 2015). Betweenness centrality, on the other hand, estimates the number of times a vertex occurs on the shortest path between other vertices, thus indicating the influence that a node can have on the entire system through the control of the flow of information across different parts of the network (Maruccia et al. 2020; Newman 2010; Schoenenberger and Schenker-Wicki 2015).

“Volume of surface water and groundwater” was the highest ranked vertex in terms of eigenvector centrality, which estimates the power and influence that a variable has over the entire system due to the relative importance of the neighboring nodes within the network (Maruccia et al. 2020). This measure, combined with its high degree and betweenness centrality, suggests that this node plays a key role in the WEFE nexus. Indeed, in Axarquia, surface water and groundwater are the largest source of supply (Regional Government of Andalusia 2023a), with “Rainfall” (node 34) standing as a major driver (Fig. 5a and Online Resource 4 – Fig. SI-4).

Other nodes consistently ranked among the ten most influential in terms of degree, closeness, eigenvector, and betweenness centralities in Axarquia’s WEFE nexus, and therefore with high potential as intervention points, included “Water use by agri-food industry and other sectors”, “Quality of surface water and groundwater”, “Economic activity”, “Irrigated crop area”, and “Cost of the water cycle” (Table 3).

## Discussion

CLDs are a valuable tool to gain insights into complex systems. However, when dealing with highly interconnected webs of cause-and-effect relationships, they show limitations that require the application of complementary approaches. One key challenge is that the plethora of interactions that exist in a very complex system like the WEFE nexus can quickly lead to a cluttered and unwieldy graphic representation if it is done in a CLD format, which can in turn result in missing key interactions and overlooking potential leverage points. In this study, we have been able to deal with these limitations and gain a better understanding of the system by combining a qualitative analysis of the nexus submaps followed with the application of network analysis to the overall WEFE nexus CLD.

Among the most noteworthy outputs of our study, the combined application of qualitative and network analysis methods allowed us to identify four variables that could be effective targets for the design of interventions based on the aggregate assessment of their ubiquity, ability to impact the reference mode, amenability to effective interventions, and ongoing discussions and initiatives to address the basin closure process in Axarquia: “Effective regulation and governance”, “Environmental awareness”, “Annual water allocations for agriculture”, and “Use of desalinated and reclaimed water”. Remarkably, while we found a high degree of overlap between the candidate intervention points identified through network analysis and variable ubiquity, especially in the WEF nexus submap, most of these variables were not found to be amenable to effective interventions. Likewise, “Use of desalinated and reclaimed water” was missed by both approaches, although alternative water sources are in fact recognized for their strategic importance to mitigate structural imbalances as well as to enhance the sustainability and resilience of water management systems (Kumar et al. 2016). Altogether, these findings indicate the usefulness of applying network analysis to identify leverage points based on CLDs, while supporting the notion that this tool can have important shortcomings so qualitative expert knowledge should be leveraged to fill in the gaps (Crielaard et al. 2022, 2023).

**Fig. 6:**
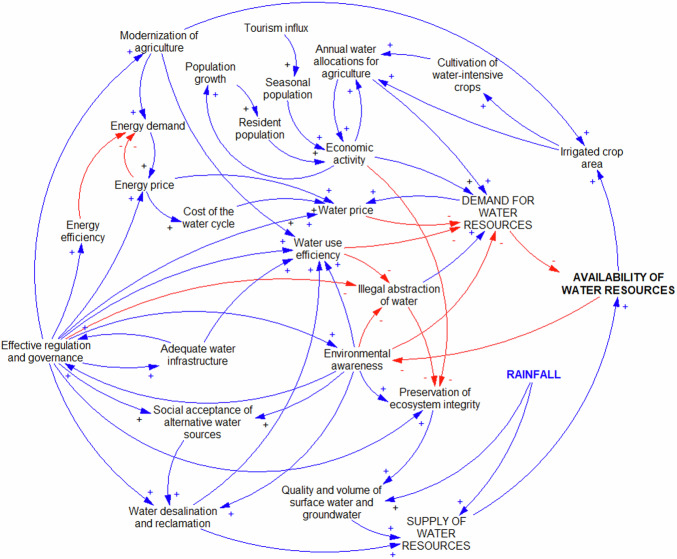
Causal loop diagram summarizing the main cause-effect relationships and causal cycles involved in the WEFE nexus of Axarquia

On the other hand, since one of the main objectives of group processes is to enhance the sense of ownership among stakeholders, we found that the high complexity of the WEFE nexus CLD obtained from the PSM workshop would preclude the achievement of this outcome. Therefore, the insights gained during the post-production and analysis stages of this study were used to develop a summary diagram to synthesize our overall findings and provide a simplified tool for communication with stakeholders. This diagram contains 27 variables and a total of 60 loops, and its core concept is that water availability is driven by the difficult balance between supply and demand in Axarquia (Damania et al. 2017; Meran et al. 2021) (Fig. 6). This summary CLD was validated by stakeholders in the second session held in Axarquia.

According to this diagram, on the supply side, rainfall patterns, surface and groundwater reserves, and alternative water sources are inputs for the region’s water stocks. Their role as central factors in the system is complemented by the need to have adequate infrastructure in place, as precipitation patterns in the comarca present an important challenge given that most of the annual precipitation falls in a few days (Yus Ramos 2005). For the same reason, the preservation of the integrity of the region’s ecosystems is of great relevance to reduce the impact of human activity on the hydrological cycle and thus secure the water resources that are required to meet the various needs of society, agriculture, and industry. Conversely, on the demand side, both the legal and illegal abstraction of water for agriculture and other human activities determine the levels of water resources available in the region. They are complemented by a range of factors that contribute to higher demand, including population growth, tourism influx, economic activity, and changing patterns in agricultural production. Lastly, the interplay between supply and demand is influenced by several variables that have an important balancing role in the system, including environmental awareness, regulations, governance, water price, and water use efficiency.

The dynamics of supply and demand in Axarquia is known to be affected by water scarcity, and the region has faced growing challenges in managing its water resources due to its semi-arid climate and rainfall patterns, which is aggravated by higher demand in recent decades and the recurring periods of drought that affect southern Spain (Yus Ramos 2005). Historically, water policy in Spain has placed significant emphasis on supply-side mechanisms to meet the demands of a rapidly growing population and expanding economic activities. One prominent strategy has been the construction of an extensive network of over 2000 reservoirs and dams, particularly in arid and semi-arid regions, including the La Viñuela dam in Axarquia, which plays an essential role since the early 1990s as the only reservoir of the region (MAPA 2023; Regional Government of Andalusia 2024). Moreover, the country has made substantial strides in advancing desalination and reclamation technologies. Numerous desalination plants have been established along its coastlines, particularly in the water-scarce regions of the southeast (Spanish Desalination and Reuse Association 2023), and planning is underway to build the first one in Axarquia (Jiménez 2023). As for water reclamation, Spain has one of the highest rates of wastewater reuse in Europe (Jodar-Abellan et al. 2019) and the ongoing water crisis has led to the rapid implementation of reclamation infrastructure in Axarquia so that approximately 80% of local wastewater is already treated and reused, which is complemented by the import of reclaimed water from the nearby city of Malaga (Regional Government of Andalusia 2023c).

Nevertheless, relying solely on supply-side strategies is often inadequate for mature water economies and regions with high risk of drought, especially in water-stressed regions like Axarquia where increasing the water supply is challenging and expensive. Accordingly, demand-side tools, such as the promotion of water-saving practices and the support of the adoption of technologies to increase the efficiency of irrigation systems, have been implemented in many water scarce regions such as California, Australia or Spain (Berbel and Esteban 2019), and a detailed analysis of the impact of irrigation water savings on the water-energy nexus has been carried out for Spain (Espinosa-Tasón et al. 2020). Yet, these improvements in water use efficiency may lead to a “rebound effect” unless adequate complementary governance measures are implemented (Berbel and Mateos 2014). This notion is supported by the results of our network analysis, which indicate that the variables in the WEFE nexus are not highly interconnected so it would be necessary to intervene simultaneously at multiple points to generate a cumulative effect that could lead to sustainable system-wide changes. The potential intervention points identified in this study could assist in the development of different types of actions designed with that purpose.

## Conclusions and Future Research

In this paper we reported the combined application of participatory systems modeling and network analysis tools to identify the main components of the WEFE nexus in Axarquia (a semi-arid region in southern Spain), explore how the different variables of the system are interconnected and how changes to one of them could affect others, and to develop a primary list of points where effective interventions could potentially be implemented to promote long-term sustainability within the nexus.

The causal loop diagrams that resulted from the application of the participatory modeling approach provide a valuable visual representation of how the different variables within the system are interconnected in a very complex and dynamic way. Remarkably, the general agreement among the stakeholders consulted during this study was that governance and regulations are at the center of the issues that need to be addressed to ensure the sustainability of water availability within the WEFE nexus in the region, and this perspective was supported by the results obtained through our network analysis. From their viewpoint, the main driver of the challenges faced by the system is the ineffectiveness of the current regulatory and governance landscape, which is not designed to optimize the use of the limited freshwater resources available. This situation is worsened by the drought conditions that frequently affect southern Spain, especially when these periods exceed three years. In this scenario, fostering the production and use of alternative water resources is an adequate solution to address the growing imbalance between supply and demand in the short term, but effective demand-side measures will also be required to ensure long-term sustainability.

On the other hand, from the methodology’s perspective, one of the main conclusions drawn from this study is that the viewpoints collected from stakeholders at participatory group events are only the starting input for the mapping process of very complex systems like the WEFE nexus. The ensuing post-production phase requires significant subject matter expertise as well as thorough and time-consuming efforts in the treatment of the raw information collected during the PSM workshop, especially when the execution of the event has faced time constraints (which is often the case when key stakeholders have limited availability and when brevity is recommended to avoid stakeholder fatigue due to frequent consultations). However, once the post-production phase has concluded, the submaps obtained and a summary diagram can become a powerful tool to facilitate collaboration and communication among stakeholders, as well as to create a shared understanding of the dynamics of the interactions that occur among different variables within the system. Furthermore, we found that the limitations that CLDs may present if used alone in the mapping and analysis of very complex systems such as the WEFE nexus can be overcome through the application of network analysis, as it has been proposed by some authors, to complement the analysis of system submaps.

Overall, our results provide valuable insights and a platform to identify vulnerabilities and potential bottlenecks within the system, from which policymakers, resource managers, and users can develop strategies to mitigate the risks associated with resource scarcity. Moreover, they can be used as the basis for the subsequent development of models and scenarios by researchers and decision-makers to simulate the impacts of various policies and interventions on the overall system, allowing for the exploration of alternative management strategies and the assessment of their consequences. Furthermore, they offer tools to foster stakeholder engagement and collaboration through a shared understanding of the dynamics that govern the WEFE nexus in semi-arid regions, where the availability of freshwater resources is a pressing concern.

For the next phase of this study, our research is focused on conducting an in-depth exploration of the candidate leverage points herein identified, and design interventions that could be implemented to steer the system towards desired outcomes. For this purpose, both hydro-economic models and a database are under development to analyze various scenarios and inform the design of policies for the improvement of long-term sustainability in Axarquia.

## Supplementary information

Appendix 1

Appendix 2

Appendix 3

Appendix 4
