# Supplementary material for: Systems Modeling of the Water-Energy-Food-Ecosystems Nexus: Insights from a Region Facing Structural Water Scarcity in Southern Spain
Source: Environ Manage. 2024 Sep 13;74(6):1045–62. doi: 10.1007/s00267-024-02037-6 (PMC11549115; doi:10.1007/s00267-024-02037-6)
Supplement: Supplementary file 1 — Appendix 1 [file 267_2024_2037_MOESM1_ESM.pdf]

# Environmental Management


# Supplementary Information

# Appendix 1

# Systems Modeling of the Water-Energy-Food-Ecosystems Nexus: Insights from a Region Facing Structural Water-Scarcity in Southern Spain


Antonio R. Hurtado[1,*], Enrique Mesa-Pérez[2], Julio Berbel[1]

[1] Water, Environmental and Agricultural Resources Economics (WEARE) Research Group, Department of Agricultural Economics, University of Cordoba, Campus Rabanales Building C5, 14014 Córdoba, Spain

[2] Departamento de Economía Financiera y Contabilidad, Universidad Loyola Andalucía, 41704 Dos Hermanas (Sevilla), Spain

*Corresponding author (es2rohuc@uco.es)

## Participatory Systems Modeling Workshops

**Table SI-1.1.** Participants in the PSM workshop.

| Sector | Nº of participants |
|---|---|
| Agri-Food Consulting | 1 |
| Andalusia's Regional Administration | 1 |
| Axarquia's Public Administration | 1 |
| Farmers and Food Processors | 8 |
| Financial Services | 1 |
| Irrigation Communities | 1 |
| Non-Profit Organizations | 1 |
| Research Organizations | 4 |
| Restauration, Tourism and Real Estate | 1 |
| Water Technology Providers | 1 |
| Universities | 3 |
| Water Utilities | 3 |
| Total | 26 |

**Table SI-1.2.** Participants in the CLD validation session.

| Sector | Nº of participants |
|---|---|
| Agri-Food Consulting | 1 |
| Analytical Services | 1 |
| Andalusia's Regional Administration | 5 |
| Chemical Industry | 2 |
| Farmers and Food Processors | 30 |
| Irrigation Communities | 9 |
| Municipal Administration | 2 |
| Non-Profit Organizations | 1 |
| Professional Associations | 1 |
| Research Organizations | 5 |
| Technology Providers | 1 |
| Universities | 3 |
| Total | 61 |
