# Supplementary material for: Systems Modeling of the Water-Energy-Food-Ecosystems Nexus: Insights from a Region Facing Structural Water Scarcity in Southern Spain
Source: Environ Manage. 2024 Sep 13;74(6):1045–62. doi: 10.1007/s00267-024-02037-6 (PMC11549115; doi:10.1007/s00267-024-02037-6)
Supplement: Supplementary file 2 — Appendix 2 [file 267_2024_2037_MOESM2_ESM.pdf]

Environmental Management

Supplementary Information

Appendix 2

Systems Modeling of the Water-Energy-Food-Ecosystems Nexus: Insights from a Region Facing Structural Water-Scarcity in Southern Spain

Antonio R. Hurtado[1,*], Enrique Mesa-Pérez[2], Julio Berbel[1]

[1] Water, Environmental and Agricultural Resources Economics (WEARE) Research Group, Department of Agricultural Economics, University of Cordoba, Campus Rabanales Building C5, 14014 Córdoba, Spain

[2] Departamento de Economía Financiera y Contabilidad, Universidad Loyola Andalucía, 41704 Dos Hermanas (Sevilla), Spain

*Corresponding author (es2rohuc@uco.es)

Food-Water-Ecosystem (FWEco) nexus

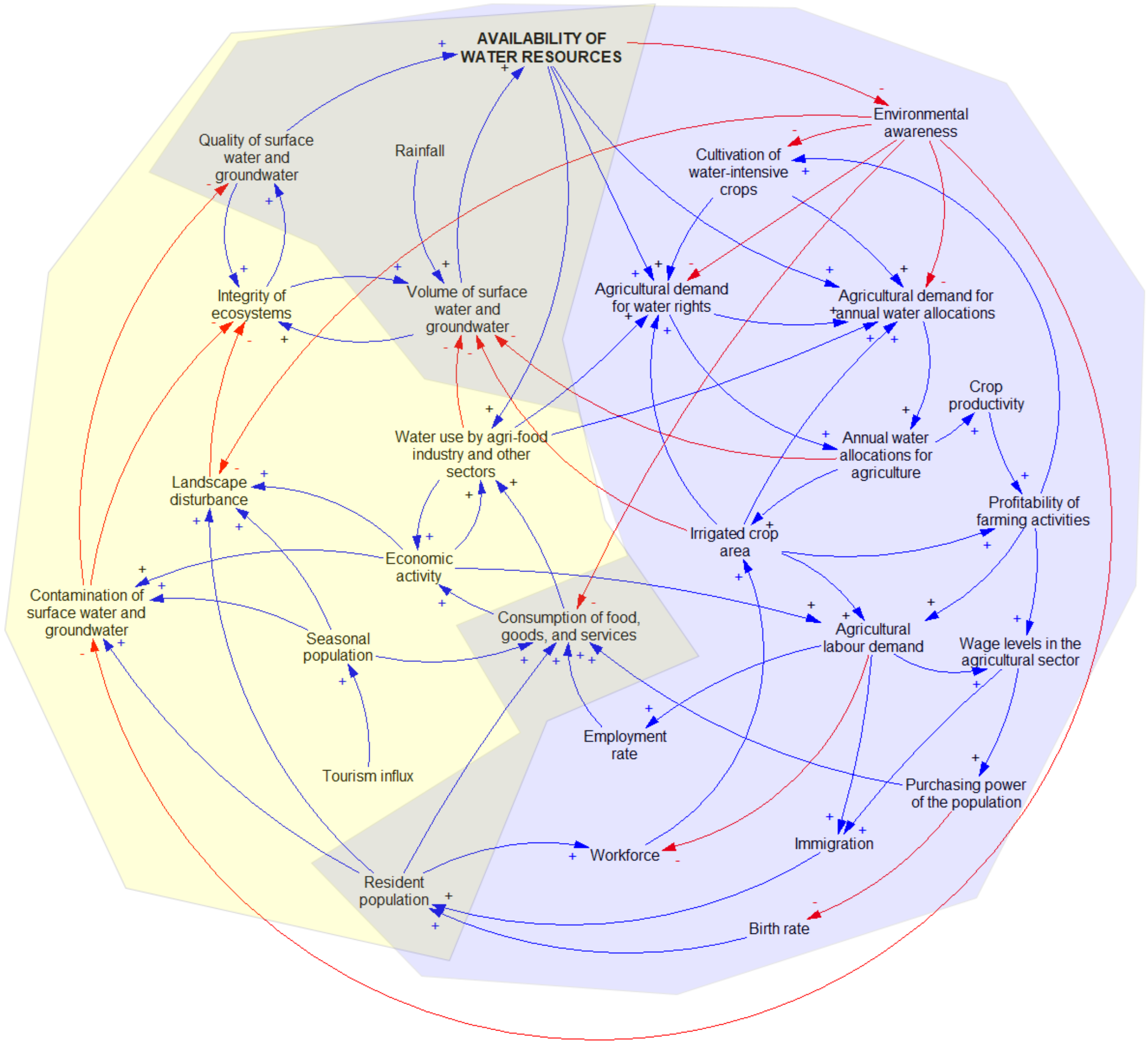

Figure SI-2. Causal loop diagram displaying the dynamics hypothesized to govern the Food-Water-Ecosystem (FWEco) nexus in Axarquía. Blue shade: interactions between agriculture and the economy (**Figure 2** in manuscript); yellow shade: interactions between population growth and ecosystems (**Figure 3** in manuscript).

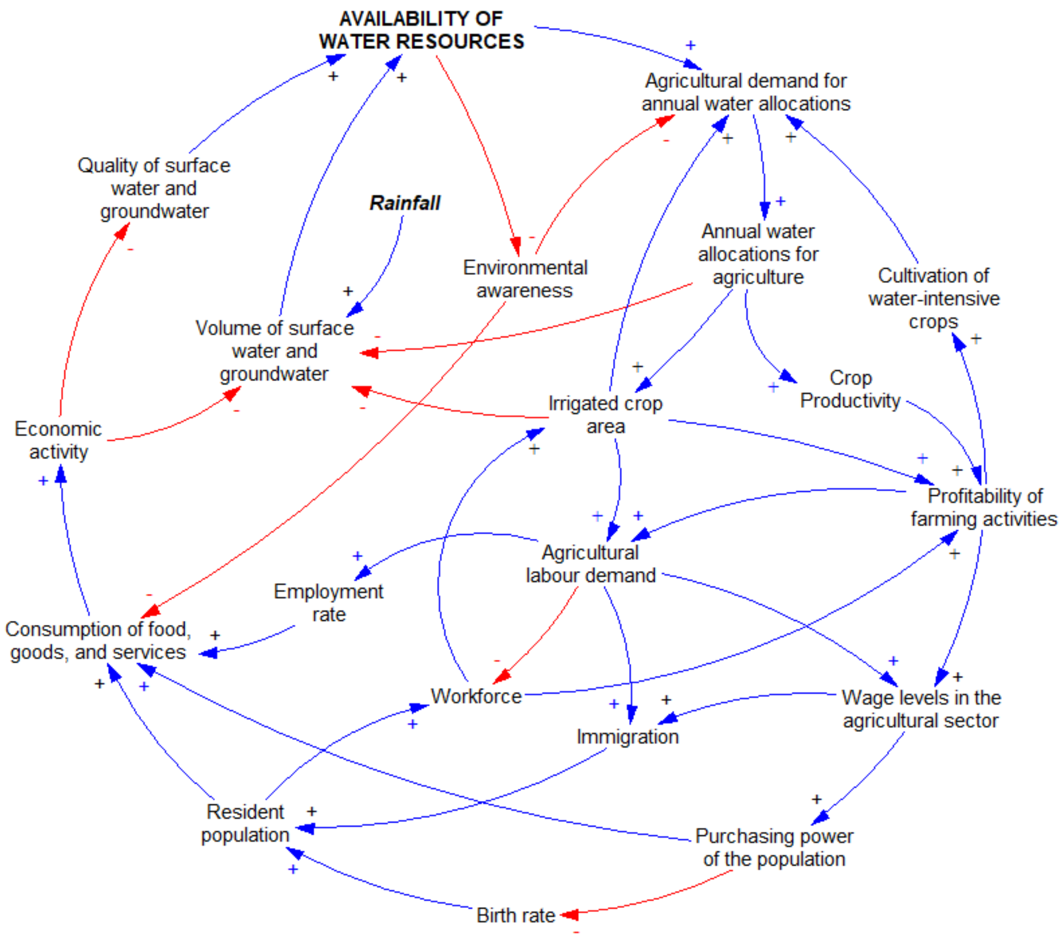

Figure SI-2.1. Causal loop diagram displaying the dynamics governing the interactions between agriculture and the economy in Axarquia (**Figure 2** in manuscript).

Table SI-2.1. Description of the loops that govern the interactions between agriculture and the economy in Axarquia (155 loops)

Nº	Loop
	**Annual water allocations for agriculture – Irrigated crop area**
**RF1** **(Reinforcing)**	Agricultural demand for annual water allocations → Annual water allocations for agriculture → Irrigated crop area → Agricultural demand for annual water allocations
	**Annual water allocations for agriculture – Cultivation of water-intensive crops**
**RF2a** **(Reinforcing)**	Agricultural demand for annual water allocations → Annual water allocations for agriculture → Crop Productivity → Profitability of farming activities → Cultivation of water-intensive crops → Agricultural demand for annual water allocations
**RF2b** **(Reinforcing)**	Agricultural demand for annual water allocations → Annual water allocations for agriculture → Irrigated crop area → Profitability of farming activities → Cultivation of water-intensive crops → Agricultural demand for annual water allocations
	**Volume of Surface Water and Groundwater / Availability of Water Resources**
**BF1** **(Balancing)**	Availability of water resources → Environmental awareness → Consumption of food, goods, and services → Economic activity → Volume of surface water and groundwater → Availability of water resources

**BF2a** **(Balancing)**	Agricultural demand for annual water allocations → Annual water allocations for agriculture → Volume of surface water and groundwater → Availability of water resources → Agricultural demand for annual water allocations
**BF2b** **(Balancing)**	Agricultural demand for annual water allocations → Annual water allocations for agriculture → Volume of surface water and groundwater → Availability of water resources → Environmental awareness → Agricultural demand for annual water allocations
**BF3a** **(Balancing)**	Agricultural demand for annual water allocations → Annual water allocations for agriculture → Irrigated crop area → Volume of surface water and groundwater → Availability of water resources → Agricultural demand for annual water allocations
**BF3b** **(Balancing)**	Agricultural demand for annual water allocations → Annual water allocations for agriculture → Irrigated crop area → Volume of surface water and groundwater → Availability of water resources → Environmental awareness → Agricultural demand for annual water allocations
**BF4a** **(Balancing)**	Agricultural demand for annual water allocations → Annual water allocations for agriculture → Irrigated crop area → Profitability of farming activities → Wage levels in the agricultural sector → Purchasing power of the population → Consumption of food, goods, and services → Economic activity → Volume of surface water and groundwater → Availability of water resources → Agricultural demand for annual water allocations
**BF4b** **(Balancing)**	Agricultural demand for annual water allocations → Annual water allocations for agriculture → Irrigated crop area → Profitability of farming activities → Agricultural labour demand → Employment rate → Consumption of food, goods, and services → Economic activity → Volume of surface water and groundwater → Availability of water resources → Agricultural demand for annual water allocations
**BF4c** **(Balancing)**	Agricultural demand for annual water allocations → Annual water allocations for agriculture → Irrigated crop area → Profitability of farming activities → Agricultural labour demand → Wage levels in the agricultural sector → Purchasing power of the population → Consumption of food, goods, and services → Economic activity → Volume of surface water and groundwater → Availability of water resources → Agricultural demand for annual water allocations
**BF4d** **(Balancing)**	Agricultural demand for annual water allocations → Annual water allocations for agriculture → Irrigated crop area → Profitability of farming activities → Agricultural labour demand → Employment rate → Consumption of food, goods, and services → Economic activity → Volume of surface water and groundwater → Availability of water resources → Environmental awareness → Agricultural demand for annual water allocations
**BF4e** **(Balancing)**	Agricultural demand for annual water allocations → Annual water allocations for agriculture → Irrigated crop area → Profitability of farming activities → Agricultural labour demand → Immigration → Resident population → Consumption of food, goods, and services → Economic activity → Volume of surface water and groundwater → Availability of water resources → Agricultural demand for annual water allocations
**BF4f** **(Balancing)**	Agricultural demand for annual water allocations → Annual water allocations for agriculture → Irrigated crop area → Profitability of farming activities → Wage levels in the agricultural sector → Purchasing power of the population → Consumption of food, goods, and services → Economic activity → Volume of surface water and groundwater → Availability of water resources → Environmental awareness → Agricultural demand for annual water allocations

**BF4g** **(Balancing)**	Agricultural demand for annual water allocations → Annual water allocations for agriculture → Irrigated crop area → Profitability of farming activities → Wage levels in the agricultural sector → Immigration → Resident population → Consumption of food, goods, and services → Economic activity → Volume of surface water and groundwater → Availability of water resources → Agricultural demand for annual water allocations
**BF4h** **(Balancing)**	Agricultural demand for annual water allocations → Annual water allocations for agriculture → Crop Productivity → Profitability of farming activities → Wage levels in the agricultural sector → Immigration → Resident population → Workforce → Irrigated crop area → Volume of surface water and groundwater → Availability of water resources → Agricultural demand for annual water allocations
**BF4i** **(Balancing)**	Agricultural demand for annual water allocations → Annual water allocations for agriculture → Irrigated crop area → Profitability of farming activities → Agricultural labour demand → Wage levels in the agricultural sector → Purchasing power of the population → Consumption of food, goods, and services → Economic activity → Volume of surface water and groundwater → Availability of water resources → Environmental awareness → Agricultural demand for annual water allocations
**BF4j** **(Balancing)**	Agricultural demand for annual water allocations → Annual water allocations for agriculture → Irrigated crop area → Profitability of farming activities → Agricultural labour demand → Immigration → Resident population → Consumption of food, goods, and services → Economic activity → Volume of surface water and groundwater → Availability of water resources → Environmental awareness → Agricultural demand for annual water allocations
**BF4k** **(Balancing)**	Agricultural demand for annual water allocations → Annual water allocations for agriculture → Irrigated crop area → Profitability of farming activities → Agricultural labour demand → Wage levels in the agricultural sector → Immigration → Resident population → Consumption of food, goods, and services → Economic activity → Volume of surface water and groundwater → Availability of water resources → Agricultural demand for annual water allocations
**BF4l** **(Balancing)**	Agricultural demand for annual water allocations → Annual water allocations for agriculture → Irrigated crop area → Profitability of farming activities →Wage levels in the agricultural sector → Immigration → Resident population → Consumption of food, goods, and services → Economic activity → Volume of surface water and groundwater → Availability of water resources → Environmental awareness → Agricultural demand for annual water allocations
**BF4m** **(Balancing)**	Agricultural demand for annual water allocations → Annual water allocations for agriculture → Irrigated crop area → Profitability of farming activities → Agricultural labour demand → Wage levels in the agricultural sector → Immigration → Resident population → Consumption of food, goods, and services → Economic activity → Volume of surface water and groundwater → Availability of water resources → Environmental awareness → Agricultural demand for annual water allocations
**RF3a** **(Reinforcing)**	Agricultural demand for annual water allocations → Annual water allocations for agriculture → Irrigated crop area → Profitability of farming activities → Wage levels in the agricultural sector → Purchasing power of the population → Birth rate → Resident population → Consumption of food, goods, and services → Economic activity → Volume of surface water and groundwater → Availability of water resources → Agricultural demand for annual water allocations

**RF3b (Reinforcing)**	Agricultural demand for annual water allocations → Annual water allocations for agriculture → Irrigated crop area → Profitability of farming activities → Wage levels in the agricultural sector → Purchasing power of the population → Birth rate → Resident population → Consumption of food, goods, and services → Economic activity → Volume of surface water and groundwater → Availability of water resources → Environmental awareness → Agricultural demand for annual water allocations
**RF3c (Reinforcing)**	Agricultural demand for annual water allocations → Annual water allocations for agriculture → Irrigated crop area → Profitability of farming activities → Agricultural labour demand → Wage levels in the agricultural sector → Purchasing power of the population → Birth rate → Resident population → Consumption of food, goods, and services → Economic activity → Volume of surface water and groundwater → Availability of water resources → Agricultural demand for annual water allocations
**RF3d (Reinforcing)**	Agricultural demand for annual water allocations → Annual water allocations for agriculture → Irrigated crop area → Profitability of farming activities → Agricultural labour demand → Wage levels in the agricultural sector → Purchasing power of the population → Birth rate → Resident population → Consumption of food, goods, and services → Economic activity → Volume of surface water and groundwater → Availability of water resources → Environmental awareness → Agricultural demand for annual water allocations
**BF5a (Balancing)**	Agricultural demand for annual water allocations → Annual water allocations for agriculture → Irrigated crop area → Agricultural labour demand → Employment rate → Consumption of food, goods, and services → Economic activity → Volume of surface water and groundwater → Availability of water resources → Agricultural demand for annual water allocations
**BF5b (Balancing)**	Agricultural demand for annual water allocations → Annual water allocations for agriculture → Irrigated crop area → Agricultural labour demand → Wage levels in the agricultural sector → Purchasing power of the population → Consumption of food, goods, and services → Economic activity → Volume of surface water and groundwater → Availability of water resources → Agricultural demand for annual water allocations
**BF5c (Balancing)**	Agricultural demand for annual water allocations → Annual water allocations for agriculture → Irrigated crop area → Agricultural labour demand → Employment rate → Consumption of food, goods, and services → Economic activity → Volume of surface water and groundwater → Availability of water resources → Environmental awareness → Agricultural demand for annual water allocations
**BF5d (Balancing)**	Agricultural demand for annual water allocations → Annual water allocations for agriculture → Irrigated crop area → Agricultural labour demand → Immigration → Resident population → Consumption of food, goods, and services → Economic activity → Volume of surface water and groundwater → Availability of water resources → Agricultural demand for annual water allocations
**BF5e (Balancing)**	Agricultural demand for annual water allocations → Annual water allocations for agriculture → Irrigated crop area → Agricultural labour demand → Wage levels in the agricultural sector → Immigration → Resident population → Consumption of food, goods, and services → Economic activity → Volume of surface water and groundwater → Availability of water resources → Agricultural demand for annual water allocations

**BF5f** **(Balancing)**	Agricultural demand for annual water allocations → Annual water allocations for agriculture → Irrigated crop area → Agricultural labour demand → Wage levels in the agricultural sector → Purchasing power of the population → Consumption of food, goods, and services → Economic activity → Volume of surface water and groundwater → Availability of water resources → Environmental awareness → Agricultural demand for annual water allocations
**BF5g** **(Balancing)**	Agricultural demand for annual water allocations → Annual water allocations for agriculture → Irrigated crop area → Agricultural labour demand → Immigration → Resident population → Consumption of food, goods, and services → Economic activity → Volume of surface water and groundwater → Availability of water resources → Environmental awareness → Agricultural demand for annual water allocations
**BF5h** **(Balancing)**	Agricultural demand for annual water allocations → Annual water allocations for agriculture → Irrigated crop area → Agricultural labour demand → Wage levels in the agricultural sector → Immigration → Resident population → Consumption of food, goods, and services → Economic activity → Volume of surface water and groundwater → Availability of water resources → Environmental awareness → Agricultural demand for annual water allocations
**BF5i** **(Balancing)**	Agricultural demand for annual water allocations → Annual water allocations for agriculture → Irrigated crop area → Agricultural labour demand → Workforce → Profitability of farming activities → Wage levels in the agricultural sector → Purchasing power of the population → Birth rate → Resident population → Consumption of food, goods, and services → Economic activity → Volume of surface water and groundwater → Availability of water resources → Agricultural demand for annual water allocations
**BF5j** **(Balancing)**	Agricultural demand for annual water allocations → Annual water allocations for agriculture → Irrigated crop area → Agricultural labour demand → Immigration → Resident population → Workforce → Profitability of farming activities → Wage levels in the agricultural sector → Purchasing power of the population → Consumption of food, goods, and services → Economic activity → Volume of surface water and groundwater → Availability of water resources → Agricultural demand for annual water allocations
**BF5k** **(Balancing)**	Agricultural demand for annual water allocations → Annual water allocations for agriculture → Irrigated crop area → Agricultural labour demand → Workforce → Profitability of farming activities → Wage levels in the agricultural sector → Purchasing power of the population → Birth rate → Resident population → Consumption of food, goods, and services → Economic activity → Volume of surface water and groundwater → Availability of water resources → Environmental awareness → Agricultural demand for annual water allocations
**BF5l** **(Balancing)**	Agricultural demand for annual water allocations → Annual water allocations for agriculture → Irrigated crop area → Agricultural labour demand → Immigration → Resident population → Workforce → Profitability of farming activities → Wage levels in the agricultural sector → Purchasing power of the population → Consumption of food, goods, and services → Economic activity → Volume of surface water and groundwater → Availability of water resources → Environmental awareness → Agricultural demand for annual water allocations
**RF4a** **(Reinforcing)**	Agricultural demand for annual water allocations → Annual water allocations for agriculture → Irrigated crop area → Agricultural labour demand → Wage levels in the agricultural sector → Purchasing power of the population → Birth rate → Resident population → Consumption of food, goods, and services → Economic activity → Volume of surface water and groundwater → Availability of water resources → Agricultural demand for annual water allocations

**RF4b (Reinforcing)**	Agricultural demand for annual water allocations → Annual water allocations for agriculture → Irrigated crop area → Agricultural labour demand → Workforce → Profitability of farming activities → Wage levels in the agricultural sector → Purchasing power of the population → Consumption of food, goods, and services → Economic activity → Volume of surface water and groundwater → Availability of water resources → Agricultural demand for annual water allocations
**RF4c (Reinforcing)**	Agricultural demand for annual water allocations → Annual water allocations for agriculture → Irrigated crop area → Agricultural labour demand → Workforce → Profitability of farming activities → Wage levels in the agricultural sector → Purchasing power of the population → Consumption of food, goods, and services → Economic activity → Volume of surface water and groundwater → Availability of water resources → Environmental awareness → Agricultural demand for annual water allocations
**RF4d (Reinforcing)**	Agricultural demand for annual water allocations → Annual water allocations for agriculture → Irrigated crop area → Agricultural labour demand → Wage levels in the agricultural sector → Purchasing power of the population → Birth rate → Resident population → Consumption of food, goods, and services → Economic activity → Volume of surface water and groundwater → Availability of water resources → Environmental awareness → Agricultural demand for annual water allocations
**RF4e (Reinforcing)**	Agricultural demand for annual water allocations → Annual water allocations for agriculture → Irrigated crop area → Agricultural labour demand → Workforce → Profitability of farming activities → Wage levels in the agricultural sector → Immigration → Resident population → Consumption of food, goods, and services → Economic activity → Volume of surface water and groundwater → Availability of water resources → Agricultural demand for annual water allocations
**RF4f (Reinforcing)**	Agricultural demand for annual water allocations → Annual water allocations for agriculture → Irrigated crop area → Agricultural labour demand → Workforce → Profitability of farming activities → Wage levels in the agricultural sector → Immigration → Resident population → Consumption of food, goods, and services → Economic activity → Volume of surface water and groundwater → Availability of water resources → Environmental awareness → Agricultural demand for annual water allocations
**BF6a (Balancing)**	Agricultural demand for annual water allocations → Annual water allocations for agriculture → Crop Productivity → Profitability of farming activities → Wage levels in the agricultural sector → Purchasing power of the population → Consumption of food, goods, and services → Economic activity → Volume of surface water and groundwater → Availability of water resources → Agricultural demand for annual water allocations
**BF6b (Balancing)**	Agricultural demand for annual water allocations → Annual water allocations for agriculture → Crop Productivity → Profitability of farming activities → Agricultural labour demand → Employment rate → Consumption of food, goods, and services → Economic activity → Volume of surface water and groundwater → Availability of water resources → Agricultural demand for annual water allocations
**BF6c (Balancing)**	Agricultural demand for annual water allocations → Annual water allocations for agriculture → Crop Productivity → Profitability of farming activities → Wage levels in the agricultural sector → Purchasing power of the population → Consumption of food, goods, and services → Economic activity → Volume of surface water and groundwater → Availability of water resources → Environmental awareness → Agricultural demand for annual water allocations

**BF6d** **(Balancing)**	Agricultural demand for annual water allocations → Annual water allocations for agriculture → Crop Productivity → Profitability of farming activities → Agricultural labour demand → Wage levels in the agricultural sector → Purchasing power of the population → Consumption of food, goods, and services → Economic activity → Volume of surface water and groundwater → Availability of water resources → Agricultural demand for annual water allocations
**BF6e** **(Balancing)**	Agricultural demand for annual water allocations → Annual water allocations for agriculture → Crop Productivity → Profitability of farming activities → Agricultural labour demand → Immigration → Resident population → Workforce → Irrigated crop area → Volume of surface water and groundwater → Availability of water resources → Agricultural demand for annual water allocations
**BF6f** **(Balancing)**	Agricultural demand for annual water allocations → Annual water allocations for agriculture → Crop Productivity → Profitability of farming activities → Agricultural labour demand → Immigration → Resident population → Consumption of food, goods, and services → Economic activity → Volume of surface water and groundwater → Availability of water resources → Agricultural demand for annual water allocations
**BF6g** **(Balancing)**	Agricultural demand for annual water allocations → Annual water allocations for agriculture → Crop Productivity → Profitability of farming activities → Agricultural labour demand → Employment rate → Consumption of food, goods, and services → Economic activity → Volume of surface water and groundwater → Availability of water resources → Environmental awareness → Agricultural demand for annual water allocations
**BF6h** **(Balancing)**	Agricultural demand for annual water allocations → Annual water allocations for agriculture → Crop Productivity → Profitability of farming activities → Wage levels in the agricultural sector → Immigration → Resident population → Consumption of food, goods, and services → Economic activity → Volume of surface water and groundwater → Availability of water resources → Agricultural demand for annual water allocations
**BF6i** **(Balancing)**	Agricultural demand for annual water allocations → Annual water allocations for agriculture → Crop Productivity → Profitability of farming activities → Agricultural labour demand → Wage levels in the agricultural sector → Immigration → Resident population → Consumption of food, goods, and services → Economic activity → Volume of surface water and groundwater → Availability of water resources → Agricultural demand for annual water allocations
**BF6j** **(Balancing)**	Agricultural demand for annual water allocations → Annual water allocations for agriculture → Crop Productivity → Profitability of farming activities → Agricultural labour demand → Wage levels in the agricultural sector → Purchasing power of the population → Consumption of food, goods, and services → Economic activity → Volume of surface water and groundwater → Availability of water resources → Environmental awareness → Agricultural demand for annual water allocations
**BF6k** **(Balancing)**	Agricultural demand for annual water allocations → Annual water allocations for agriculture → Crop Productivity → Profitability of farming activities → Agricultural labour demand → Immigration → Resident population → Consumption of food, goods, and services → Economic activity → Volume of surface water and groundwater → Availability of water resources → Environmental awareness → Agricultural demand for annual water allocations

**BF6l** **(Balancing)**	Agricultural demand for annual water allocations → Annual water allocations for agriculture → Crop Productivity → Profitability of farming activities → Wage levels in the agricultural sector → Immigration → Resident population → Workforce → Irrigated crop area → Volume of surface water and groundwater → Availability of water resources → Environmental awareness → Agricultural demand for annual water allocations
**BF6m** **(Balancing)**	Agricultural demand for annual water allocations → Annual water allocations for agriculture → Crop Productivity → Profitability of farming activities → Agricultural labour demand → Wage levels in the agricultural sector → Immigration → Resident population → Workforce → Irrigated crop area → Volume of surface water and groundwater → Availability of water resources → Agricultural demand for annual water allocations
**BF6n** **(Balancing)**	Agricultural demand for annual water allocations → Annual water allocations for agriculture → Crop Productivity → Profitability of farming activities → Agricultural labour demand → Immigration → Resident population → Workforce → Irrigated crop area → Volume of surface water and groundwater → Availability of water resources → Environmental awareness → Agricultural demand for annual water allocations
**BF6o** **(Balancing)**	Agricultural demand for annual water allocations → Annual water allocations for agriculture → Crop Productivity → Profitability of farming activities → Wage levels in the agricultural sector → Immigration → Resident population → Consumption of food, goods, and services → Economic activity → Volume of surface water and groundwater → Availability of water resources → Environmental awareness → Agricultural demand for annual water allocations
**BF6p** **(Balancing)**	Agricultural demand for annual water allocations → Annual water allocations for agriculture → Crop Productivity → Profitability of farming activities → Agricultural labour demand → Wage levels in the agricultural sector → Immigration → Resident population → Workforce → Irrigated crop area → Volume of surface water and groundwater → Availability of water resources → Environmental awareness → Agricultural demand for annual water allocations
**BF6q** **(Balancing)**	Agricultural demand for annual water allocations → Annual water allocations for agriculture → Crop Productivity → Profitability of farming activities → Agricultural labour demand → Wage levels in the agricultural sector → Immigration → Resident population → Consumption of food, goods, and services → Economic activity → Volume of surface water and groundwater → Availability of water resources → Environmental awareness → Agricultural demand for annual water allocations
**BF6r** **(Balancing)**	Agricultural demand for annual water allocations → Annual water allocations for agriculture → Crop Productivity → Profitability of farming activities → Wage levels in the agricultural sector → Immigration → Resident population → Workforce → Irrigated crop area → Agricultural labour demand → Employment rate → Consumption of food, goods, and services → Economic activity → Volume of surface water and groundwater → Availability of water resources → Agricultural demand for annual water allocations
**BF6s** **(Balancing)**	Agricultural demand for annual water allocations → Annual water allocations for agriculture → Crop Productivity → Profitability of farming activities → Wage levels in the agricultural sector → Immigration → Resident population → Workforce → Irrigated crop area → Agricultural labour demand → Employment rate → Consumption of food, goods, and services → Economic activity → Volume of surface water and groundwater → Availability of water resources → Environmental awareness → Agricultural demand for annual water allocations

**RF5a** **(Reinforcing)**	Agricultural demand for annual water allocations → Annual water allocations for agriculture → Crop Productivity → Profitability of farming activities → Agricultural labour demand → Workforce → Irrigated crop area → Volume of surface water and groundwater → Availability of water resources → Agricultural demand for annual water allocations
**RF5b** **(Reinforcing)**	Agricultural demand for annual water allocations → Annual water allocations for agriculture → Crop Productivity → Profitability of farming activities → Agricultural labour demand → Workforce → Irrigated crop area → Volume of surface water and groundwater → Availability of water resources → Environmental awareness → Agricultural demand for annual water allocations
**RF5c** **(Reinforcing)**	Agricultural demand for annual water allocations → Annual water allocations for agriculture → Crop Productivity → Profitability of farming activities → Wage levels in the agricultural sector → Purchasing power of the population → Birth rate → Resident population → Consumption of food, goods, and services → Economic activity → Volume of surface water and groundwater → Availability of water resources → Agricultural demand for annual water allocations
**RF5d** **(Reinforcing)**	Agricultural demand for annual water allocations → Annual water allocations for agriculture → Crop Productivity → Profitability of farming activities → Wage levels in the agricultural sector → Purchasing power of the population → Birth rate → Resident population → Workforce → Irrigated crop area → Volume of surface water and groundwater → Availability of water resources → Agricultural demand for annual water allocations
**RF5e** **(Reinforcing)**	Agricultural demand for annual water allocations → Annual water allocations for agriculture → Crop Productivity → Profitability of farming activities → Wage levels in the agricultural sector → Purchasing power of the population → Birth rate → Resident population → Consumption of food, goods, and services → Economic activity → Volume of surface water and groundwater → Availability of water resources → Environmental awareness → Agricultural demand for annual water allocations
**RF5f** **(Reinforcing)**	Agricultural demand for annual water allocations → Annual water allocations for agriculture → Crop Productivity → Profitability of farming activities → Wage levels in the agricultural sector → Purchasing power of the population → Birth rate → Resident population → Workforce → Irrigated crop area → Volume of surface water and groundwater → Availability of water resources → Environmental awareness → Agricultural demand for annual water allocations
**RF5g** **(Reinforcing)**	Agricultural demand for annual water allocations → Annual water allocations for agriculture → Crop Productivity → Profitability of farming activities → Agricultural labour demand → Wage levels in the agricultural sector → Purchasing power of the population → Birth rate → Resident population → Workforce → Irrigated crop area → Volume of surface water and groundwater → Availability of water resources → Agricultural demand for annual water allocations
**RF5h** **(Reinforcing)**	Agricultural demand for annual water allocations → Annual water allocations for agriculture → Crop Productivity → Profitability of farming activities → Agricultural labour demand → Wage levels in the agricultural sector → Purchasing power of the population → Birth rate → Resident population → Consumption of food, goods, and services → Economic activity → Volume of surface water and groundwater → Availability of water resources → Agricultural demand for annual water allocations

**RF5i** **(Reinforcing)**	Agricultural demand for annual water allocations → Annual water allocations for agriculture → Crop Productivity → Profitability of farming activities → Agricultural labour demand → Wage levels in the agricultural sector → Purchasing power of the population → Birth rate → Resident population → Workforce → Irrigated crop area → Volume of surface water and groundwater → Availability of water resources → Environmental awareness → Agricultural demand for annual water allocations
**RF5j** **(Reinforcing)**	Agricultural demand for annual water allocations → Annual water allocations for agriculture → Crop Productivity → Profitability of farming activities→ Agricultural labour demand→ Wage levels in the agricultural sector → Purchasing power of the population → Birth rate → Resident population → Consumption of food, goods, and services → Economic activity → Volume of surface water and groundwater → Availability of water resources → Environmental awareness → Agricultural demand for annual water allocations
**RF5k** **(Reinforcing)**	Agricultural demand for annual water allocations → Annual water allocations for agriculture → Crop Productivity → Profitability of farming activities → Wage levels in the agricultural sector → Purchasing power of the population → Birth rate → Resident population → Workforce → Irrigated crop area → Agricultural labour demand → Employment rate → Consumption of food, goods, and services → Economic activity → Volume of surface water and groundwater → Availability of water resources → Agricultural demand for annual water allocations
**RF5l** **(Reinforcing)**	Agricultural demand for annual water allocations → Annual water allocations for agriculture → Crop Productivity → Profitability of farming activities → Wage levels in the agricultural sector → Purchasing power of the population → Birth rate → Resident population → Workforce → Irrigated crop area → Agricultural labour demand → Employment rate → Consumption of food, goods, and services → Economic activity → Volume of surface water and groundwater → Availability of water resources → Environmental awareness → Agricultural demand for annual water allocations
**Quality of Surface Water and Groundwater / Availability of Water Resources**	
**BF7** **(Balancing)**	Availability of water resources → Environmental awareness → Consumption of food, goods, and services → Economic activity → Quality of surface water and groundwater → Availability of water resources
**BF8a** **(Balancing)**	Agricultural demand for annual water allocations → Annual water allocations for agriculture → Irrigated crop area → Profitability of farming activities → Wage levels in the agricultural sector → Purchasing power of the population → Consumption of food, goods, and services → Economic activity → Quality of surface water and groundwater → Availability of water resources → Agricultural demand for annual water allocations
**BF8b** **(Balancing)**	Agricultural demand for annual water allocations → Annual water allocations for agriculture → Irrigated crop area → Profitability of farming activities → Agricultural labour demand → Employment rate → Consumption of food, goods, and services → Economic activity → Quality of surface water and groundwater → Availability of water resources → Agricultural demand for annual water allocations
**BF8c** **(Balancing)**	Agricultural demand for annual water allocations → Annual water allocations for agriculture → Irrigated crop area → Profitability of farming activities → Wage levels in the agricultural sector → Purchasing power of the population → Consumption of food, goods, and services → Economic activity → Quality of surface water and groundwater → Availability of water resources → Environmental awareness → Agricultural demand for annual water allocations

**BF8d** **(Balancing)**	Agricultural demand for annual water allocations → Annual water allocations for agriculture → Irrigated crop area → Profitability of farming activities → Agricultural labour demand → Employment rate → Consumption of food, goods, and services → Economic activity → Quality of surface water and groundwater → Availability of water resources → Environmental awareness → Agricultural demand for annual water allocations
**BF8e** **(Balancing)**	Agricultural demand for annual water allocations → Annual water allocations for agriculture → Irrigated crop area → Profitability of farming activities → Agricultural labour demand → Wage levels in the agricultural sector → Purchasing power of the population → Consumption of food, goods, and services → Economic activity → Quality of surface water and groundwater → Availability of water resources → Agricultural demand for annual water allocations
**BF8f** **(Balancing)**	Agricultural demand for annual water allocations → Annual water allocations for agriculture → Irrigated crop area → Profitability of farming activities → Wage levels in the agricultural sector → Immigration → Resident population → Consumption of food, goods, and services → Economic activity → Quality of surface water and groundwater → Availability of water resources → Agricultural demand for annual water allocations
**BF8g** **(Balancing)**	Agricultural demand for annual water allocations → Annual water allocations for agriculture → Irrigated crop area → Profitability of farming activities → Agricultural labour demand → Immigration → Resident population → Consumption of food, goods, and services → Economic activity → Quality of surface water and groundwater → Availability of water resources → Agricultural demand for annual water allocations
**BF8h** **(Balancing)**	Agricultural demand for annual water allocations → Annual water allocations for agriculture → Irrigated crop area → Profitability of farming activities → Agricultural labour demand → Immigration → Resident population → Consumption of food, goods, and services → Economic activity → Quality of surface water and groundwater → Availability of water resources → Environmental awareness → Agricultural demand for annual water allocations
**BF8i** **(Balancing)**	Agricultural demand for annual water allocations → Annual water allocations for agriculture → Irrigated crop area → Profitability of farming activities → Wage levels in the agricultural sector → Immigration → Resident population → Consumption of food, goods, and services → Economic activity → Quality of surface water and groundwater → Availability of water resources → Environmental awareness → Agricultural demand for annual water allocations
**BF8j** **(Balancing)**	Agricultural demand for annual water allocations → Annual water allocations for agriculture → Irrigated crop area → Profitability of farming activities → Agricultural labour demand → Wage levels in the agricultural sector → Immigration → Resident population → Consumption of food, goods, and services → Economic activity → Quality of surface water and groundwater → Availability of water resources → Agricultural demand for annual water allocations
**BF8k** **(Balancing)**	Agricultural demand for annual water allocations → Annual water allocations for agriculture → Irrigated crop area → Profitability of farming activities → Agricultural labour demand → Wage levels in the agricultural sector → Purchasing power of the population → Consumption of food, goods, and services → Economic activity → Quality of surface water and groundwater → Availability of water resources → Environmental awareness → Agricultural demand for annual water allocations

**BF8l (Balancing)**	Agricultural demand for annual water allocations → Annual water allocations for agriculture → Irrigated crop area → Profitability of farming activities → Agricultural labour demand → Wage levels in the agricultural sector → Immigration → Resident population → Consumption of food, goods, and services → Economic activity → Quality of surface water and groundwater → Availability of water resources → Environmental awareness → Agricultural demand for annual water allocations
**RF6a (Reinforcing)**	Agricultural demand for annual water allocations → Annual water allocations for agriculture → Irrigated crop area → Profitability of farming activities → Wage levels in the agricultural sector → Purchasing power of the population → Birth rate → Resident population → Consumption of food, goods, and services → Economic activity → Quality of surface water and groundwater → Availability of water resources → Agricultural demand for annual water allocations
**RF6b (Reinforcing)**	Agricultural demand for annual water allocations → Annual water allocations for agriculture → Irrigated crop area → Profitability of farming activities → Wage levels in the agricultural sector → Purchasing power of the population → Birth rate → Resident population → Consumption of food, goods, and services → Economic activity → Quality of surface water and groundwater → Availability of water resources → Environmental awareness → Agricultural demand for annual water allocations
**RF6c (Reinforcing)**	Agricultural demand for annual water allocations → Annual water allocations for agriculture → Irrigated crop area → Profitability of farming activities → Agricultural labour demand → Wage levels in the agricultural sector → Purchasing power of the population → Birth rate → Resident population → Consumption of food, goods, and services → Economic activity → Quality of surface water and groundwater → Availability of water resources → Agricultural demand for annual water allocations
**RF6d (Reinforcing)**	Agricultural demand for annual water allocations → Annual water allocations for agriculture → Irrigated crop area → Profitability of farming activities → Agricultural labour demand → Wage levels in the agricultural sector → Purchasing power of the population → Birth rate → Resident population → Consumption of food, goods, and services → Economic activity → Quality of surface water and groundwater → Availability of water resources → Environmental awareness → Agricultural demand for annual water allocations
**BF9a (Balancing)**	Agricultural demand for annual water allocations → Annual water allocations for agriculture → Irrigated crop area → Agricultural labour demand → Employment rate → Consumption of food, goods, and services → Economic activity → Quality of surface water and groundwater → Availability of water resources → Agricultural demand for annual water allocations
**BF9b (Balancing)**	Agricultural demand for annual water allocations → Annual water allocations for agriculture → Irrigated crop area → Agricultural labour demand → Wage levels in the agricultural sector → Purchasing power of the population → Consumption of food, goods, and services → Economic activity → Quality of surface water and groundwater → Availability of water resources → Agricultural demand for annual water allocations
**BF9c (Balancing)**	Agricultural demand for annual water allocations → Annual water allocations for agriculture → Irrigated crop area → Agricultural labour demand → Immigration → Resident population → Consumption of food, goods, and services → Economic activity → Quality of surface water and groundwater → Availability of water resources → Agricultural demand for annual water allocations

**BF9d** **(Balancing)**	Agricultural demand for annual water allocations → Annual water allocations for agriculture → Irrigated crop area → Agricultural labour demand → Employment rate → Consumption of food, goods, and services → Economic activity → Quality of surface water and groundwater → Availability of water resources → Environmental awareness → Agricultural demand for annual water allocations
**BF9e** **(Balancing)**	Agricultural demand for annual water allocations → Annual water allocations for agriculture → Irrigated crop area → Agricultural labour demand → Wage levels in the agricultural sector → Immigration → Resident population → Consumption of food, goods, and services → Economic activity → Quality of surface water and groundwater → Availability of water resources → Agricultural demand for annual water allocations
**BF9f** **(Balancing)**	Agricultural demand for annual water allocations → Annual water allocations for agriculture → Irrigated crop area → Agricultural labour demand → Wage levels in the agricultural sector → Purchasing power of the population → Consumption of food, goods, and services → Economic activity → Quality of surface water and groundwater → Availability of water resources → Environmental awareness → Agricultural demand for annual water allocations
**BF9g** **(Balancing)**	Agricultural demand for annual water allocations → Annual water allocations for agriculture → Irrigated crop area → Agricultural labour demand → Immigration → Resident population → Consumption of food, goods, and services → Economic activity → Quality of surface water and groundwater → Availability of water resources → Environmental awareness → Agricultural demand for annual water allocations
**BF9h** **(Balancing)**	Agricultural demand for annual water allocations → Annual water allocations for agriculture → Irrigated crop area → Agricultural labour demand → Wage levels in the agricultural sector → Immigration → Resident population → Consumption of food, goods, and services → Economic activity → Quality of surface water and groundwater → Availability of water resources → Environmental awareness → Agricultural demand for annual water allocations
**BF9i** **(Balancing)**	Agricultural demand for annual water allocations → Annual water allocations for agriculture → Irrigated crop area → Agricultural labour demand → Immigration → Resident population → Workforce → Profitability of farming activities → Wage levels in the agricultural sector → Purchasing power of the population → Consumption of food, goods, and services → Economic activity → Quality of surface water and groundwater → Availability of water resources → Agricultural demand for annual water allocations
**BF9j** **(Balancing)**	Agricultural demand for annual water allocations → Annual water allocations for agriculture → Irrigated crop area → Agricultural labour demand → Workforce → Profitability of farming activities → Wage levels in the agricultural sector → Purchasing power of the population → Birth rate → Resident population → Consumption of food, goods, and services → Economic activity → Quality of surface water and groundwater → Availability of water resources → Agricultural demand for annual water allocations
**BF9k** **(Balancing)**	Agricultural demand for annual water allocations → Annual water allocations for agriculture → Irrigated crop area → Agricultural labour demand → Workforce → Profitability of farming activities → Wage levels in the agricultural sector → Purchasing power of the population → Birth rate → Resident population → Consumption of food, goods, and services → Economic activity → Quality of surface water and groundwater → Availability of water resources → Environmental awareness → Agricultural demand for annual water allocations

**BF9l (Balancing)**	Agricultural demand for annual water allocations → Annual water allocations for agriculture → Irrigated crop area → Agricultural labour demand → Immigration → Resident population → Workforce → Profitability of farming activities → Wage levels in the agricultural sector → Purchasing power of the population → Consumption of food, goods, and services → Economic activity → Quality of surface water and groundwater → Availability of water resources → Environmental awareness → Agricultural demand for annual water allocations
**RF7a (Reinforcing)**	Agricultural demand for annual water allocations → Annual water allocations for agriculture → Irrigated crop area → Agricultural labour demand → Workforce → Profitability of farming activities → Wage levels in the agricultural sector → Purchasing power of the population → Consumption of food, goods, and services → Economic activity → Quality of surface water and groundwater → Availability of water resources → Agricultural demand for annual water allocations
**RF7b (Reinforcing)**	Agricultural demand for annual water allocations → Annual water allocations for agriculture → Irrigated crop area → Agricultural labour demand → Wage levels in the agricultural sector → Purchasing power of the population → Birth rate → Resident population → Consumption of food, goods, and services → Economic activity → Quality of surface water and groundwater → Availability of water resources → Agricultural demand for annual water allocations
**RF7c (Reinforcing)**	Agricultural demand for annual water allocations → Annual water allocations for agriculture → Irrigated crop area → Agricultural labour demand → Workforce → Profitability of farming activities → Wage levels in the agricultural sector → Immigration → Resident population → Consumption of food, goods, and services → Economic activity → Quality of surface water and groundwater → Availability of water resources → Agricultural demand for annual water allocations
**RF7d (Reinforcing)**	Agricultural demand for annual water allocations → Annual water allocations for agriculture → Irrigated crop area → Agricultural labour demand → Workforce → Profitability of farming activities → Wage levels in the agricultural sector → Purchasing power of the population → Consumption of food, goods, and services → Economic activity → Quality of surface water and groundwater → Availability of water resources → Environmental awareness → Agricultural demand for annual water allocations
**RF7e (Reinforcing)**	Agricultural demand for annual water allocations → Annual water allocations for agriculture → Irrigated crop area → Agricultural labour demand → Wage levels in the agricultural sector → Purchasing power of the population → Birth rate → Resident population → Consumption of food, goods, and services → Economic activity → Quality of surface water and groundwater → Availability of water resources → Environmental awareness → Agricultural demand for annual water allocations
**RF7f (Reinforcing)**	Agricultural demand for annual water allocations → Annual water allocations for agriculture → Irrigated crop area → Agricultural labour demand → Workforce → Profitability of farming activities → Wage levels in the agricultural sector → Immigration → Resident population → Consumption of food, goods, and services → Economic activity → Quality of surface water and groundwater → Availability of water resources → Environmental awareness → Agricultural demand for annual water allocations
**BF10a (Balancing)**	Agricultural demand for annual water allocations → Annual water allocations for agriculture → Crop Productivity → Profitability of farming activities → Wage levels in the agricultural sector → Purchasing power of the population → Consumption of food, goods, and services → Economic activity → Quality of surface water and groundwater → Availability of water resources → Agricultural demand for annual water allocations

**BF10b (Balancing)**	Agricultural demand for annual water allocations → Annual water allocations for agriculture → Crop Productivity → Profitability of farming activities → Agricultural labour demand → Employment rate → Consumption of food, goods, and services → Economic activity → Quality of surface water and groundwater → Availability of water resources → Agricultural demand for annual water allocations
**BF10c (Balancing)**	Agricultural demand for annual water allocations → Annual water allocations for agriculture → Crop Productivity → Profitability of farming activities → Wage levels in the agricultural sector → Purchasing power of the population → Consumption of food, goods, and services → Economic activity → Quality of surface water and groundwater → Availability of water resources → Environmental awareness → Agricultural demand for annual water allocations
**BF10d (Balancing)**	Agricultural demand for annual water allocations → Annual water allocations for agriculture → Crop Productivity → Profitability of farming activities → Agricultural labour demand → Immigration → Resident population → Consumption of food, goods, and services → Economic activity → Quality of surface water and groundwater → Availability of water resources → Agricultural demand for annual water allocations
**BF10e (Balancing)**	Agricultural demand for annual water allocations → Annual water allocations for agriculture → Crop Productivity → Profitability of farming activities → Agricultural labour demand → Wage levels in the agricultural sector → Purchasing power of the population → Consumption of food, goods, and services → Economic activity → Quality of surface water and groundwater → Availability of water resources → Agricultural demand for annual water allocations
**BF10f (Balancing)**	Agricultural demand for annual water allocations → Annual water allocations for agriculture → Crop Productivity → Profitability of farming activities → Agricultural labour demand → Employment rate → Consumption of food, goods, and services → Economic activity → Quality of surface water and groundwater → Availability of water resources → Environmental awareness → Agricultural demand for annual water allocations
**BF10g (Balancing)**	Agricultural demand for annual water allocations → Annual water allocations for agriculture → Crop Productivity → Profitability of farming activities → Wage levels in the agricultural sector → Immigration → Resident population → Consumption of food, goods, and services → Economic activity → Quality of surface water and groundwater → Availability of water resources → Agricultural demand for annual water allocations
**BF10h (Balancing)**	Agricultural demand for annual water allocations → Annual water allocations for agriculture → Crop Productivity → Profitability of farming activities → Agricultural labour demand → Wage levels in the agricultural sector → Immigration → Resident population → Consumption of food, goods, and services → Economic activity → Quality of surface water and groundwater → Availability of water resources → Agricultural demand for annual water allocations
**BF10i (Balancing)**	Agricultural demand for annual water allocations → Annual water allocations for agriculture → Crop Productivity → Profitability of farming activities → Agricultural labour demand → Wage levels in the agricultural sector → Purchasing power of the population → Consumption of food, goods, and services → Economic activity → Quality of surface water and groundwater → Availability of water resources → Environmental awareness → Agricultural demand for annual water allocations

**BF10j** **(Balancing)**	Agricultural demand for annual water allocations → Annual water allocations for agriculture → Crop Productivity → Profitability of farming activities → Agricultural labour demand → Immigration → Resident population → Consumption of food, goods, and services → Economic activity → Quality of surface water and groundwater → Availability of water resources → Environmental awareness → Agricultural demand for annual water allocations
**BF10k** **(Balancing)**	Agricultural demand for annual water allocations → Annual water allocations for agriculture → Crop Productivity → Profitability of farming activities → Wage levels in the agricultural sector → Immigration → Resident population → Consumption of food, goods, and services → Economic activity → Quality of surface water and groundwater → Availability of water resources → Environmental awareness → Agricultural demand for annual water allocations
**BF10l** **(Balancing)**	Agricultural demand for annual water allocations → Annual water allocations for agriculture → Crop Productivity → Profitability of farming activities → Agricultural labour demand → Wage levels in the agricultural sector → Immigration → Resident population → Consumption of food, goods, and services → Economic activity → Quality of surface water and groundwater → Availability of water resources → Environmental awareness → Agricultural demand for annual water allocations
**BF10m** **(Balancing)**	Agricultural demand for annual water allocations → Annual water allocations for agriculture → Crop Productivity → Profitability of farming activities → Wage levels in the agricultural sector → Immigration → Resident population → Workforce → Irrigated crop area → Agricultural labour demand → Employment rate → Consumption of food, goods, and services → Economic activity → Quality of surface water and groundwater → Availability of water resources → Agricultural demand for annual water allocations
**BF10n** **(Balancing)**	Agricultural demand for annual water allocations → Annual water allocations for agriculture → Crop Productivity → Profitability of farming activities → Wage levels in the agricultural sector → Immigration → Resident population → Workforce → Irrigated crop area → Agricultural labour demand → Employment rate → Consumption of food, goods, and services → Economic activity → Quality of surface water and groundwater → Availability of water resources → Environmental awareness → Agricultural demand for annual water allocations
**RF8a** **(Reinforcing)**	Agricultural demand for annual water allocations → Annual water allocations for agriculture → Crop Productivity → Profitability of farming activities → Wage levels in the agricultural sector → Purchasing power of the population → Birth rate → Resident population → Consumption of food, goods, and services → Economic activity → Quality of surface water and groundwater → Availability of water resources → Agricultural demand for annual water allocations
**RF8b** **(Reinforcing)**	Agricultural demand for annual water allocations → Annual water allocations for agriculture → Crop Productivity → Profitability of farming activities → Wage levels in the agricultural sector → Purchasing power of the population → Birth rate → Resident population → Consumption of food, goods, and services → Economic activity → Quality of surface water and groundwater → Availability of water resources → Environmental awareness → Agricultural demand for annual water allocations

**RF8c** **(Reinforcing)**	Agricultural demand for annual water allocations → Annual water allocations for agriculture → Crop Productivity → Profitability of farming activities → Agricultural labour demand → Wage levels in the agricultural sector → Purchasing power of the population → Birth rate → Resident population → Consumption of food, goods, and services → Economic activity → Quality of surface water and groundwater → Availability of water resources → Agricultural demand for annual water allocations
**RF8d** **(Reinforcing)**	Agricultural demand for annual water allocations → Annual water allocations for agriculture → Crop Productivity → Profitability of farming activities → Agricultural labour demand → Wage levels in the agricultural sector → Purchasing power of the population → Birth rate → Resident population → Consumption of food, goods, and services → Economic activity → Quality of surface water and groundwater → Availability of water resources → Environmental awareness → Agricultural demand for annual water allocations
**RF8e** **(Reinforcing)**	Agricultural demand for annual water allocations → Annual water allocations for agriculture → Crop Productivity → Profitability of farming activities → Wage levels in the agricultural sector → Purchasing power of the population → Birth rate → Resident population → Workforce → Irrigated crop area → Agricultural labour demand → Employment rate → Consumption of food, goods, and services → Economic activity → Quality of surface water and groundwater → Availability of water resources → Agricultural demand for annual water allocations
**RF8f** **(Reinforcing)**	Agricultural demand for annual water allocations → Annual water allocations for agriculture → Crop Productivity → Profitability of farming activities → Wage levels in the agricultural sector → Purchasing power of the population → Birth rate → Resident population → Workforce → Irrigated crop area → Agricultural labour demand → Employment rate → Consumption of food, goods, and services → Economic activity → Quality of surface water and groundwater → Availability of water resources → Environmental awareness → Agricultural demand for annual water allocations
	**Agricultural Labour Demand / Resident Population / Workforce**
**RF9a** **(Reinforcing)**	Resident population → Workforce → Irrigated crop area → Agricultural labour demand → Immigration → Resident population
**RF9b** **(Reinforcing)**	Resident population → Workforce → Irrigated crop area → Agricultural labour demand → Wage levels in the agricultural sector → Immigration → Resident population
**RF9c** **(Reinforcing)**	Resident population → Workforce → Irrigated crop area → Profitability of farming activities → Agricultural labour demand → Immigration → Resident population
**RF9d** **(Reinforcing)**	Resident population → Workforce → Irrigated crop area → Profitability of farming activities → Wage levels in the agricultural sector → Immigration →Resident population
**RF9e** **(Reinforcing)**	Resident population → Workforce → Irrigated crop area → Profitability of farming activities → Agricultural labour demand → Wage levels in the agricultural sector → Immigration → Resident population
**BF11** **(Balancing)**	Agricultural labour demand → Workforce → Profitability of farming activities → Agricultural labour demand
**BF12a** **(Balancing)**	Agricultural labour demand → Workforce → Irrigated crop area → Agricultural labour demand
**BF12b** **(Balancing)**	Agricultural labour demand → Workforce → Irrigated crop area → Profitability of farming activities → Agricultural labour demand

**BF12c (Balancing)**	Resident population → Workforce → Irrigated crop area → Agricultural labour demand → Wage levels in the agricultural sector → Purchasing power of the population → Birth rate → Resident population
**BF12d (Balancing)**	Resident population → Workforce → Irrigated crop area → Profitability of farming activities → Wage levels in the agricultural sector → Purchasing power of the population → Birth rate → Resident population
**BF12e (Balancing)**	Resident population → Workforce → Irrigated crop area → Profitability of farming activities → Agricultural labour demand → Wage levels in the agricultural sector → Purchasing power of the population → Birth rate → Resident population
**RF10a (Reinforcing)**	Resident population → Workforce → Profitability of farming activities → Wage levels in the agricultural sector → Immigration → Resident population
**RF10b (Reinforcing)**	Resident population → Workforce → Profitability of farming activities → Agricultural labour demand → Immigration → Resident population
**RF10c (Reinforcing)**	Resident population → Workforce → Profitability of farming activities → Agricultural labour demand → Wage levels in the agricultural sector → Immigration → Resident population
**BF13a (Balancing)**	Resident population → Workforce → Profitability of farming activities → Wage levels in the agricultural sector → Purchasing power of the population → Birth rate → Resident population
**BF13b (Balancing)**	Resident population → Workforce → Profitability of farming activities → Agricultural labour demand → Wage levels in the agricultural sector → Purchasing power of the population → Birth rate → Resident population
**RF11a (Reinforcing)**	Agricultural demand for annual water allocations → Annual water allocations for agriculture → Irrigated crop area → Agricultural labour demand → Immigration → Resident population → Workforce → Profitability of farming activities → Cultivation of water-intensive crops → Agricultural demand for annual water allocations
**RF11b (Reinforcing)**	Agricultural demand for annual water allocations → Annual water allocations for agriculture → Irrigated crop area → Agricultural labour demand → Wage levels in the agricultural sector → Immigration → Resident population → Workforce → Profitability of farming activities → Cultivation of water-intensive crops → Agricultural demand for annual water allocations
**BF14a (Balancing)**	Agricultural demand for annual water allocations → Annual water allocations for agriculture → Irrigated crop area → Agricultural labour demand → Workforce → Profitability of farming activities → Cultivation of water-intensive crops → Agricultural demand for annual water allocations
**BF14b (Balancing)**	Agricultural demand for annual water allocations → Annual water allocations for agriculture → Irrigated crop area → Agricultural labour demand → Wage levels in the agricultural sector → Purchasing power of the population → Birth rate → Resident population → Workforce → Profitability of farming activities → Cultivation of water-intensive crops → Agricultural demand for annual water allocations
**RF12a (Reinforcing)**	Agricultural demand for annual water allocations → Annual water allocations for agriculture → Crop Productivity → Profitability of farming activities → Agricultural labour demand → Immigration → Resident population → Workforce → Irrigated crop area → Agricultural demand for annual water allocations
**RF12b (Reinforcing)**	Agricultural demand for annual water allocations → Annual water allocations for agriculture → Crop Productivity → Profitability of farming activities → Wage levels in the agricultural sector → Immigration → Resident population → Workforce → Irrigated crop area → Agricultural demand for annual water allocations

**RF12c** **(Reinforcing)**	Agricultural demand for annual water allocations → Annual water allocations for agriculture → Crop Productivity → Profitability of farming activities → Agricultural labour demand → Wage levels in the agricultural sector → Immigration → Resident population → Workforce → Irrigated crop area → Agricultural demand for annual water allocations
**BF15a** **(Balancing)**	Agricultural demand for annual water allocations → Annual water allocations for agriculture → Crop Productivity → Profitability of farming activities → Agricultural labour demand → Workforce → Irrigated crop area → Agricultural demand for annual water allocations
**BF15b** **(Balancing)**	Agricultural demand for annual water allocations → Annual water allocations for agriculture → Crop Productivity → Profitability of farming activities → Wage levels in the agricultural sector → Purchasing power of the population → Birth rate → Resident population → Workforce → Irrigated crop area → Agricultural demand for annual water allocations
**BF15c** **(Balancing)**	Agricultural demand for annual water allocations → Annual water allocations for agriculture → Crop Productivity → Profitability of farming activities → Agricultural labour demand → Wage levels in the agricultural sector → Purchasing power of the population → Birth rate → Resident population → Workforce → Irrigated crop area → Agricultural demand for annual water allocations

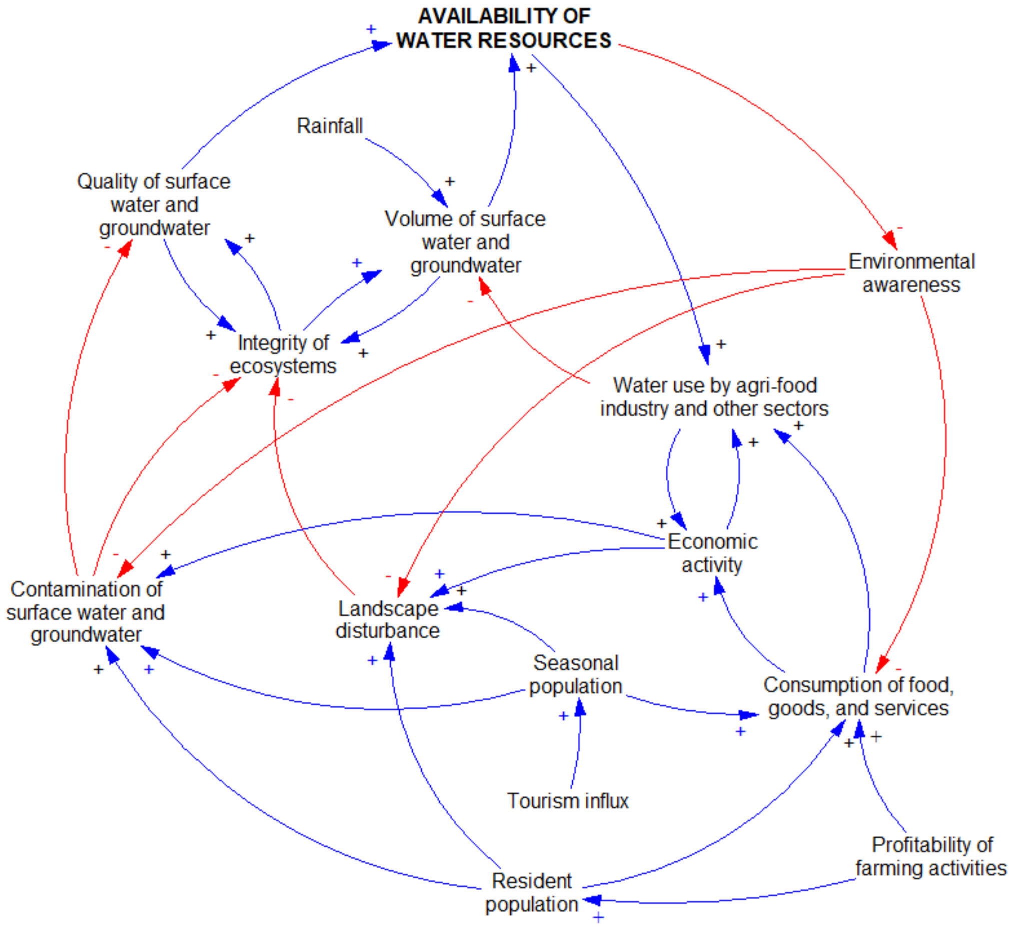

Figure SI-2.2. Causal loop diagram displaying the dynamics governing the interactions between population growth and ecosystems in Axarquia (**Figure 3** in manuscript).

Table SI-2.2. Description of the loops that govern the interactions between population growth and ecosystems in Axarquia (33 loops)

Nº	Loop
	**Economic Activity / Availability of Water Resources**
**RE1 (Reinforcing)**	Economic activity → Water use by agri-food industry and other sectors → Economic activity
**BE1 (Balancing)**	Availability of water resources → Water use by agri-food industry and other sectors → Volume of surface water and groundwater → Availability of water resources
**BE2 (Balancing)**	Availability of water resources → Water use by agri-food industry and other sectors → Volume of surface water and groundwater → Integrity of ecosystems → Quality of surface water and groundwater → Availability of water resources
**BE3 (Balancing)**	Availability of water resources → Water use by agri-food industry and other sectors → Economic activity → Contamination of surface water and groundwater → Integrity of ecosystems → Volume of surface water and groundwater → Availability of water resources
**BE4 (Balancing)**	Availability of water resources → Water use by agri-food industry and other sectors → Economic activity → Landscape disturbance → Integrity of ecosystems → Volume of surface water and groundwater → Availability of water resources

**BE5 (Balancing)**	Availability of water resources → Water use by agri-food industry and other sectors → Economic activity → Contamination of surface water and groundwater → Quality of surface water and groundwater → Integrity of ecosystems → Volume of surface water and groundwater → Availability of water resources
**BE6 (Balancing)**	Availability of water resources → Water use by agri-food industry and other sectors → Economic activity → Contamination of surface water and groundwater → Quality of surface water and groundwater → Availability of water resources
**BE7 (Balancing)**	Availability of water resources → Water use by agri-food industry and other sectors → Economic activity → Contamination of surface water and groundwater → Integrity of ecosystems → Quality of surface water and groundwater → Availability of water resources
**BE8 (Balancing)**	Availability of water resources → Water use by agri-food industry and other sectors → Economic activity → Landscape disturbance → Integrity of ecosystems → Quality of surface water and groundwater → Availability of water resources
	**Availability of Water Resources / Environmental Awareness**
**BE9 (Balancing)**	Availability of water resources → Environmental awareness → Contamination of surface water and groundwater → Quality of surface water and groundwater → Availability of water resources
**BE10 (Balancing)**	Availability of water resources → Environmental awareness → Landscape disturbance → Integrity of ecosystems → Quality of surface water and groundwater → Availability of water resources
**BE11 (Balancing)**	Availability of water resources → Environmental awareness → Contamination of surface water and groundwater → Integrity of ecosystems → Quality of surface water and groundwater → Availability of water resources
**BE12 (Balancing)**	Availability of water resources → Environmental awareness → Contamination of surface water and groundwater → Integrity of ecosystems → Volume of surface water and groundwater → Availability of water resources
**BE13 (Balancing)**	Availability of water resources → Environmental awareness → Landscape disturbance→ Integrity of ecosystems → Volume of surface water and groundwater → Availability of water resources
**BE14 (Balancing)**	Availability of water resources → Environmental awareness → Consumption of food, goods, and services → Water use by agri-food industry and other sectors → Volume of surface water and groundwater → Availability of water resources
**BE15 (Balancing)**	Availability of water resources → Environmental awareness → Contamination of surface water and groundwater → Quality of surface water and groundwater → Integrity of ecosystems → Volume of surface water and groundwater → Availability of water resources
**BE16 (Balancing)**	Availability of water resources → Environmental awareness → Consumption of food, goods, and services → Economic activity → Contamination of surface water and groundwater → Quality of surface water and groundwater → Availability of water resources
**BE17 (Balancing)**	Availability of water resources → Environmental awareness → Consumption of food, goods, and services → Economic activity → Water use by agri-food industry and other sectors → Volume of surface water and groundwater → Availability of water resources
**BE18 (Balancing)**	Availability of water resources → Environmental awareness → Consumption of food, goods, and services → Water use by agri-food industry and other sectors → Volume of surface water and groundwater → Integrity of ecosystems → Quality of surface water and groundwater → Availability of water resources
**BE19 (Balancing)**	Availability of water resources → Environmental awareness → Consumption of food, goods, and services → Economic activity → Landscape disturbance → Integrity of ecosystems → Quality of surface water and groundwater → Availability of water resources

**BE20** **(Balancing)**	Availability of water resources → Environmental awareness → Consumption of food, goods, and services → Water use by agri-food industry and other sectors → Economic activity → Contamination of surface water and groundwater → Quality of surface water and groundwater → Availability of water resources
**BE21** **(Balancing)**	Availability of water resources → Environmental awareness → Consumption of food, goods, and services → Economic activity → Landscape disturbance → Integrity of ecosystems → Volume of surface water and groundwater → Availability of water resources
**BE22** **(Balancing)**	Availability of water resources → Environmental awareness → Consumption of food, goods, and services → Economic activity → Contamination of surface water and groundwater → Integrity of ecosystems → Volume of surface water and groundwater → Availability of water resources
**BE23** **(Balancing)**	Availability of water resources → Environmental awareness → Consumption of food, goods, and services → Economic activity → Contamination of surface water and groundwater → Integrity of ecosystems → Quality of surface water and groundwater → Availability of water resources
**BE24** **(Balancing)**	Availability of water resources → Environmental awareness → Consumption of food, goods, and services → Water use by agri-food industry and other sectors → Economic activity → Landscape disturbance → Integrity of ecosystems → Volume of surface water and groundwater → Availability of water resources
**BE25** **(Balancing)**	Availability of water resources → Environmental awareness → Consumption of food, goods, and services → Water use by agri-food industry and other sectors → Economic activity → Landscape disturbance → Integrity of ecosystems → Quality of surface water and groundwater → Availability of water resources
**BE26** **(Balancing)**	Availability of water resources → Environmental awareness → Consumption of food, goods, and services → Water use by agri-food industry and other sectors → Economic activity → Contamination of surface water and groundwater → Integrity of ecosystems → Quality of surface water and groundwater → Availability of water resources
**BE27** **(Balancing)**	Availability of water resources → Environmental awareness → Consumption of food, goods, and services → Economic activity → Contamination of surface water and groundwater → Quality of surface water and groundwater → Integrity of ecosystems → Volume of surface water and groundwater → Availability of water resources
**BE28** **(Balancing)**	Availability of water resources → Environmental awareness → Consumption of food, goods, and services → Water use by agri-food industry and other sectors → Economic activity → Contamination of surface water and groundwater → Integrity of ecosystems → Volume of surface water and groundwater → Availability of water resources
**BE29** **(Balancing)**	Availability of water resources → Environmental awareness → Consumption of food, goods, and services → Economic activity → Water use by agri-food industry and other sectors → Volume of surface water and groundwater → Integrity of ecosystems → Quality of surface water and groundwater → Availability of water resources
**BE30** **(Balancing)**	Availability of water resources → Environmental awareness → Consumption of food, goods, and services → Water use by agri-food industry and other sectors → Economic activity → Contamination of surface water and groundwater → Quality of surface water and groundwater → Integrity of ecosystems → Volume of surface water and groundwater → Availability of water resources
	**Integrity of Ecosystems / Availability of Water Resources**
**RE2** **(Reinforcing)**	Integrity of ecosystems → Volume of surface water and groundwater → Integrity of ecosystems
**RE3** **(Reinforcing)**	Integrity of ecosystems → Quality of surface water and groundwater → Integrity of ecosystems
