# Supplementary material for: Systems Modeling of the Water-Energy-Food-Ecosystems Nexus: Insights from a Region Facing Structural Water Scarcity in Southern Spain
Source: Environ Manage. 2024 Sep 13;74(6):1045–62. doi: 10.1007/s00267-024-02037-6 (PMC11549115; doi:10.1007/s00267-024-02037-6)
Supplement: Supplementary file 3 — Appendix 3 [file 267_2024_2037_MOESM3_ESM.pdf]

Environmental Management

Supplementary Information

Appendix 3

Systems Modeling of the Water-Energy-Food-Ecosystems Nexus: Insights from a Region Facing Structural Water-Scarcity in Southern Spain

Antonio R. Hurtado[1,*], Enrique Mesa-Pérez[2], Julio Berbel[1]

[1] Water, Environmental and Agricultural Resources Economics (WEARE) Research Group, Department of Agricultural Economics, University of Cordoba, Campus Rabanales Building C5, 14014 Córdoba, Spain

[2] Departamento de Economía Financiera y Contabilidad, Universidad Loyola Andalucía, 41704 Dos Hermanas (Sevilla), Spain

*Corresponding author (es2rohuc@uco.es)

Water-Energy-Food (WEF) nexus

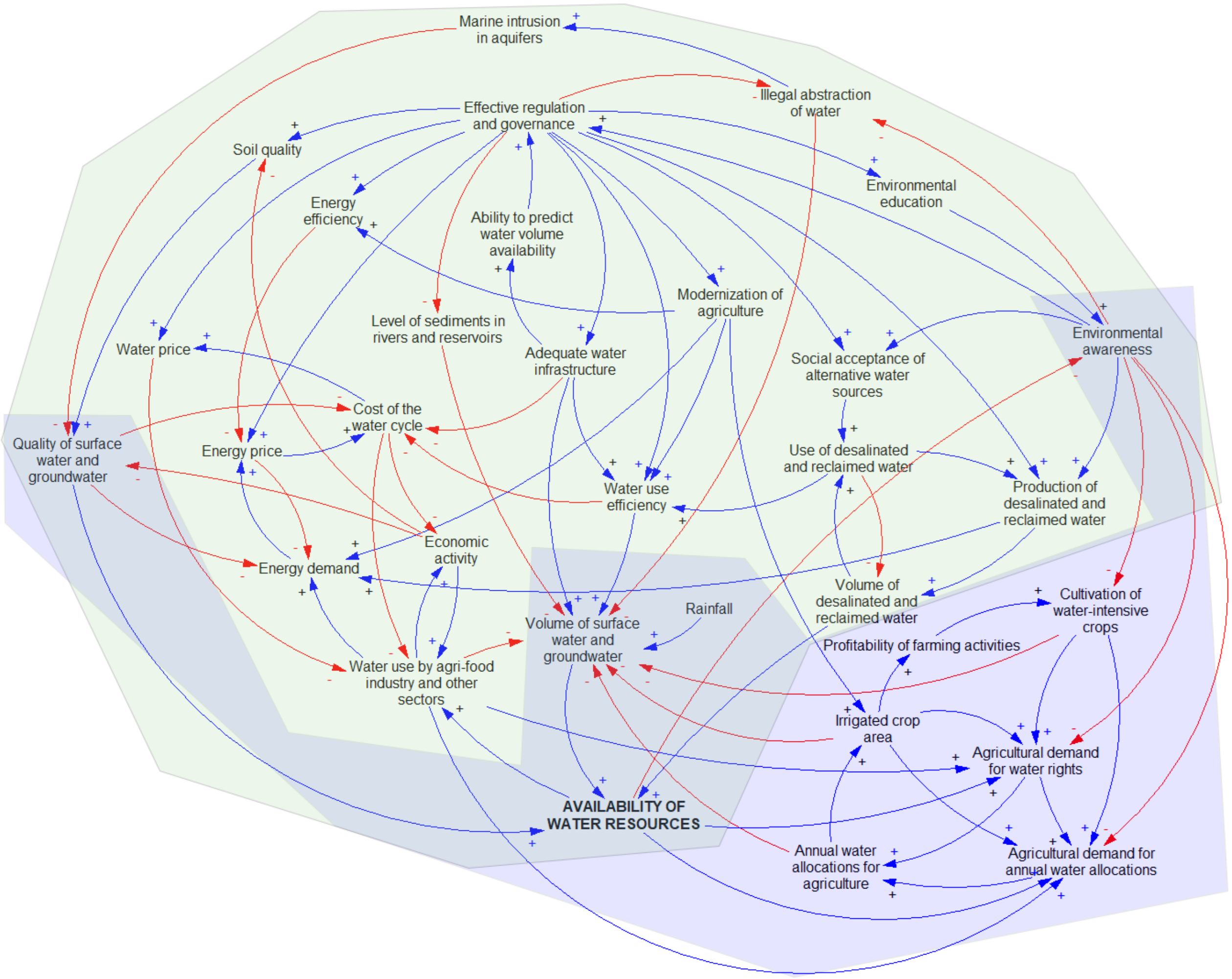

Figure SI-3. Causal loop diagram displaying the dynamics hypothesized to govern the WEF nexus in Axarquía. Blue shade: interactions between agriculture and the economy (**Figure 2** in manuscript); green shade: interactions in the water-energy system (**Figure 4** in manuscript).

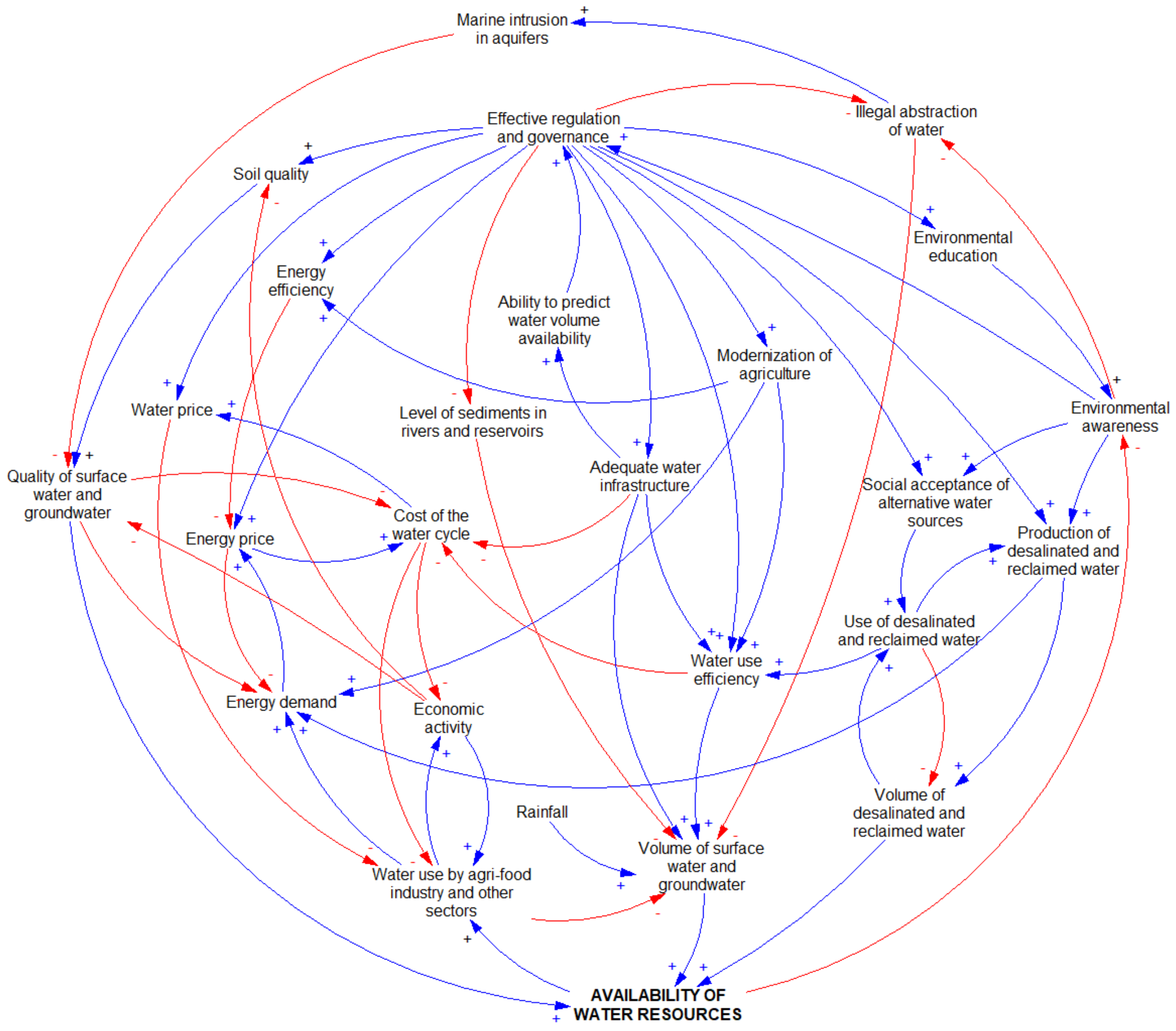

Figure SI-3.1. Causal loop diagram displaying the dynamics governing the water-energy system in Axarquia (**Figure 4** in manuscript).

Table SI-3.1. Description of the loops that govern the water-energy system in Axarquia (213 loops)

Nº	Loop
	**Effective Regulation and Governance**
**RW1 (Reinforcing)**	Effective regulation and governance → Environmental education → Environmental awareness → Effective regulation and governance
**RW2 (Reinforcing)**	Effective regulation and governance → Adequate water infrastructure → Ability to predict water volume availability → Effective regulation and governance
**BW1a (Balancing)**	Availability of water resources → Environmental awareness → Effective regulation and governance → Adequate water infrastructure → Volume of surface water and groundwater → Availability of water resources
**BW1b (Balancing)**	Availability of water resources → Environmental awareness → Effective regulation and governance → Adequate water infrastructure → Water use efficiency → Volume of surface water and groundwater → Availability of water resources

**BW2a** **(Balancing)**	Availability of water resources → Environmental awareness → Effective regulation and governance → Illegal abstraction of water → Marine intrusion in aquifers → Quality of surface water and groundwater → Availability of water resources
**BW2b** **(Balancing)**	Availability of water resources → Environmental awareness → Effective regulation and governance → Illegal abstraction of water → Volume of surface water and groundwater → Availability of water resources
**BW3** **(Balancing)**	Availability of water resources → Environmental awareness → Effective regulation and governance → Modernization of agriculture → Water use efficiency → Volume of surface water and groundwater → Availability of water resources
**BW4** **(Balancing)**	Availability of water resources → Environmental awareness → Effective regulation and governance → Level of sediments in rivers and reservoirs → Volume of surface water and groundwater → Availability of water resources
**BW5** **(Balancing)**	Availability of water resources → Environmental awareness → Effective regulation and governance → Water use efficiency → Volume of surface water and groundwater → Availability of water resources
**BW6** **(Balancing)**	Availability of water resources → Environmental awareness → Effective regulation and governance → Soil quality → Quality of surface water and groundwater → Availability of water resources
**BW7a** **(Balancing)**	Availability of water resources → Environmental awareness → Effective regulation and governance → Water price → Water use by agri-food industry and other sectors → Volume of surface water and groundwater → Availability of water resources
**BW7b** **(Balancing)**	Availability of water resources → Environmental awareness → Effective regulation and governance → Water price → Water use by agri-food industry and other sectors → Economic activity → Quality of surface water and groundwater → Availability of water resources
**BW7c** **(Balancing)**	Availability of water resources → Environmental awareness → Effective regulation and governance → Water price → Water use by agri-food industry and other sectors → Economic activity → Soil quality → Quality of surface water and groundwater → Availability of water resources
	**Effective Regulation and Governance / Cost of the Water Cycle**
**RW3a** **(Reinforcing)**	Availability of water resources → Environmental awareness → Effective regulation and governance → Adequate water infrastructure → Cost of the water cycle → Economic activity → Quality of surface water and groundwater → Availability of water resources
**RW3b** **(Reinforcing)**	Availability of water resources → Environmental awareness → Effective regulation and governance → Adequate water infrastructure → Cost of the water cycle → Water use by agri-food industry and other sectors → Volume of surface water and groundwater → Availability of water resources
**RW3c** **(Reinforcing)**	Availability of water resources → Environmental awareness → Effective regulation and governance → Adequate water infrastructure → Cost of the water cycle → Water use by agri-food industry and other sectors → Economic activity → Quality of surface water and groundwater → Availability of water resources
**RW3d** **(Reinforcing)**	Availability of water resources → Environmental awareness → Effective regulation and governance → Adequate water infrastructure → Cost of the water cycle → Water price → Water use by agri-food industry and other sectors → Volume of surface water and groundwater → Availability of water resources
**RW3e** **(Reinforcing)**	Availability of water resources → Environmental awareness → Effective regulation and governance → Adequate water infrastructure → Cost of the water cycle → Economic activity → Water use by agri-food industry and other sectors → Volume of surface water and groundwater → Availability of water resources
**RW3f** **(Reinforcing)**	Availability of water resources → Environmental awareness → Effective regulation and governance → Adequate water infrastructure → Cost of the water cycle → Economic

	activity → Soil quality → Quality of surface water and groundwater → Availability of water resources
**RW3g (Reinforcing)**	Availability of water resources → Environmental awareness → Effective regulation and governance → Adequate water infrastructure → Cost of the water cycle → Water price → Water use by agri-food industry and other sectors → Economic activity → Quality of surface water and groundwater → Availability of water resources
**RW3h (Reinforcing)**	Availability of water resources → Environmental awareness → Effective regulation and governance → Adequate water infrastructure → Cost of the water cycle → Water use by agri-food industry and other sectors → Economic activity → Soil quality → Quality of surface water and groundwater → Availability of water resources
**RW3i (Reinforcing)**	Availability of water resources → Environmental awareness → Effective regulation and governance → Adequate water infrastructure → Cost of the water cycle → Water price → Water use by agri-food industry and other sectors → Economic activity → Soil quality → Quality of surface water and groundwater → Availability of water resources
**RW4a (Reinforcing)**	Availability of water resources → Environmental awareness → Effective regulation and governance → Adequate water infrastructure → Water use efficiency → Cost of the water cycle → Water use by agri-food industry and other sectors → Volume of surface water and groundwater → Availability of water resources
**RW4b (Reinforcing)**	Availability of water resources → Environmental awareness → Effective regulation and governance → Adequate water infrastructure → Water use efficiency → Cost of the water cycle → Economic activity → Quality of surface water and groundwater → Availability of water resources
**RW4c (Reinforcing)**	Availability of water resources → Environmental awareness → Effective regulation and governance → Adequate water infrastructure → Water use efficiency → Cost of the water cycle → Economic activity → Water use by agri-food industry and other sectors → Volume of surface water and groundwater → Availability of water resources
**RW4d (Reinforcing)**	Availability of water resources → Environmental awareness → Effective regulation and governance → Adequate water infrastructure → Water use efficiency → Cost of the water cycle → Water price → Water use by agri-food industry and other sectors → Volume of surface water and groundwater → Availability of water resources
**RW4e (Reinforcing)**	Availability of water resources → Environmental awareness → Effective regulation and governance → Adequate water infrastructure → Water use efficiency → Cost of the water cycle → Water use by agri-food industry and other sectors → Economic activity → Quality of surface water and groundwater → Availability of water resources
**RW4f (Reinforcing)**	Availability of water resources → Environmental awareness → Effective regulation and governance → Adequate water infrastructure → Water use efficiency → Cost of the water cycle → Economic activity → Soil quality → Quality of surface water and groundwater → Availability of water resources
**RW4g (Reinforcing)**	Availability of water resources → Environmental awareness → Effective regulation and governance → Adequate water infrastructure → Water use efficiency → Cost of the water cycle → Water use by agri-food industry and other sectors → Economic activity → Soil quality → Quality of surface water and groundwater → Availability of water resources
**RW4h (Reinforcing)**	Availability of water resources → Environmental awareness → Effective regulation and governance → Adequate water infrastructure → Water use efficiency → Cost of the water cycle → Water price → Water use by agri-food industry and other sectors → Economic activity → Quality of surface water and groundwater → Availability of water resources
**RW4i (Reinforcing)**	Availability of water resources → Environmental awareness → Effective regulation and governance → Adequate water infrastructure → Water use efficiency → Cost of the water cycle → Water price → Water use by agri-food industry and other sectors → Economic activity → Soil quality → Quality of surface water and groundwater → Availability of water resources
**RW5a (Reinforcing)**	Availability of water resources → Environmental awareness → Effective regulation and governance → Illegal abstraction of water → Marine intrusion in aquifers → Quality of

	surface water and groundwater → Cost of the water cycle → Water use by agri-food industry and other sectors → Volume of surface water and groundwater → Availability of water resources
**RW5b** **(Reinforcing)**	Availability of water resources → Environmental awareness → Effective regulation and governance → Illegal abstraction of water → Marine intrusion in aquifers → Quality of surface water and groundwater → Cost of the water cycle → Economic activity → Water use by agri-food industry and other sectors → Volume of surface water and groundwater → Availability of water resources
**RW5c** **(Reinforcing)**	Availability of water resources → Environmental awareness → Effective regulation and governance → Illegal abstraction of water → Marine intrusion in aquifers → Quality of surface water and groundwater → Cost of the water cycle → Water price → Water use by agri-food industry and other sectors → Volume of surface water and groundwater → Availability of water resources
**RW5d** **(Reinforcing)**	Availability of water resources → Environmental awareness → Effective regulation and governance → Illegal abstraction of water → Marine intrusion in aquifers → Quality of surface water and groundwater → Energy demand → Energy price → Cost of the water cycle → Water use by agri-food industry and other sectors → Volume of surface water and groundwater → Availability of water resources
**RW5e** **(Reinforcing)**	Availability of water resources → Environmental awareness → Effective regulation and governance → Illegal abstraction of water → Marine intrusion in aquifers → Quality of surface water and groundwater → Energy demand → Energy price → Cost of the water cycle → Water price → Water use by agri-food industry and other sectors → Volume of surface water and groundwater → Availability of water resources
**RW5f** **(Reinforcing)**	Availability of water resources → Environmental awareness → Effective regulation and governance → Illegal abstraction of water → Marine intrusion in aquifers → Quality of surface water and groundwater → Energy demand → Energy price → Cost of the water cycle → Economic activity → Water use by agri-food industry and other sectors → Volume of surface water and groundwater → Availability of water resources
**RW6a** **(Reinforcing)**	Availability of water resources → Environmental awareness → Effective regulation and governance → Modernization of agriculture → Water use efficiency → Cost of the water cycle → Economic activity → Quality of surface water and groundwater → Availability of water resources
**RW6b** **(Reinforcing)**	Availability of water resources → Environmental awareness → Effective regulation and governance → Modernization of agriculture → Water use efficiency → Cost of the water cycle → Water use by agri-food industry and other sectors → Volume of surface water and groundwater → Availability of water resources
**RW6c** **(Reinforcing)**	Availability of water resources → Environmental awareness → Effective regulation and governance → Modernization of agriculture → Energy efficiency → Energy price → Cost of the water cycle → Water use by agri-food industry and other sectors → Volume of surface water and groundwater → Availability of water resources
**RW6d** **(Reinforcing)**	Availability of water resources → Environmental awareness → Effective regulation and governance → Modernization of agriculture → Water use efficiency → Cost of the water cycle → Economic activity → Soil quality → Quality of surface water and groundwater → Availability of water resources
**RW6e** **(Reinforcing)**	Availability of water resources → Environmental awareness → Effective regulation and governance → Modernization of agriculture → Water use efficiency → Cost of the water cycle → Water use by agri-food industry and other sectors → Economic activity → Quality of surface water and groundwater → Availability of water resources

**RW6f** **(Reinforcing)**	Availability of water resources → Environmental awareness → Effective regulation and governance → Modernization of agriculture → Water use efficiency → Cost of the water cycle → Water price → Water use by agri-food industry and other sectors → Volume of surface water and groundwater → Availability of water resources
**RW6g** **(Reinforcing)**	Availability of water resources → Environmental awareness → Effective regulation and governance → Modernization of agriculture → Water use efficiency → Cost of the water cycle → Economic activity → Water use by agri-food industry and other sectors → Volume of surface water and groundwater → Availability of water resources
**RW6h** **(Reinforcing)**	Availability of water resources → Environmental awareness → Effective regulation and governance → Modernization of agriculture → Energy efficiency → Energy price → Cost of the water cycle → Economic activity → Quality of surface water and groundwater → Availability of water resources
**RW6i** **(Reinforcing)**	Availability of water resources → Environmental awareness → Effective regulation and governance → Modernization of agriculture → Water use efficiency → Cost of the water cycle → Water use by agri-food industry and other sectors → Economic activity → Soil quality → Quality of surface water and groundwater → Availability of water resources
**RW6j** **(Reinforcing)**	Availability of water resources → Environmental awareness → Effective regulation and governance → Modernization of agriculture → Energy efficiency → Energy price → Cost of the water cycle → Water use by agri-food industry and other sectors → Economic activity → Quality of surface water and groundwater → Availability of water resources
**RW6k** **(Reinforcing)**	Availability of water resources → Environmental awareness → Effective regulation and governance → Modernization of agriculture → Energy efficiency → Energy price → Cost of the water cycle → Water price → Water use by agri-food industry and other sectors → Volume of surface water and groundwater → Availability of water resources
**RW6l** **(Reinforcing)**	Availability of water resources → Environmental awareness → Effective regulation and governance → Modernization of agriculture → Energy efficiency → Energy price → Cost of the water cycle → Economic activity → Soil quality → Quality of surface water and groundwater → Availability of water resources
**RW6m** **(Reinforcing)**	Availability of water resources → Environmental awareness → Effective regulation and governance → Modernization of agriculture → Water use efficiency → Cost of the water cycle → Water price → Water use by agri-food industry and other sectors → Economic activity → Quality of surface water and groundwater → Availability of water resources
**RW6n** **(Reinforcing)**	Availability of water resources → Environmental awareness → Effective regulation and governance → Modernization of agriculture → Energy efficiency → Energy price → Cost of the water cycle → Economic activity → Water use by agri-food industry and other sectors → Volume of surface water and groundwater → Availability of water resources
**RW6o** **(Reinforcing)**	Availability of water resources → Environmental awareness → Effective regulation and governance → Modernization of agriculture → Energy efficiency → Energy price → Cost of the water cycle → Water price → Water use by agri-food industry and other sectors → Economic activity → Quality of surface water and groundwater → Availability of water resources
**RW6p** **(Reinforcing)**	Availability of water resources → Environmental awareness → Effective regulation and governance → Modernization of agriculture → Energy efficiency → Energy price → Cost of the water cycle → Water use by agri-food industry and other sectors → Economic activity → Soil quality → Quality of surface water and groundwater → Availability of water resources
**RW6q** **(Reinforcing)**	Availability of water resources → Environmental awareness → Effective regulation and governance → Modernization of agriculture → Water use efficiency → Cost of the water cycle → Water price → Water use by agri-food industry and other sectors → Economic activity → Soil quality → Quality of surface water and groundwater → Availability of water resources

**RW6r** **(Reinforcing)**	Availability of water resources → Environmental awareness → Effective regulation and governance → Modernization of agriculture → Energy efficiency → Energy price → Cost of the water cycle → Water price → Water use by agri-food industry and other sectors → Economic activity → Soil quality → Quality of surface water and groundwater → Availability of water resources
**BW8a** **(Balancing)**	Availability of water resources → Environmental awareness → Effective regulation and governance → Modernization of agriculture → Energy demand → Energy price → Cost of the water cycle → Economic activity → Quality of surface water and groundwater → Availability of water resources
**BW8b** **(Balancing)**	Availability of water resources → Environmental awareness → Effective regulation and governance → Modernization of agriculture → Energy demand → Energy price → Cost of the water cycle → Water use by agri-food industry and other sectors → Volume of surface water and groundwater → Availability of water resources
**BW8c** **(Balancing)**	Availability of water resources → Environmental awareness → Effective regulation and governance → Modernization of agriculture → Energy demand → Energy price → Cost of the water cycle → Economic activity → Soil quality → Quality of surface water and groundwater → Availability of water resources
**BW8d** **(Balancing)**	Availability of water resources → Environmental awareness → Effective regulation and governance → Modernization of agriculture → Energy demand → Energy price → Cost of the water cycle → Economic activity → Water use by agri-food industry and other sectors → Volume of surface water and groundwater → Availability of water resources
**BW8e** **(Balancing)**	Availability of water resources → Environmental awareness → Effective regulation and governance → Modernization of agriculture → Energy demand → Energy price → Cost of the water cycle → Water price → Water use by agri-food industry and other sectors → Volume of surface water and groundwater → Availability of water resources
**BW8f** **(Balancing)**	Availability of water resources → Environmental awareness → Effective regulation and governance → Modernization of agriculture → Energy demand → Energy price → Cost of the water cycle → Water use by agri-food industry and other sectors → Economic activity → Quality of surface water and groundwater → Availability of water resources
**BW8g** **(Balancing)**	Availability of water resources → Environmental awareness → Effective regulation and governance → Modernization of agriculture → Energy demand → Energy price → Cost of the water cycle → Water use by agri-food industry and other sectors → Economic activity → Soil quality → Quality of surface water and groundwater → Availability of water resources
**BW8h** **(Balancing)**	Availability of water resources → Environmental awareness → Effective regulation and governance → Modernization of agriculture → Energy demand → Energy price → Cost of the water cycle → Water price → Water use by agri-food industry and other sectors → Economic activity → Quality of surface water and groundwater → Availability of water resources
**BW8i** **(Balancing)**	Availability of water resources → Environmental awareness → Effective regulation and governance → Modernization of agriculture → Energy demand → Energy price → Cost of the water cycle → Water price → Water use by agri-food industry and other sectors → Economic activity → Soil quality → Quality of surface water and groundwater → Availability of water resources
**RW7a** **(Reinforcing)**	Availability of water resources → Environmental awareness → Effective regulation and governance → Water use efficiency → Cost of the water cycle → Water use by agri-food industry and other sectors → Volume of surface water and groundwater → Availability of water resources
**RW7b** **(Reinforcing)**	Availability of water resources → Environmental awareness → Effective regulation and governance → Water use efficiency → Cost of the water cycle → Economic activity → Quality of surface water and groundwater → Availability of water resources

**RW7c** **(Reinforcing)**	Availability of water resources → Environmental awareness → Effective regulation and governance → Water use efficiency → Cost of the water cycle → Economic activity → Water use by agri-food industry and other sectors → Volume of surface water and groundwater → Availability of water resources
**RW7d** **(Reinforcing)**	Availability of water resources → Environmental awareness → Effective regulation and governance → Water use efficiency → Cost of the water cycle → Water price → Water use by agri-food industry and other sectors → Volume of surface water and groundwater → Availability of water resources
**RW7e** **(Reinforcing)**	Availability of water resources → Environmental awareness → Effective regulation and governance → Water use efficiency → Cost of the water cycle → Economic activity → Soil quality → Quality of surface water and groundwater → Availability of water resources
**RW7f** **(Reinforcing)**	Availability of water resources → Environmental awareness → Effective regulation and governance → Water use efficiency → Cost of the water cycle → Water use by agri-food industry and other sectors → Economic activity → Quality of surface water and groundwater → Availability of water resources
**RW7g** **(Reinforcing)**	Availability of water resources → Environmental awareness → Effective regulation and governance → Water use efficiency → Cost of the water cycle → Water use by agri-food industry and other sectors → Economic activity → Soil quality → Quality of surface water and groundwater → Availability of water resources
**RW7h** **(Reinforcing)**	Availability of water resources → Environmental awareness → Effective regulation and governance → Water use efficiency → Cost of the water cycle → Water price → Water use by agri-food industry and other sectors → Economic activity → Quality of surface water and groundwater → Availability of water resources
**RW7i** **(Reinforcing)**	Availability of water resources → Environmental awareness → Effective regulation and governance → Water use efficiency → Cost of the water cycle → Water price → Water use by agri-food industry and other sectors → Economic activity → Soil quality → Quality of surface water and groundwater → Availability of water resources
**RW8a** **(Reinforcing)**	Availability of water resources → Environmental awareness → Effective regulation and governance → Soil quality → Quality of surface water and groundwater → Cost of the water cycle → Water use by agri-food industry and other sectors → Volume of surface water and groundwater → Availability of water resources
**RW8b** **(Reinforcing)**	Availability of water resources → Environmental awareness → Effective regulation and governance → Soil quality → Quality of surface water and groundwater → Cost of the water cycle → Economic activity → Water use by agri-food industry and other sectors → Volume of surface water and groundwater → Availability of water resources
**RW8c** **(Reinforcing)**	Availability of water resources → Environmental awareness → Effective regulation and governance → Soil quality → Quality of surface water and groundwater → Cost of the water cycle → Water price → Water use by agri-food industry and other sectors → Volume of surface water and groundwater → Availability of water resources
**RW8d** **(Reinforcing)**	Availability of water resources → Environmental awareness → Effective regulation and governance → Soil quality → Quality of surface water and groundwater → Energy demand → Energy price → Cost of the water cycle → Water use by agri-food industry and other sectors → Volume of surface water and groundwater → Availability of water resources
**RW8e** **(Reinforcing)**	Availability of water resources → Environmental awareness → Effective regulation and governance → Soil quality → Quality of surface water and groundwater → Energy demand → Energy price → Cost of the water cycle → Economic activity → Water use by agri-food industry and other sectors → Volume of surface water and groundwater → Availability of water resources

**RW8f (Reinforcing)**	Availability of water resources → Environmental awareness → Effective regulation and governance → Soil quality → Quality of surface water and groundwater → Energy demand → Energy price → Cost of the water cycle → Water price → Water use by agri-food industry and other sectors → Volume of surface water and groundwater → Availability of water resources
**RW9a (Reinforcing)**	Availability of water resources → Environmental awareness → Effective regulation and governance → Water price → Water use by agri-food industry and other sectors → Energy demand → Energy price → Cost of the water cycle → Economic activity → Quality of surface water and groundwater → Availability of water resources
**RW9b (Reinforcing)**	Availability of water resources → Environmental awareness → Effective regulation and governance → Water price → Water use by agri-food industry and other sectors → Energy demand → Energy price → Cost of the water cycle → Economic activity → Soil quality → Quality of surface water and groundwater → Availability of water resources
**BW9a (Balancing)**	Availability of water resources → Environmental awareness → Effective regulation and governance → Energy price → Cost of the water cycle → Water use by agri-food industry and other sectors → Volume of surface water and groundwater → Availability of water resources
**BW9b (Balancing)**	Availability of water resources → Environmental awareness → Effective regulation and governance → Energy price → Cost of the water cycle → Economic activity → Quality of surface water and groundwater → Availability of water resources
**BW9c (Balancing)**	Availability of water resources → Environmental awareness → Effective regulation and governance → Energy price → Cost of the water cycle → Economic activity → Soil quality → Quality of surface water and groundwater → Availability of water resources
**BW9d (Balancing)**	Availability of water resources → Environmental awareness → Effective regulation and governance → Energy price → Cost of the water cycle → Water use by agri-food industry and other sectors → Economic activity → Quality of surface water and groundwater → Availability of water resources
**BW9e (Balancing)**	Availability of water resources → Environmental awareness → Effective regulation and governance → Energy price → Cost of the water cycle → Water price → Water use by agri-food industry and other sectors → Volume of surface water and groundwater → Availability of water resources
**BW9f (Balancing)**	Availability of water resources → Environmental awareness → Effective regulation and governance → Energy price → Cost of the water cycle → Economic activity → Water use by agri-food industry and other sectors → Volume of surface water and groundwater → Availability of water resources
**BW9g (Balancing)**	Availability of water resources → Environmental awareness → Effective regulation and governance → Energy price → Cost of the water cycle → Water use by agri-food industry and other sectors → Economic activity → Soil quality → Quality of surface water and groundwater → Availability of water resources
**BW9h (Balancing)**	Availability of water resources → Environmental awareness → Effective regulation and governance → Energy price → Cost of the water cycle → Water price → Water use by agri-food industry and other sectors → Economic activity → Quality of surface water and groundwater → Availability of water resources
**BW9i (Balancing)**	Availability of water resources → Environmental awareness → Effective regulation and governance → Energy price → Cost of the water cycle → Water price → Water use by agri-food industry and other sectors → Economic activity → Soil quality → Quality of surface water and groundwater → Availability of water resources
**RW10a (Reinforcing)**	Availability of water resources → Environmental awareness → Effective regulation and governance → Energy efficiency → Energy price → Cost of the water cycle → Water use by agri-food industry and other sectors → Volume of surface water and groundwater → Availability of water resources

**RW10b (Reinforcing)**	Availability of water resources → Environmental awareness → Effective regulation and governance → Energy efficiency → Energy price → Cost of the water cycle → Economic activity → Quality of surface water and groundwater → Availability of water resources
**RW10c (Reinforcing)**	Availability of water resources → Environmental awareness → Effective regulation and governance → Energy efficiency → Energy price → Cost of the water cycle → Water price → Water use by agri-food industry and other sectors → Volume of surface water and groundwater → Availability of water resources
**RW10d (Reinforcing)**	Availability of water resources → Environmental awareness → Effective regulation and governance → Energy efficiency → Energy price → Cost of the water cycle → Water use by agri-food industry and other sectors → Economic activity → Quality of surface water and groundwater → Availability of water resources
**RW10e (Reinforcing)**	Availability of water resources → Environmental awareness → Effective regulation and governance → Energy efficiency → Energy price → Cost of the water cycle → Economic activity → Soil quality → Quality of surface water and groundwater → Availability of water resources
**RW10f (Reinforcing)**	Availability of water resources → Environmental awareness → Effective regulation and governance → Energy efficiency → Energy price → Cost of the water cycle → Economic activity → Water use by agri-food industry and other sectors → Volume of surface water and groundwater → Availability of water resources
**RW10g (Reinforcing)**	Availability of water resources → Environmental awareness → Effective regulation and governance → Energy efficiency → Energy price → Cost of the water cycle → Water use by agri-food industry and other sectors → Economic activity → Soil quality → Quality of surface water and groundwater → Availability of water resources
**RW10h (Reinforcing)**	Availability of water resources → Environmental awareness → Effective regulation and governance → Energy efficiency → Energy price → Cost of the water cycle → Water price → Water use by agri-food industry and other sectors → Economic activity → Quality of surface water and groundwater → Availability of water resources
**RW10i (Reinforcing)**	Availability of water resources → Environmental awareness → Effective regulation and governance → Energy efficiency → Energy price → Cost of the water cycle → Water price → Water use by agri-food industry and other sectors → Economic activity → Soil quality → Quality of surface water and groundwater → Availability of water resources
	**Cost of the Water Cycle**
**BW10 (Balancing)**	Cost of the water cycle → Water use by agri-food industry and other sectors → Energy demand → Energy price → Cost of the water cycle
**BW11 (Balancing)**	Cost of the water cycle → Water price → Water use by agri-food industry and other sectors → Energy demand → Energy price → Cost of the water cycle
**BW12 (Balancing)**	Cost of the water cycle → Economic activity → Water use by agri-food industry and other sectors → Energy demand → Energy price → Cost of the water cycle
**BW13a (Balancing)**	Quality of surface water and groundwater → Cost of the water cycle → Economic activity → Quality of surface water and groundwater
**BW13b (Balancing)**	Quality of surface water and groundwater → Cost of the water cycle → Economic activity → Soil quality → Quality of surface water and groundwater
**BW14a (Balancing)**	Quality of surface water and groundwater → Cost of the water cycle → Water use by agri-food industry and other sectors → Economic activity → Quality of surface water and groundwater
**BW14b (Balancing)**	Quality of surface water and groundwater → Cost of the water cycle → Water use by agri-food industry and other sectors → Economic activity → Soil quality → Quality of surface water and groundwater
**BW15a (Balancing)**	Quality of surface water and groundwater → Cost of the water cycle → Water price → Water use by agri-food industry and other sectors → Economic activity → Quality of surface water and groundwater

**BW15b** **(Balancing)**	Quality of surface water and groundwater → Cost of the water cycle → Water price → Water use by agri-food industry and other sectors → Economic activity → Soil quality → Quality of surface water and groundwater
**BW16a** **(Balancing)**	Quality of surface water and groundwater → Energy demand → Energy price → Cost of the water cycle → Economic activity → Quality of surface water and groundwater
**BW16b** **(Balancing)**	Quality of surface water and groundwater → Energy demand → Energy price → Cost of the water cycle → Economic activity → Soil quality → Quality of surface water and groundwater
**BW16c** **(Balancing)**	Quality of surface water and groundwater → Energy demand → Energy price → Cost of the water cycle → Water use by agri-food industry and other sectors → Economic activity → Quality of surface water and groundwater
**BW16d** **(Balancing)**	Quality of surface water and groundwater → Energy demand → Energy price → Cost of the water cycle → Water use by agri-food industry and other sectors → Economic activity → Soil quality → Quality of surface water and groundwater
**BW16e** **(Balancing)**	Quality of surface water and groundwater → Energy demand → Energy price → Cost of the water cycle → Water price → Water use by agri-food industry and other sectors → Economic activity → Quality of surface water and groundwater
**BW16f** **(Balancing)**	Quality of surface water and groundwater → Energy demand → Energy price → Cost of the water cycle → Water price → Water use by agri-food industry and other sectors → Economic activity → Soil quality → Quality of surface water and groundwater
**RW11a** **(Reinforcing)**	Availability of water resources → Water use by agri-food industry and other sectors → Energy demand → Energy price → Cost of the water cycle → Economic activity → Quality of surface water and groundwater → Availability of water resources
**RW11b** **(Reinforcing)**	Availability of water resources → Water use by agri-food industry and other sectors → Energy demand → Energy price → Cost of the water cycle → Economic activity → Soil quality → Quality of surface water and groundwater → Availability of water resources
**RW12a** **(Reinforcing)**	Availability of water resources → Environmental awareness → Illegal abstraction of water → Marine intrusion in aquifers → Quality of surface water and groundwater → Cost of the water cycle → Water use by agri-food industry and other sectors → Volume of surface water and groundwater → Availability of water resources
**RW12b** **(Reinforcing)**	Availability of water resources → Environmental awareness → Illegal abstraction of water → Marine intrusion in aquifers → Quality of surface water and groundwater → Cost of the water cycle → Economic activity → Water use by agri-food industry and other sectors → Volume of surface water and groundwater → Availability of water resources
**RW12c** **(Reinforcing)**	Availability of water resources → Environmental awareness → Illegal abstraction of water → Marine intrusion in aquifers → Quality of surface water and groundwater → Cost of the water cycle → Water price → Water use by agri-food industry and other sectors → Volume of surface water and groundwater → Availability of water resources
**RW12d** **(Reinforcing)**	Availability of water resources → Environmental awareness → Illegal abstraction of water → Marine intrusion in aquifers → Quality of surface water and groundwater → Energy demand → Energy price → Cost of the water cycle → Water use by agri-food industry and other sectors → Volume of surface water and groundwater → Availability of water resources
**RW12e** **(Reinforcing)**	Availability of water resources → Environmental awareness → Illegal abstraction of water → Marine intrusion in aquifers → Quality of surface water and groundwater → Energy demand → Energy price → Cost of the water cycle → Water price → Water use by agri-food industry and other sectors → Volume of surface water and groundwater → Availability of water resources

**RW12f** **(Reinforcing)**	Availability of water resources → Environmental awareness → Illegal abstraction of water → Marine intrusion in aquifers → Quality of surface water and groundwater → Energy demand → Energy price → Cost of the water cycle → Economic activity → Water use by agri-food industry and other sectors → Volume of surface water and groundwater → Availability of water resources
	**Water Desalination and Reclamation**
**RW13** **(Reinforcing)**	Production of desalinated and reclaimed water → Volume of desalinated and reclaimed water → Use of desalinated and reclaimed water → Production of desalinated and reclaimed water
**BW17** **(Balancing)**	Use of desalinated and reclaimed water → Volume of desalinated and reclaimed water → Use of desalinated and reclaimed water
**BW18** **(Balancing)**	Environmental awareness → Production of desalinated and reclaimed water → Volume of desalinated and reclaimed water → Availability of water resources → Environmental awareness
**BW19a** **(Balancing)**	Availability of water resources → Environmental awareness → Effective regulation and governance → Production of desalinated and reclaimed water → Volume of desalinated and reclaimed water → Availability of water resources
**BW19b** **(Balancing)**	Availability of water resources → Environmental awareness → Effective regulation and governance → Production of desalinated and reclaimed water → Volume of desalinated and reclaimed water → Use of desalinated and reclaimed water → Water use efficiency → Volume of surface water and groundwater → Availability of water resources
**BW19c** **(Balancing)**	Availability of water resources → Environmental awareness → Effective regulation and governance → Production of desalinated and reclaimed water → Energy demand → Energy price → Cost of the water cycle → Economic activity → Quality of surface water and groundwater → Availability of water resources
**BW19d** **(Balancing)**	Availability of water resources → Environmental awareness → Effective regulation and governance → Production of desalinated and reclaimed water → Energy demand → Energy price → Cost of the water cycle → Water use by agri-food industry and other sectors → Volume of surface water and groundwater → Availability of water resources
**BW19e** **(Balancing)**	Availability of water resources → Environmental awareness → Effective regulation and governance → Production of desalinated and reclaimed water → Energy demand → Energy price → Cost of the water cycle → Economic activity → Water use by agri-food industry and other sectors → Volume of surface water and groundwater → Availability of water resources
**BW19f** **(Balancing)**	Availability of water resources → Environmental awareness → Effective regulation and governance → Production of desalinated and reclaimed water → Energy demand → Energy price → Cost of the water cycle → Water use by agri-food industry and other sectors → Economic activity → Quality of surface water and groundwater → Availability of water resources
**BW19g** **(Balancing)**	Availability of water resources → Environmental awareness → Effective regulation and governance → Production of desalinated and reclaimed water → Energy demand → Energy price → Cost of the water cycle → Economic activity → Soil quality → Quality of surface water and groundwater → Availability of water resources
**BW19h** **(Balancing)**	Availability of water resources → Environmental awareness → Effective regulation and governance → Production of desalinated and reclaimed water → Energy demand → Energy price → Cost of the water cycle → Water price → Water use by agri-food industry and other sectors → Volume of surface water and groundwater → Availability of water resources
**BW19i** **(Balancing)**	Availability of water resources → Environmental awareness → Effective regulation and governance → Production of desalinated and reclaimed water → Energy demand → Energy price → Cost of the water cycle → Water price → Water use by agri-food industry and other sectors → Economic activity → Quality of surface water and groundwater → Availability of water resources

**BW19j (Balancing)**	Availability of water resources → Environmental awareness → Effective regulation and governance → Production of desalinated and reclaimed water → Energy demand → Energy price → Cost of the water cycle → Water use by agri-food industry and other sectors → Economic activity → Soil quality → Quality of surface water and groundwater → Availability of water resources
**BW19k (Balancing)**	Availability of water resources → Environmental awareness → Effective regulation and governance → Production of desalinated and reclaimed water → Energy demand → Energy price → Cost of the water cycle → Water price → Water use by agri-food industry and other sectors → Economic activity → Soil quality → Quality of surface water and groundwater → Availability of water resources
**RW14a (Reinforcing)**	Availability of water resources → Environmental awareness → Effective regulation and governance → Production of desalinated and reclaimed water → Volume of desalinated and reclaimed water → Use of desalinated and reclaimed water → Water use efficiency → Cost of the water cycle → Economic activity → Quality of surface water and groundwater → Availability of water resources
**RW14b (Reinforcing)**	Availability of water resources → Environmental awareness → Effective regulation and governance → Production of desalinated and reclaimed water → Volume of desalinated and reclaimed water →Use of desalinated and reclaimed water → Water use efficiency → Cost of the water cycle → Water use by agri-food industry and other sectors → Volume of surface water and groundwater → Availability of water resources
**RW14c (Reinforcing)**	Availability of water resources → Environmental awareness → Effective regulation and governance → Production of desalinated and reclaimed water → Volume of desalinated and reclaimed water → Use of desalinated and reclaimed water → Water use efficiency → Cost of the water cycle → Water use by agri-food industry and other sectors → Economic activity → Quality of surface water and groundwater → Availability of water resources
**RW14d (Reinforcing)**	Availability of water resources → Environmental awareness → Effective regulation and governance → Production of desalinated and reclaimed water → Volume of desalinated and reclaimed water → Use of desalinated and reclaimed water → Water use efficiency → Cost of the water cycle → Water price → Water use by agri-food industry and other sectors → Volume of surface water and groundwater → Availability of water resources
**RW14e (Reinforcing)**	Availability of water resources → Environmental awareness → Effective regulation and governance → Production of desalinated and reclaimed water → Volume of desalinated and reclaimed water → Use of desalinated and reclaimed water →Water use efficiency → Cost of the water cycle → Economic activity → Soil quality → Quality of surface water and groundwater → Availability of water resources
**RW14f (Reinforcing)**	Availability of water resources → Environmental awareness → Effective regulation and governance → Production of desalinated and reclaimed water → Volume of desalinated and reclaimed water → Use of desalinated and reclaimed water → Water use efficiency → Cost of the water cycle → Economic activity → Water use by agri-food industry and other sectors → Volume of surface water and groundwater → Availability of water resources
**RW14g (Reinforcing)**	Availability of water resources → Environmental awareness → Effective regulation and governance → Production of desalinated and reclaimed water → Volume of desalinated and reclaimed water → Use of desalinated and reclaimed water → Water use efficiency → Cost of the water cycle → Water price → Water use by agri-food industry and other sectors → Economic activity → Quality of surface water and groundwater → Availability of water resources

**RW14h (Reinforcing)**	Availability of water resources → Environmental awareness → Effective regulation and governance → Production of desalinated and reclaimed water → Volume of desalinated and reclaimed water → Use of desalinated and reclaimed water → Water use efficiency → Cost of the water cycle → Water use by agri-food industry and other sectors → Economic activity → Soil quality → Quality of surface water and groundwater → Availability of water resources
**RW14i (Reinforcing)**	Availability of water resources → Environmental awareness → Effective regulation and governance → Production of desalinated and reclaimed water → Volume of desalinated and reclaimed water → Use of desalinated and reclaimed water → Water use efficiency → Cost of the water cycle → Water price → Water use by agri-food industry and other sectors → Economic activity → Soil quality → Quality of surface water and groundwater → Availability of water resources
**BW20a (Balancing)**	Availability of water resources → Environmental awareness → Effective regulation and governance → Social acceptance of alternative water sources → Use of desalinated and reclaimed water → Water use efficiency → Volume of surface water and groundwater → Availability of water resources
**BW20b (Balancing)**	Availability of water resources → Environmental awareness → Effective regulation and governance → Social acceptance of alternative water sources → Use of desalinated and reclaimed water → Production of desalinated and reclaimed water → Volume of desalinated and reclaimed water → Availability of water resources
**BW20c (Balancing)**	Availability of water resources → Environmental awareness → Effective regulation and governance → Social acceptance of alternative water sources → Use of desalinated and reclaimed water → Production of desalinated and reclaimed water → Energy demand → Energy price → Cost of the water cycle → Economic activity → Quality of surface water and groundwater → Availability of water resources
**BW20d (Balancing)**	Availability of water resources → Environmental awareness → Effective regulation and governance → Social acceptance of alternative water sources → Use of desalinated and reclaimed water → Production of desalinated and reclaimed water → Energy demand → Energy price → Cost of the water cycle → Water use by agri-food industry and other sectors → Volume of surface water and groundwater → Availability of water resources
**BW20e (Balancing)**	Availability of water resources → Environmental awareness → Effective regulation and governance → Social acceptance of alternative water sources → Use of desalinated and reclaimed water → Production of desalinated and reclaimed water → Energy demand → Energy price → Cost of the water cycle → Water use by agri-food industry and other sectors → Economic activity → Quality of surface water and groundwater → Availability of water resources
**BW20f (Balancing)**	Availability of water resources → Environmental awareness → Effective regulation and governance → Social acceptance of alternative water sources → Use of desalinated and reclaimed water → Production of desalinated and reclaimed water → Energy demand → Energy price → Cost of the water cycle → Economic activity → Soil quality → Quality of surface water and groundwater → Availability of water resources
**BW20g (Balancing)**	Availability of water resources → Environmental awareness → Effective regulation and governance → Social acceptance of alternative water sources → Use of desalinated and reclaimed water → Production of desalinated and reclaimed water → Energy demand → Energy price → Cost of the water cycle → Water price → Water use by agri-food industry and other sectors → Volume of surface water and groundwater → Availability of water resources
**BW20h (Balancing)**	Availability of water resources → Environmental awareness → Effective regulation and governance → Social acceptance of alternative water sources → Use of desalinated and reclaimed water → Production of desalinated and reclaimed water → Energy demand → Energy price → Cost of the water cycle → Economic activity → Water use by agri-food industry and other sectors → Volume of surface water and groundwater → Availability of water resources

**BW20i (Balancing)**	Availability of water resources → Environmental awareness → Effective regulation and governance → Social acceptance of alternative water sources → Use of desalinated and reclaimed water → Production of desalinated and reclaimed water → Energy demand → Energy price → Cost of the water cycle → Water use by agri-food industry and other sectors → Economic activity → Soil quality → Quality of surface water and groundwater → Availability of water resources
**BW20j (Balancing)**	Availability of water resources → Environmental awareness → Effective regulation and governance → Social acceptance of alternative water sources → Use of desalinated and reclaimed water → Production of desalinated and reclaimed water → Energy demand → Energy price → Cost of the water cycle → Water price → Water use by agri-food industry and other sectors → Economic activity → Quality of surface water and groundwater → Availability of water resources
**BW20k (Balancing)**	Availability of water resources → Environmental awareness → Effective regulation and governance → Social acceptance of alternative water sources → Use of desalinated and reclaimed water → Production of desalinated and reclaimed water → Energy demand → Energy price → Cost of the water cycle → Water price → Water use by agri-food industry and other sectors → Economic activity → Soil quality → Quality of surface water and groundwater → Availability of water resources
**RW15a (Reinforcing)**	Availability of water resources → Environmental awareness → Effective regulation and governance → Social acceptance of alternative water sources → Use of desalinated and reclaimed water → Volume of desalinated and reclaimed water → Availability of water resources
**RW15b (Reinforcing)**	Availability of water resources → Environmental awareness → Effective regulation and governance → Social acceptance of alternative water sources → Use of desalinated and reclaimed water → Water use efficiency → Cost of the water cycle → Water use by agri-food industry and other sectors → Volume of surface water and groundwater → Availability of water resources
**RW15c (Reinforcing)**	Availability of water resources → Environmental awareness → Effective regulation and governance → Social acceptance of alternative water sources → Use of desalinated and reclaimed water → Water use efficiency → Cost of the water cycle → Economic activity → Quality of surface water and groundwater → Availability of water resources
**RW15d (Reinforcing)**	Availability of water resources → Environmental awareness → Effective regulation and governance → Social acceptance of alternative water sources → Use of desalinated and reclaimed water → Water use efficiency → Cost of the water cycle → Water price → Water use by agri-food industry and other sectors → Volume of surface water and groundwater → Availability of water resources
**RW15e (Reinforcing)**	Availability of water resources → Environmental awareness → Effective regulation and governance → Social acceptance of alternative water sources → Use of desalinated and reclaimed water → Water use efficiency → Cost of the water cycle → Economic activity → Water use by agri-food industry and other sectors → Volume of surface water and groundwater → Availability of water resources
**RW15f (Reinforcing)**	Availability of water resources → Environmental awareness → Effective regulation and governance → Social acceptance of alternative water sources → Use of desalinated and reclaimed water → Water use efficiency → Cost of the water cycle → Economic activity → Soil quality → Quality of surface water and groundwater → Availability of water resources
**RW15g (Reinforcing)**	Availability of water resources → Environmental awareness → Effective regulation and governance → Social acceptance of alternative water sources → Use of desalinated and reclaimed water → Water use efficiency → Cost of the water cycle → Water use by agri-food industry and other sectors → Economic activity → Quality of surface water and groundwater → Availability of water resources

**RW15h (Reinforcing)**	Availability of water resources → Environmental awareness → Effective regulation and governance → Social acceptance of alternative water sources → Use of desalinated and reclaimed water → Water use efficiency → Cost of the water cycle → Water use by agri-food industry and other sectors → Economic activity → Soil quality → Quality of surface water and groundwater → Availability of water resources
**RW15i (Reinforcing)**	Availability of water resources → Environmental awareness → Effective regulation and governance → Social acceptance of alternative water sources → Use of desalinated and reclaimed water → Water use efficiency → Cost of the water cycle → Water price → Water use by agri-food industry and other sectors → Economic activity → Quality of surface water and groundwater → Availability of water resources
**RW15j (Reinforcing)**	Availability of water resources → Environmental awareness → Effective regulation and governance → Social acceptance of alternative water sources → Use of desalinated and reclaimed water → Water use efficiency → Cost of the water cycle → Water price → Water use by agri-food industry and other sectors → Economic activity → Soil quality → Quality of surface water and groundwater → Availability of water resources
**BW21a (Balancing)**	Availability of water resources → Environmental awareness → Production of desalinated and reclaimed water → Volume of desalinated and reclaimed water → Use of desalinated and reclaimed water → Water use efficiency → Volume of surface water and groundwater → Availability of water resources
**BW21b (Balancing)**	Availability of water resources → Environmental awareness → Production of desalinated and reclaimed water → Energy demand → Energy price → Cost of the water cycle → Water use by agri-food industry and other sectors → Volume of surface water and groundwater → Availability of water resources
**BW21c (Balancing)**	Availability of water resources → Environmental awareness → Production of desalinated and reclaimed water → Energy demand → Energy price → Cost of the water cycle → Economic activity → Quality of surface water and groundwater → Availability of water resources
**BW21d (Balancing)**	Availability of water resources → Environmental awareness → Production of desalinated and reclaimed water → Energy demand → Energy price → Cost of the water cycle → Economic activity → Soil quality → Quality of surface water and groundwater → Availability of water resources
**BW21e (Balancing)**	Availability of water resources → Environmental awareness → Production of desalinated and reclaimed water → Energy demand → Energy price → Cost of the water cycle → Economic activity → Water use by agri-food industry and other sectors → Volume of surface water and groundwater → Availability of water resources
**BW21f (Balancing)**	Availability of water resources → Environmental awareness → Production of desalinated and reclaimed water → Energy demand → Energy price → Cost of the water cycle → Water price → Water use by agri-food industry and other sectors → Volume of surface water and groundwater → Availability of water resources
**BW21g (Balancing)**	Availability of water resources → Environmental awareness → Production of desalinated and reclaimed water → Energy demand → Energy price → Cost of the water cycle → Water use by agri-food industry and other sectors → Economic activity → Quality of surface water and groundwater → Availability of water resources
**BW21h (Balancing)**	Availability of water resources → Environmental awareness → Production of desalinated and reclaimed water → Energy demand → Energy price → Cost of the water cycle → Water price → Water use by agri-food industry and other sectors → Economic activity → Quality of surface water and groundwater → Availability of water resources
**BW21i (Balancing)**	Availability of water resources → Environmental awareness → Production of desalinated and reclaimed water → Energy demand → Energy price → Cost of the water cycle → Water use by agri-food industry and other sectors → Economic activity → Soil quality → Quality of surface water and groundwater → Availability of water resources

**BW21j** **(Balancing)**	Availability of water resources → Environmental awareness → Production of desalinated and reclaimed water → Energy demand → Energy price → Cost of the water cycle → Water price → Water use by agri-food industry and other sectors → Economic activity → Soil quality → Quality of surface water and groundwater → Availability of water resources
**RW16a** **(Reinforcing)**	Availability of water resources → Environmental awareness → Production of desalinated and reclaimed water → Volume of desalinated and reclaimed water → Use of desalinated and reclaimed water → Water use efficiency → Cost of the water cycle → Economic activity → Quality of surface water and groundwater → Availability of water resources
**RW16b** **(Reinforcing)**	Availability of water resources → Environmental awareness → Production of desalinated and reclaimed water → Volume of desalinated and reclaimed water → Use of desalinated and reclaimed water → Water use efficiency → Cost of the water cycle → Water use by agri-food industry and other sectors → Volume of surface water and groundwater → Availability of water resources
**RW16c** **(Reinforcing)**	Availability of water resources → Environmental awareness → Production of desalinated and reclaimed water → Volume of desalinated and reclaimed water → Use of desalinated and reclaimed water → Water use efficiency → Cost of the water cycle → Water use by agri-food industry and other sectors → Economic activity → Quality of surface water and groundwater → Availability of water resources
**RW16d** **(Reinforcing)**	Availability of water resources → Environmental awareness → Production of desalinated and reclaimed water → Volume of desalinated and reclaimed water → Use of desalinated and reclaimed water → Water use efficiency → Cost of the water cycle → Economic activity → Soil quality → Quality of surface water and groundwater → Availability of water resources
**RW16e** **(Reinforcing)**	Availability of water resources → Environmental awareness → Production of desalinated and reclaimed water → Volume of desalinated and reclaimed water → Use of desalinated and reclaimed water → Water use efficiency → Cost of the water cycle → Economic activity → Water use by agri-food industry and other sectors → Volume of surface water and groundwater → Availability of water resources
**RW16f** **(Reinforcing)**	Availability of water resources → Environmental awareness → Production of desalinated and reclaimed water → Volume of desalinated and reclaimed water → Use of desalinated and reclaimed water → Water use efficiency → Cost of the water cycle → Water price → Water use by agri-food industry and other sectors → Volume of surface water and groundwater → Availability of water resources
**RW16g** **(Reinforcing)**	Availability of water resources → Environmental awareness → Production of desalinated and reclaimed water → Volume of desalinated and reclaimed water → Use of desalinated and reclaimed water → Water use efficiency → Cost of the water cycle → Water use by agri-food industry and other sectors → Economic activity → Soil quality → Quality of surface water and groundwater → Availability of water resources
**RW16h** **(Reinforcing)**	Availability of water resources → Environmental awareness → Production of desalinated and reclaimed water → Volume of desalinated and reclaimed water → Use of desalinated and reclaimed water → Water use efficiency → Cost of the water cycle → Water price → Water use by agri-food industry and other sectors → Economic activity → Quality of surface water and groundwater → Availability of water resources
**RW16i** **(Reinforcing)**	Availability of water resources → Environmental awareness → Production of desalinated and reclaimed water → Volume of desalinated and reclaimed water → Use of desalinated and reclaimed water → Water use efficiency → Cost of the water cycle → Water price → Water use by agri-food industry and other sectors → Economic activity → Soil quality → Quality of surface water and groundwater → Availability of water resources
**BW22a** **(Balancing)**	Availability of water resources → Environmental awareness → Social acceptance of alternative water sources → Use of desalinated and reclaimed water → Production of desalinated and reclaimed water → Volume of desalinated and reclaimed water → Availability of water resources

**BW22b** **(Balancing)**	Availability of water resources → Environmental awareness → Social acceptance of alternative water sources → Use of desalinated and reclaimed water → Water use efficiency → Volume of surface water and groundwater → Availability of water resources
**BW22c** **(Balancing)**	Availability of water resources → Environmental awareness → Social acceptance of alternative water sources → Use of desalinated and reclaimed water → Production of desalinated and reclaimed water → Energy demand → Energy price → Cost of the water cycle → Water use by agri-food industry and other sectors → Volume of surface water and groundwater → Availability of water resources
**BW22d** **(Balancing)**	Availability of water resources → Environmental awareness → Social acceptance of alternative water sources → Use of desalinated and reclaimed water → Production of desalinated and reclaimed water → Energy demand → Energy price → Cost of the water cycle → Economic activity → Quality of surface water and groundwater → Availability of water resources
**BW22e** **(Balancing)**	Availability of water resources → Environmental awareness → Social acceptance of alternative water sources → Use of desalinated and reclaimed water → Production of desalinated and reclaimed water → Energy demand → Energy price → Cost of the water cycle → Economic activity → Water use by agri-food industry and other sectors → Volume of surface water and groundwater → Availability of water resources
**BW22f** **(Balancing)**	Availability of water resources → Environmental awareness → Social acceptance of alternative water sources → Use of desalinated and reclaimed water → Production of desalinated and reclaimed water → Energy demand → Energy price → Cost of the water cycle → Water use by agri-food industry and other sectors → Economic activity → Quality of surface water and groundwater → Availability of water resources
**BW22g** **(Balancing)**	Availability of water resources → Environmental awareness → Social acceptance of alternative water sources → Use of desalinated and reclaimed water → Production of desalinated and reclaimed water → Energy demand → Energy price → Cost of the water cycle → Water price → Water use by agri-food industry and other sectors → Volume of surface water and groundwater → Availability of water resources
**BW22h** **(Balancing)**	Availability of water resources → Environmental awareness → Social acceptance of alternative water sources → Use of desalinated and reclaimed water → Production of desalinated and reclaimed water → Energy demand → Energy price → Cost of the water cycle → Economic activity → Soil quality → Quality of surface water and groundwater → Availability of water resources
**BW22i** **(Balancing)**	Availability of water resources → Environmental awareness → Social acceptance of alternative water sources → Use of desalinated and reclaimed water → Production of desalinated and reclaimed water → Energy demand → Energy price → Cost of the water cycle → Water use by agri-food industry and other sectors → Economic activity → Soil quality → Quality of surface water and groundwater → Availability of water resources
**BW22j** **(Balancing)**	Availability of water resources → Environmental awareness → Social acceptance of alternative water sources → Use of desalinated and reclaimed water → Production of desalinated and reclaimed water → Energy demand → Energy price → Cost of the water cycle → Water price → Water use by agri-food industry and other sectors → Economic activity → Quality of surface water and groundwater → Availability of water resources
**BW22k** **(Balancing)**	Availability of water resources → Environmental awareness → Social acceptance of alternative water sources → Use of desalinated and reclaimed water → Production of desalinated and reclaimed water → Energy demand → Energy price → Cost of the water cycle → Water price → Water use by agri-food industry and other sectors → Economic activity → Soil quality → Quality of surface water and groundwater → Availability of water resources
**RW17a** **(Reinforcing)**	Availability of water resources → Environmental awareness → Social acceptance of alternative water sources → Use of desalinated and reclaimed water → Volume of desalinated and reclaimed water → Availability of water resources

**RW17b** **(Reinforcing)**	Availability of water resources → Environmental awareness → Social acceptance of alternative water sources → Use of desalinated and reclaimed water → Water use efficiency → Cost of the water cycle → Economic activity → Quality of surface water and groundwater → Availability of water resources
**RW17c** **(Reinforcing)**	Availability of water resources → Environmental awareness → Social acceptance of alternative water sources → Use of desalinated and reclaimed water → Water use efficiency → Cost of the water cycle → Water use by agri-food industry and other sectors → Volume of surface water and groundwater → Availability of water resources
**RW17d** **(Reinforcing)**	Availability of water resources → Environmental awareness → Social acceptance of alternative water sources → Use of desalinated and reclaimed water → Water use efficiency → Cost of the water cycle → Water use by agri-food industry and other sectors → Economic activity → Quality of surface water and groundwater → Availability of water resources
**RW17e** **(Reinforcing)**	Availability of water resources → Environmental awareness → Social acceptance of alternative water sources → Use of desalinated and reclaimed water → Water use efficiency → Cost of the water cycle → Economic activity → Water use by agri-food industry and other sectors → Volume of surface water and groundwater → Availability of water resources
**RW17f** **(Reinforcing)**	Availability of water resources → Environmental awareness → Social acceptance of alternative water sources → Use of desalinated and reclaimed water → Water use efficiency → Cost of the water cycle → Water price → Water use by agri-food industry and other sectors → Volume of surface water and groundwater → Availability of water resources
**RW17g** **(Reinforcing)**	Availability of water resources → Environmental awareness → Social acceptance of alternative water sources → Use of desalinated and reclaimed water → Water use efficiency → Cost of the water cycle → Economic activity → Soil quality → Quality of surface water and groundwater → Availability of water resources
**RW17h** **(Reinforcing)**	Availability of water resources → Environmental awareness → Social acceptance of alternative water sources → Use of desalinated and reclaimed water → Water use efficiency → Cost of the water cycle → Water price → Water use by agri-food industry and other sectors → Economic activity → Quality of surface water and groundwater → Availability of water resources
**RW17i** **(Reinforcing)**	Availability of water resources → Environmental awareness → Social acceptance of alternative water sources → Use of desalinated and reclaimed water → Water use efficiency → Cost of the water cycle → Water use by agri-food industry and other sectors → Economic activity → Soil quality → Quality of surface water and groundwater → Availability of water resources
**RW17j** **(Reinforcing)**	Availability of water resources → Environmental awareness → Social acceptance of alternative water sources → Use of desalinated and reclaimed water → Water use efficiency → Cost of the water cycle → Water price → Water use by agri-food industry and other sectors → Economic activity → Soil quality → Quality of surface water and groundwater → Availability of water resources
**BW23** **(Balancing)**	Availability of water resources → Water use by agri-food industry and other sectors → Volume of surface water and groundwater → Availability of water resources
**BW24a** **(Balancing)**	Availability of water resources → Water use by agri-food industry and other sectors → Economic activity → Quality of surface water and groundwater → Availability of water resources
**BW24b** **(Balancing)**	Availability of water resources → Water use by agri-food industry and other sectors → Economic activity → Soil quality → Quality of surface water and groundwater → Availability of water resources
**BW25a** **(Balancing)**	Availability of water resources → Environmental awareness → Illegal abstraction of water → Volume of surface water and groundwater → Availability of water resources

**BW25b** **(Balancing)**	Availability of water resources → Environmental awareness → Illegal abstraction of water → Marine intrusion in aquifers → Quality of surface water and groundwater → Availability of water resources
**BW26** **(Balancing)**	Energy demand → Energy price → Energy demand
**RW18** **(Reinforcing)**	Economic activity → Water use by agri-food industry and other sectors → Economic activity
