# Supplementary material for: Systems Modeling of the Water-Energy-Food-Ecosystems Nexus: Insights from a Region Facing Structural Water Scarcity in Southern Spain
Source: Environ Manage. 2024 Sep 13;74(6):1045–62. doi: 10.1007/s00267-024-02037-6 (PMC11549115; doi:10.1007/s00267-024-02037-6)
Supplement: Supplementary file 4 — Appendix 4 [file 267_2024_2037_MOESM4_ESM.pdf]

Environmental Management

Supplementary Information

Appendix 4

Systems Modeling of the Water-Energy-Food-Ecosystems Nexus: Insights from a Region Facing Structural Water-Scarcity in Southern Spain

Antonio R. Hurtado[1,*], Enrique Mesa-Pérez[2], Julio Berbel[1]

[1] Water, Environmental and Agricultural Resources Economics (WEARE) Research Group, Department of Agricultural Economics, University of Cordoba, Campus Rabanales Building C5, 14014 Córdoba, Spain

[2] Departamento de Economía Financiera y Contabilidad, Universidad Loyola Andalucía, 41704 Dos Hermanas (Sevilla), Spain

*Corresponding author (es2rohuc@uco.es)

Water-Energy-Food-Ecosystem (WEFE) nexus

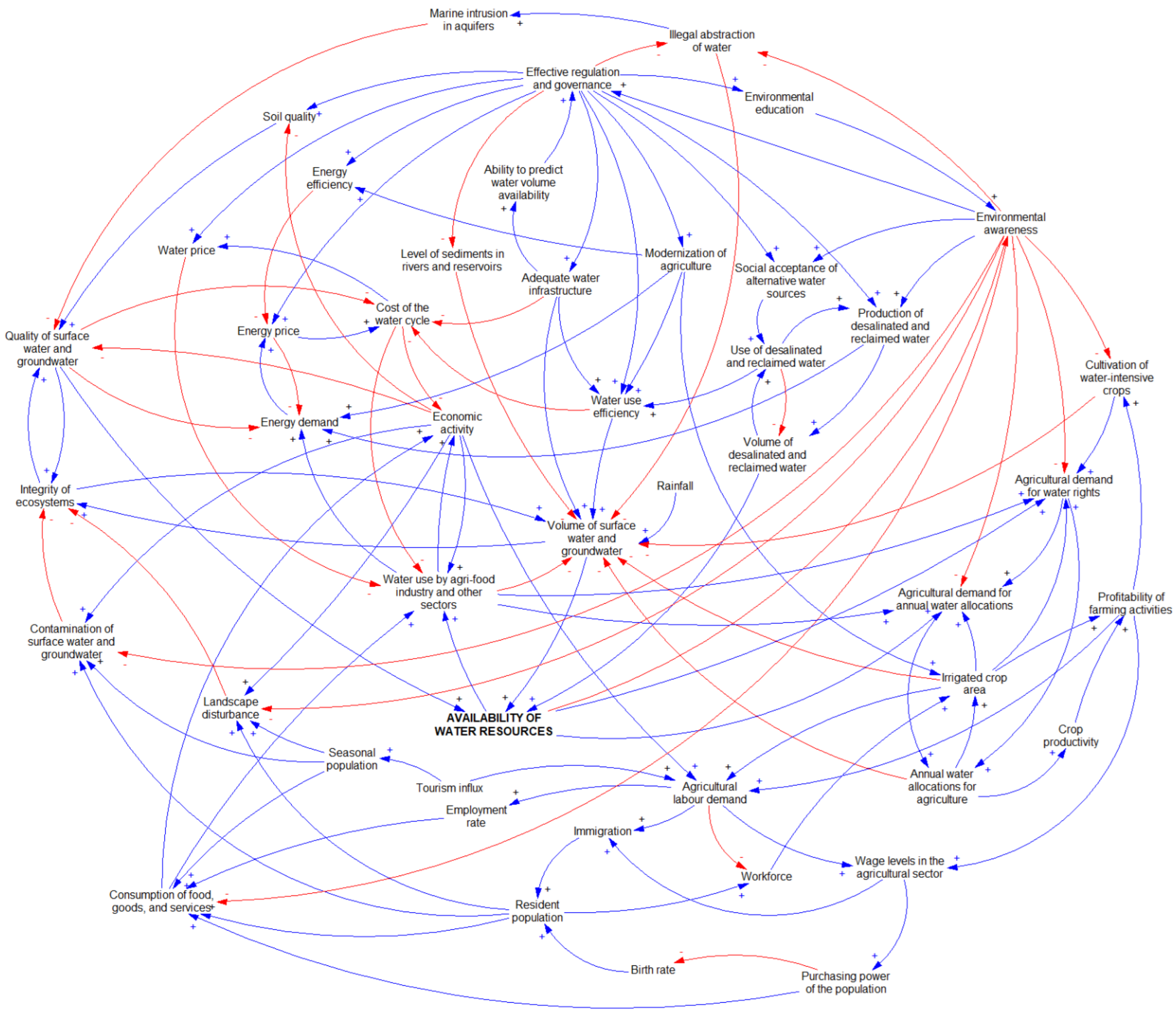

Figure SI-4. Causal loop diagram displaying the dynamics hypothesized to govern the WEFE nexus in Axarquía.

Table SI-4.1. List of nodes of the integrated CLD of the WEFE nexus in Axarquia.

ID	Variable	ID	Variable
1	Ability to predict water volume availability	25	Irrigated crop area
2	Adequate water infrastructure	26	Landscape disturbance
3	Agricultural demand for annual water allocations	27	Level of sediments in rivers and reservoirs
4	Agricultural demand for water rights	28	Marine intrusion in aquifers
5	Agricultural labour demand	29	Modernization of agriculture
6	Annual water allocations for agriculture	30	Production of desalinated and reclaimed water
7	Availability of water resources	31	Profitability of farming activities
8	Birth rate	32	Purchasing power of the population
9	Consumption of food, goods, and services	33	Quality of surface water and groundwater
10	Contamination of surface water and groundwater	34	Rainfall
11	Cost of the water cycle	35	Resident population
12	Crop productivity	36	Seasonal population
13	Cultivation of water-intensive crops	37	Social acceptance of alternative water sources
14	Economic activity	38	Soil quality
15	Effective regulation and governance	39	Tourism influx
16	Employment rate	40	Use of desalinated and reclaimed water
17	Energy demand	41	Volume of desalinated and reclaimed water
18	Energy efficiency	42	Volume of surface water and groundwater
19	Energy price	43	Wage levels in the agricultural sector
20	Environmental awareness	44	Water price
21	Environmental education	45	Water use by agri-food industry and other sectors
22	Illegal abstraction of water	46	Water use efficiency
23	Immigration	47	Workforce
24	Integrity of ecosystems		

Table SI-4.2. List of edges of the integrated CLD of the WEFE nexus in Axarquia.

Source	Target	Source	Target	Source	Target	Source	Target
1	15	14	10	20	37	34	42
2	1	14	26	21	20	35	9
2	11	14	33	22	28	35	10
2	42	14	38	22	42	35	26
2	46	14	45	23	35	35	47
3	6	15	2	24	33	36	9
4	3	15	18	24	42	36	10
4	6	15	19	25	3	36	26
5	16	15	21	25	4	37	40
5	23	15	22	25	5	38	33
5	43	15	27	25	31	39	5
5	47	15	29	25	42	39	36
6	12	15	30	26	24	40	30
6	25	15	37	27	42	40	41
6	42	15	38	28	33	40	46
7	3	15	44	29	17	41	7
7	4	15	46	29	18	41	40
7	20	16	9	29	25	42	7
7	45	17	19	29	46	42	24
8	35	18	19	30	17	43	23
9	14	19	11	30	41	43	32
9	45	20	3	31	5	44	45
10	24	20	4	31	13	45	3
11	14	20	9	31	43	45	4
11	44	20	10	32	8	45	14
11	45	20	13	32	9	45	17
12	31	20	15	33	7	45	42
13	4	20	22	33	11	46	11
13	42	20	26	33	17	46	42
14	5	20	30	33	24	47	25

Table SI-4.3. Degree, closeness, eigenvector, and betweenness centralities in Axarquia's WEFE nexus.

ID	Variable	In-Degree	Out-Degree	Degree Centrality	Closeness Centrality	Eigenvector Centrality	Betweenness Centrality
1	Ability to predict water volume availability	1	1	2	0.288590604	0.022471907	14.50000000
2	Adequate water infrastructure	1	4	5	0.313868613	0.042348354	48.26666667
3	Agricultural demand for annual water allocations	5	1	6	0.216080402	0.862391747	65.44444444
4	Agricultural demand for water rights	5	2	7	0.218274112	0.682508599	83.44444444
5	Agricultural labour demand	4	4	8	0.228723404	0.327678948	312.14682540
6	Annual water allocations for agriculture	2	3	5	0.272151899	0.523132810	184.88888889
7	Availability of water resources	3	4	7	0.349593496	0.600948087	855.47777778
8	Birth rate	1	1	2	0.190265487	0.030663260	5.666666667
9	Consumption of food, goods, and services	5	2	7	0.257485030	0.210372950	229.48571429
10	Contamination of surface water and groundwater	4	1	5	0.197247706	0.305691255	38.01904762
11	Cost of the water cycle	4	3	7	0.260606061	0.380217456	296.48888889
12	Crop productivity	1	1	2	0.200934579	0.168944632	20.04444444
13	Cultivation of water-intensive crops	2	2	4	0.241573034	0.138338071	53.53333333
14	Economic activity	3	6	9	0.318518519	0.482151934	425.95396825
15	Effective regulation and governance	2	12	14	0.398148148	0.097172796	379.63333333
16	Employment rate	1	1	2	0.207729469	0.122384515	25.73333333
17	Energy demand	4	1	5	0.175510204	0.529160695	43.30000000
18	Energy efficiency	2	1	3	0.178423237	0.064820261	0.50000000
19	Energy price	3	1	4	0.209756098	0.263233604	95.06666667
20	Environmental awareness	2	10	12	0.417475728	0.229272498	713.83333333
21	Environmental education	1	1	2	0.298611111	0.042348354	12.66666667
22	Illegal abstraction of water	2	2	4	0.228723404	0.123936694	48.30000000
23	Immigration	2	1	3	0.188596491	0.192613577	58.66666667
24	Integrity of ecosystems	4	2	6	0.240223464	0.780475004	186.93809524
25	Irrigated crop area	3	5	8	0.290540541	0.258210675	200.09841270
26	Landscape disturbance	4	1	5	0.197247706	0.305691255	38.01904762
27	Level of sediments in rivers and reservoirs	1	1	2	0.219387755	0.042348354	1.23333333

28	Marine intrusion in aquifers	1	1	2	0.241573034	0.051325150	10.23333333
29	Modernization of agriculture	1	4	5	0.277419355	0.042348354	26.73333333
30	Production of desalinated and reclaimed water	3	2	5	0.228723404	0.172159760	51.83333333
31	Profitability of farming activities	2	3	5	0.245714286	0.148983812	72.50000000
32	Purchasing power of the population	1	2	3	0.220512821	0.070229062	81.56666667
33	Quality of surface water and groundwater	4	4	8	0.309352518	0.548472430	347.59523810
34	Rainfall	0	1	1	0.218905473	0.000000000	0.00000000
35	Resident population	2	4	6	0.227513228	0.092383621	104.33333333
36	Seasonal population	1	3	4	0.214634146	0.003579768	28.46428571
37	Social acceptance of alternative water sources	2	1	3	0.205741627	0.123936694	43.00000000
38	Soil quality	2	1	3	0.240223464	0.216027535	3.13333333
39	Tourism influx	0	2	2	0.206422018	0.000000000	0.00000000
40	Use of desalinated and reclaimed water	2	3	5	0.252941176	0.105583878	48.83333333
41	Volume of desalinated and reclaimed water	2	2	4	0.277419355	0.120714358	75.66666667
42	Volume of surface water and groundwater	10	2	12	0.273885350	1.000000000	577.21587302
43	Wage levels in the agricultural sector	2	2	4	0.193693694	0.179134247	97.56666667
44	Water price	2	1	3	0.236263736	0.186978668	5.16666667
45	Water use by agri-food industry and other sectors	5	5	10	0.300699301	0.691170273	310.18730159
46	Water use efficiency	4	2	6	0.260606061	0.135515234	54.43333333
47	Workforce	2	1	3	0.227513228	0.164586684	77.18730159
